# Topogenic sequence recognition at TIM complexes revealed by a stendomycin-bound structure

**DOI:** 10.1038/s41589-026-02304-z

**Published:** 2026-08-26

**Authors:** Yuanyuan Chen, Antony Lurie, Kevin Wu, Kihong Nam, Samantha N. Garcia, Nathaniel W. M. Dempsey, Esben B. Svenningsen, Thomas Tørring, Thomas B. Poulsen, Alban Ordureau, Eunyong Park

**Affiliations:** 1 Department of Molecular and Cell Biology, University of California, Berkeley, CA USA; 2 California Institute for Quantitative Biosciences, University of California, Berkeley, CA USA; 3 Cell Biology Program, Sloan Kettering Institute, Memorial Sloan Kettering Cancer Center, New York, NY USA; 4 Department of Chemistry, Aarhus University, Aarhus, Denmark; 5 Department of Biological and Chemical Engineering, Aarhus University, Aarhus, Denmark

**Keywords:** Structural biology, Membrane proteins, Mechanism of action

## Abstract

In the mitochondrial inner membrane (IM), topogenesis of imported proteins is mediated by TIM23 and TIM22 complexes. TIM23 translocates soluble polypeptides across the IM into the matrix, whereas TIM22 inserts polytopic membrane proteins into the IM. Although functionally distinct, both rely on homologous subunits, Tim17 in TIM23 and Tim22 in TIM22. The underlying mechanisms, however, remain elusive. Here we use structural and functional approaches with yeast Tim17, Tim22 and the TIM23 inhibitor stendomycin. Cryogenic-electron microscopy shows that stendomycin binds to the protein translocation cavity of Tim17, mimicking α-helical topogenic sequences. While Tim22 does not bind stendomycin, a single mutation in its equivalent cavity suffices to enable binding. The cavities of Tim17 and Tim22 are largely interchangeable without disrupting their functions. Lastly, stendomycin triggers a collapse of the membrane potential, likely via its Tim17- or Tim22-dependent translocation across the IM. These findings reveal a mechanistic overlap between protein translocases and insertases.

**Figure:**
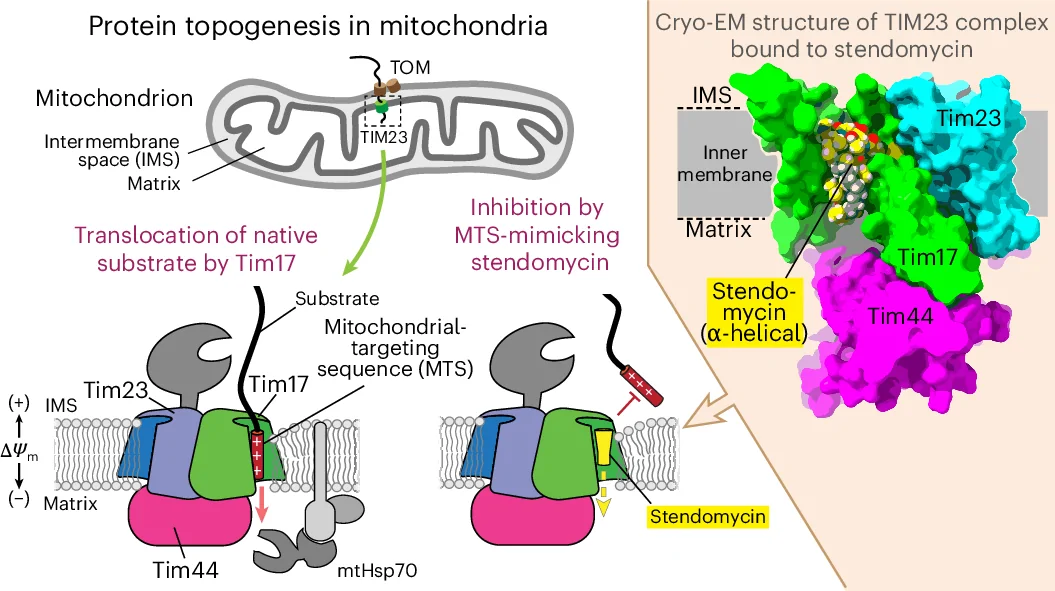

## Abstract (web summary)

The mitochondrial inner membrane complexes TIM23 and TIM22 mediate distinct protein topogenesis functions in polypeptide import. The authors reveal their mechanistic overlap in topogenic sequence recognition by cryo-EM and functional studies using the yeast system and the stendomycin inhibitor.

## Main

Approximately 99% of mitochondrial proteins are synthesized in the cytosol and imported into the organelle (for reviews, see refs. ^1–5^). These precursor proteins are first transported in an unfolded state across the mitochondrial outer membrane (OM) through the translocase of the outer membrane (TOM) complex. Most proteins are further translocated across or integrated into the inner membrane (IM) by the two distinct translocase of the inner membrane (TIM) complexes, TIM23 and TIM22. As most mitochondrial proteins are destined for the matrix or IM, TIM23 and TIM22 are indispensable for mitochondrial biogenesis and function.

TIM23 functions mainly as a protein translocase, whereas TIM22 acts as a membrane protein insertase. TIM23 transports soluble proteins across the IM into the matrix, typically in coordination with TOM, as most polypeptides simultaneously move across the OM and IM in an extended conformation spanning both TOM and TIM23 (refs. ^6–8^). TIM23 also inserts certain hydrophobic segments into the IM through a stop-transfer mechanism, in which a hydrophobic segment within the polypeptide halts further translocation and is released laterally into the lipid bilayer^9,10^. By contrast, TIM22 integrates multi-pass membrane proteins lacking extensive soluble domains^11,12^. These proteins may first be fully translocated into the intermembrane space (IMS) via TOM, where they are stabilized by small TIM complexes, before TIM22-mediated insertion^13–15^.

TIM23 and TIM22 also differ in the targeting (topogenic) sequences they recognize. TIM23 substrates generally possess a short, positively charged, amphipathic N-terminal sequence called the mitochondrial targeting sequence (MTS) or presequence^16–18^. In contrast, TIM22 substrates lack an MTS and presumably rely on hydrophobic transmembrane (TM) segments for targeting^11,15,19^. The mechanisms underlying the recognition of these sequences and client engagement remain poorly understood.

Although functionally distinct, their core subunits, Tim17 in TIM23 and Tim22 in TIM22, are evolutionarily related. Cryogenic-electron microscopy (cryo-EM) studies have revealed that Tim17 and Tim22 share notably similar architectures, including a lipid-facing hydrophilic cavity, which could explain their insertase functions^20–22^. While Tim17 can form a channel-like structure by associating with the transmembrane protein Mgr2 (Romo1 in humans)—a feature more consistent with translocase activity—Mgr2 is dispensable for protein translocation^20,23–26^. This suggests that the translocase and insertase functions of TIM23 and TIM22 are largely mediated by the same insertase-like structure. Although both complexes were historically considered channels^27–31^, these structures indicate that they perform their functions using a single copy of Tim17 or Tim22 without strictly forming a channel. In addition, their structural similarity hints at a potential mechanistic overlap, despite the structural and functional distinction between protein translocases and insertases in general.

At present, it remains unclear how Tim17 and Tim22 mediate protein topogenesis. The low sequence identity (~20%) between Tim17 and Tim22 results in substantial surface differences, which may account for their functional divergence. These differences may also explain the chemoproteomic selectivity of stendomycin, a natural product known to specifically inhibit TIM23, but not TIM22, in yeast^32^. Understanding whether and how the structural differences between Tim17 and Tim22 underlie stendomycin’s selectivity could provide valuable insights into their functional specialization.

Here we addressed these questions by determining the structure of stendomycin-bound TIM23 and performing mutational studies. We show that stendomycin inhibits TIM23-mediated protein translocation by directly occluding the translocation pathway in Tim17. Notably, stendomycin adopts an α-helical structure, mimicking topogenic sequences, such as MTSs and TMs. With the introduction of a single amino acid substitution into its cavity, Tim22 could also be targeted by stendomycin. Moreover, the cavities of Tim17 and Tim22 are largely interchangeable, sustaining their respective functions. Lastly, we found that stendomycin depletes the mitochondrial membrane potential, likely due to its translocation across the IM upon binding to Tim17 or Tim22 and upregulates certain mitochondrial lipid transporters (Ups1 and Ups2) as a potential stress response.

## Results

### Cryo-EM analysis of stendomycin-bound TIM23

**Fig. 1:**
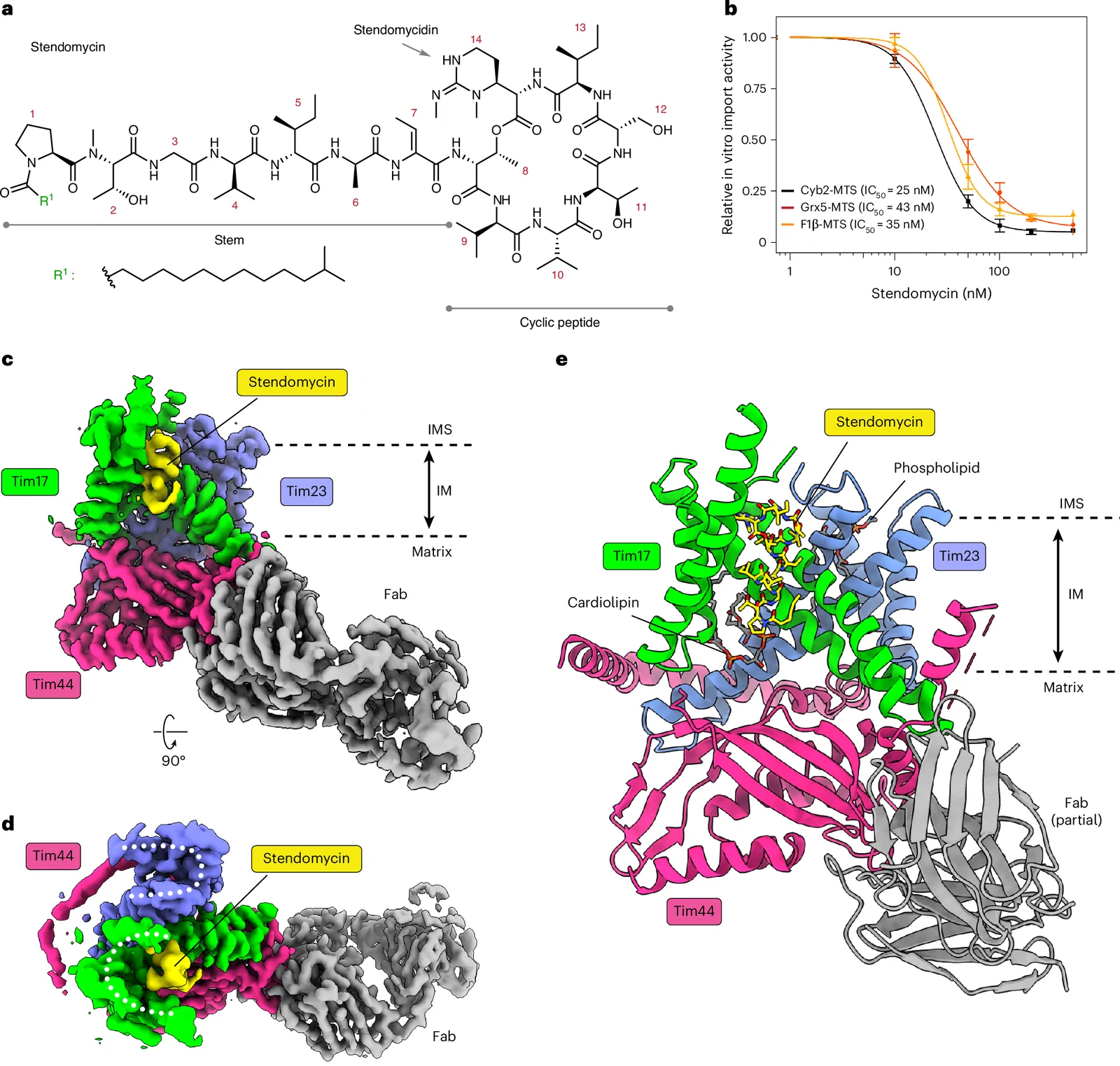
Cryo-EM structure of the stendomycin-bound core TIM23 complex from *S. cerevisiae*. **a**, Chemical structure of stendomycin. Note that there are different congeners of stendomycin with minor variations in the acyl chain (R_1_; C_11_H_23_, C_12_H_25_ or C_13_H_27_) and residues 5 and 13 (D-alloisoleucine or D-valine). Shown here is stendomycin I. **b**, Inhibitory effect of stendomycin on in vitro protein import into isolated mitochondria. Shown are means ± s.e.m. of three independent experiments, and IC_50_ values were derived from dose–response curve fitting (Methods). See Extended Data Fig. 1b for representative immunoblot images used for quantification. **c**, Side view of the 3.2-Å-resolution cryo-EM map of the stendomycin-bound core TIM23 complex. **d**, As in **c**, but showing the top view (view from IMS). The back-to-back arrangement of the concave cavities of the Tim17–Tim23 heterodimer is indicated by white dotted lines. **e**, Atomic model of the stendomycin-bound TIM23 complex (side view). In addition to stendomycin, copurified cardiolipin and phospholipid (shown in a stick representation) were also observed as seen in the apo structure (PDB 8SCX). Source data

**Extended Data Fig. 1:**
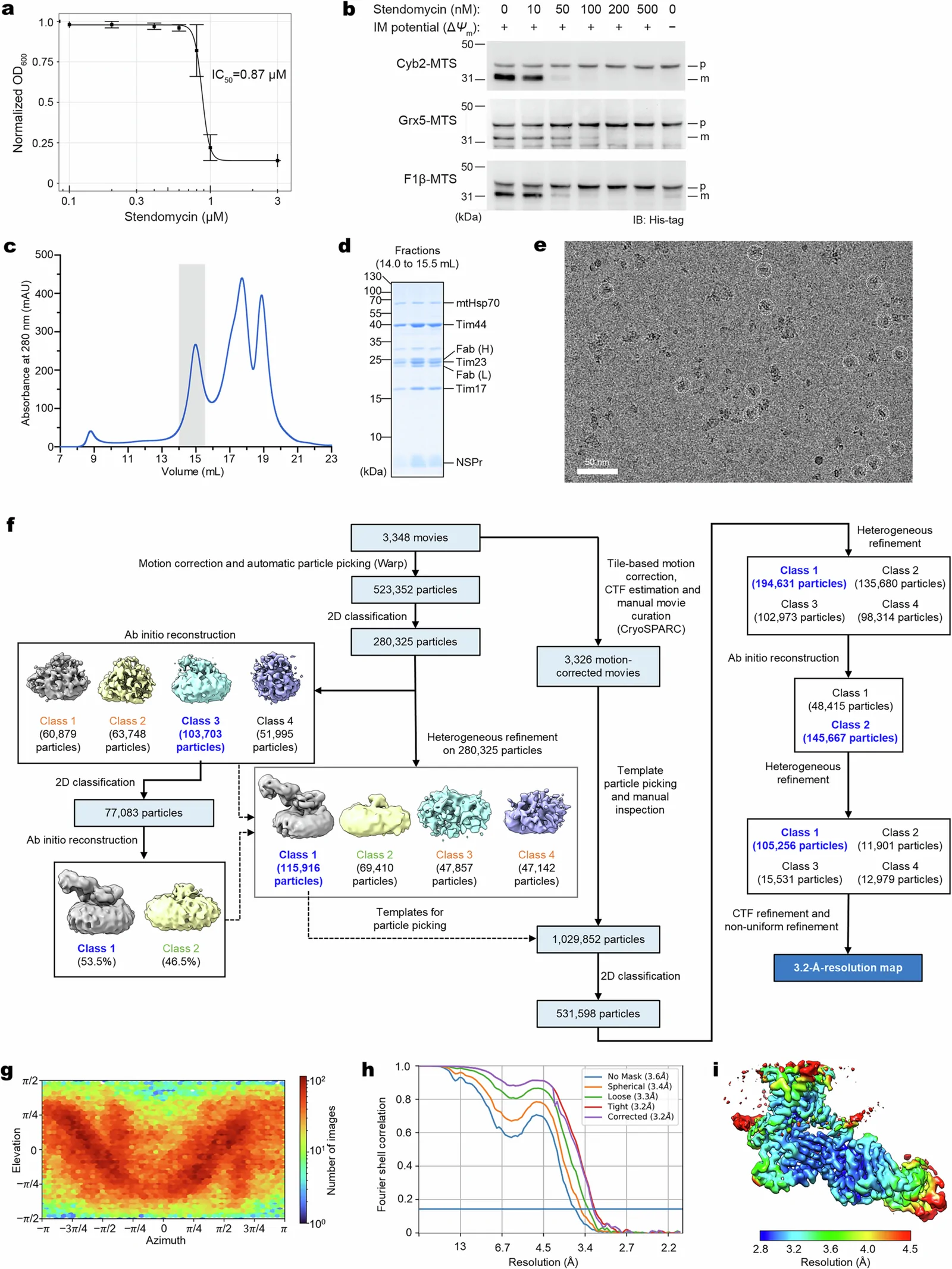
Cryo-EM analysis of the stendomycin-bound core TIM23 complex. (**a**) Growth inhibition of WT yeast (W303) by stendomycin. Cells were cultured in YPD medium. Data represent the means ± s.e.m. of three independent experiments. The curve was fitted using a dose-response model (see Methods). (**b**) In-vitro mitochondrial import assay of model client proteins. Where indicated, mitochondrial membrane potential was dissipated with valinomycin (−Δ*Ψ*_m_). p, precursor form; m, mature imported form. Shown is a representative of three independent experiments. (**c**, **d**) Superose-6 size-exclusion chromatography profile of the core TIM23 complex reconstituted into peptidiscs. Fractions highlighted by a gray box were used for SDS-PAGE (panel **d**) and cryo-EM analysis. NSPr, peptidisc peptide. The purification experiment was repeated twice with similar results. (**e**) Representative cryo-EM image of the stendomycin-bound TIM23 complex (out of 3,348 images). TIM23 particles are indicated by white dotted circles. (**f**) Overview of the cryo-EM image process workflow. (**g**) Distribution of cryo-EM particle view orientations. (**h**) Fourier-shell correlation (FSC) curves of the final density map. (**i**) Local resolution distribution of the final density map. Source data

The recent core TIM23 cryo-EM structure revealed that protein translocation is mediated by the lipid-exposed concave cavity of Tim17 (ref. ^20^). Despite this advance, however, the mechanistic understanding of how Tim17 engages its substrate remains limited. Stendomycin, a lipodepsipeptide from *Streptomyces hygroscopicus* (Fig. 1a), is known to inhibit TIM23-driven protein import in *Saccharomyces cerevisiae* and mammalian cells^32–34^. To better understand the mechanism of TIM23, we first investigated stendomycin-mediated inhibition using cryo-EM. As reported previously^32^, stendomycin showed a moderate (~0.9 µM) half-maximal inhibitory concentration (IC_50_) in our yeast growth assay (Extended Data Fig. 1a). In yeast, the presence of a thick cell wall and efficient efflux pumps may reduce the effective cellular concentration of added stendomycin. Therefore, to more accurately measure the stendomycin–TIM23 interaction strength, we also tested import of dihydrofolate reductase (DHFR)-fused model substrates with three different MTSs into purified yeast mitochondria (Fig. 1b and Extended Data Fig. 1b). Stendomycin efficiently inhibited import of these substrates at an IC_50_ of ~20–40 nM, validating a strong interaction with TIM23 required for structural analysis.

**Extended Data Fig. 2:**
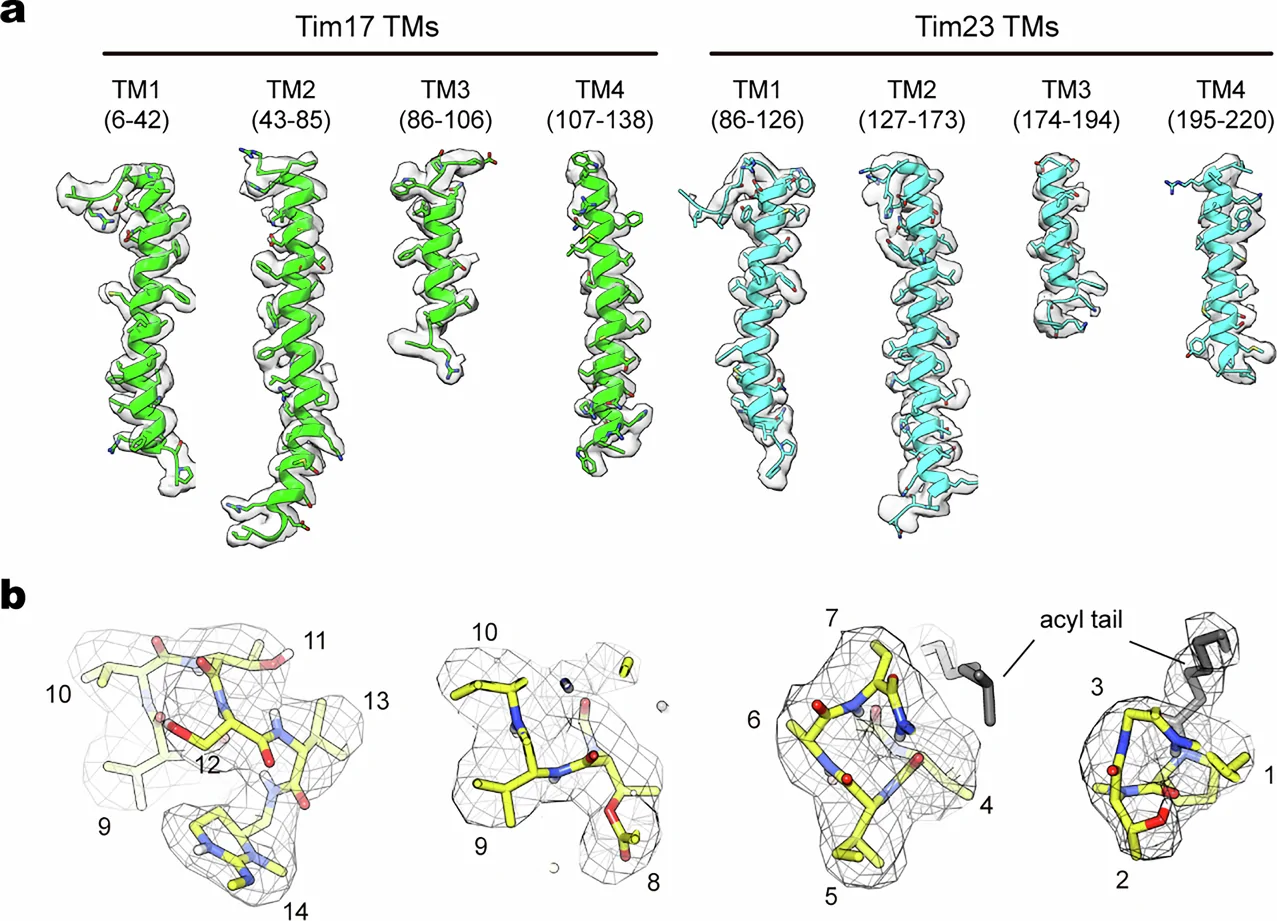
Cryo-EM density map quality and atomic model. (**a**) Segmented cryo-EM densities (gray semitransparent surfaces) overlaid with the atomic model (ribbons and sticks) for the Tim17 and Tim23 TMs. Parenthetical ranges indicate the displayed amino acid positions. (**b**) As in **a**, showing the density (mesh) and atomic model (sticks) for stendomycin. Panels progress left to right from the IMS-proximal face to the matrix-proximal face, illustrating individual residues (numbered according to Fig. 1a).

To enable cryo-EM analysis, we first purified the heterotrimeric core TIM23 complex from yeast, consisting of Tim17, Tim23 and Tim44, as performed previously^20^. Since stendomycin is a largely hydrophobic molecule, its binding could be weakened by free detergent micelles in the sample. To address this, we reconstituted TIM23 into peptidiscs^35^ before adding stendomycin (Extended Data Fig. 1c,d). With the aid of a monoclonal antibody fragment (Fab) specific to Tim44 as a fiducial marker, we obtained a cryo-EM map of the stendomycin-bound TIM23 complex at an overall resolution of 3.2 Å (Fig. 1c–e, Extended Data Figs. 1e–i and 2, and Supplementary Table 1). Most regions of the complex were well resolved in the map. Notably, a well-defined density of stendomycin was clearly visible in the Tim17 cavity with a local resolution of ~3.0–3.4 Å, allowing us to build a reliable atomic model.

### Structural basis for stendomycin-mediated TIM23 inhibition

**Fig. 2:**
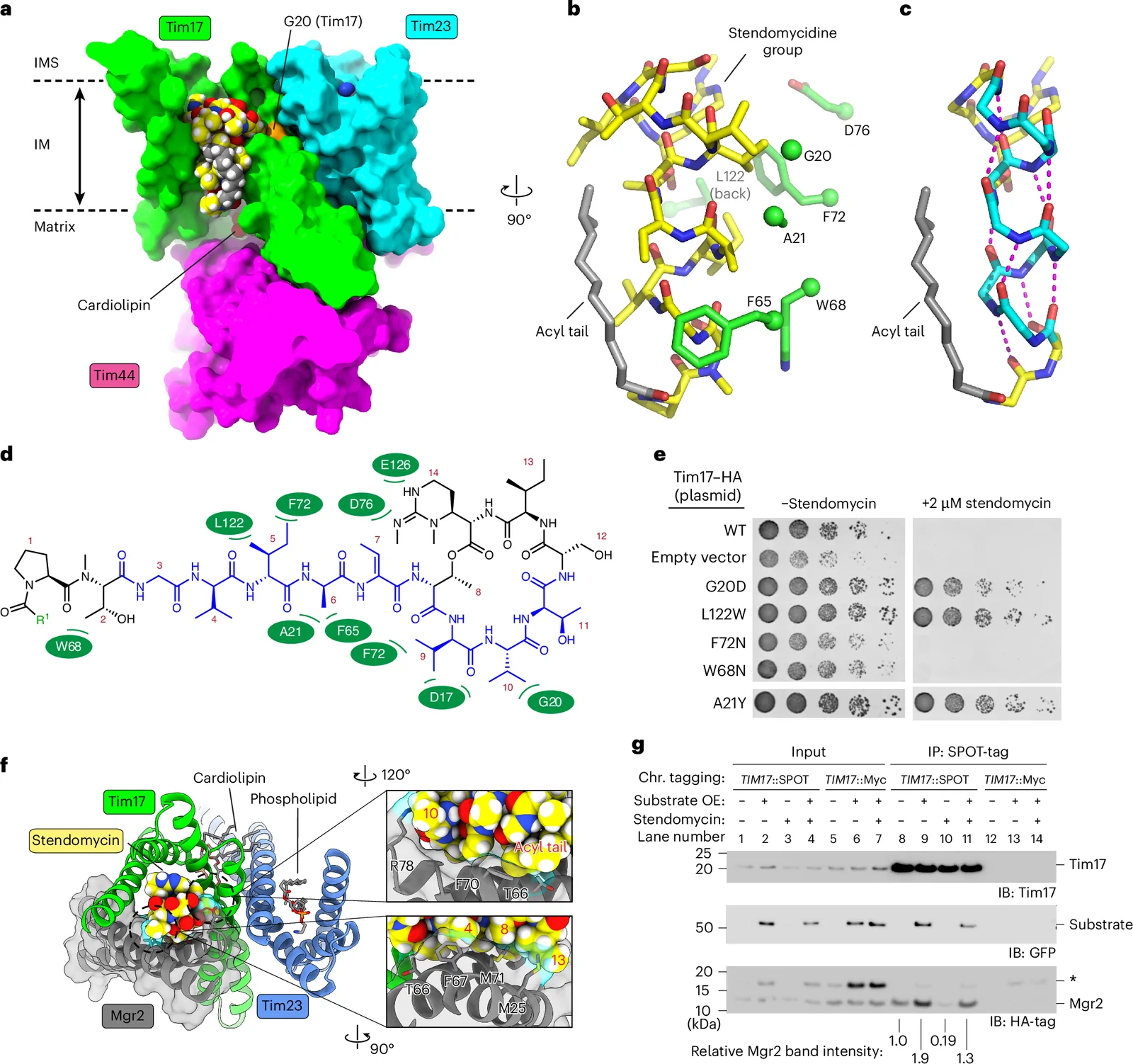
Interaction between stendomycin and Tim17. **a**, Side view of the stendomycin-bound TIM23 complex, showing the protein in surface representation and stendomycin in space-filling representation, highlighting the extensive interaction between stendomycin and the Tim17 cavity. Known stendomycin-resistant mutations occur at Gly20, Ala21 and Leu122. The position of Gly20 is indicated in orange. Ala21 and Leu122 are located behind the stendomycin in this view and thus are not visible. **b**, Close-up view of stendomycin and surrounding Tim17 side chains, shown in stick representation. The view is rotated 90° relative to **a** (with Tim17 cavity positioned on the right side). **c**, As in **b**, but displaying only the backbone structure and acyl tail of stendomycin. Hydrogen bonds within the left-handed α-helix are indicated by magenta dashed lines. **d**, Map of Tim17 residues interacting with stendomycin. The portion of stendomycin forming the left-handed α-helix is shown in blue. **e**, Yeast growth assay showing stendomycin resistance conferred by G20D, L122W and A21Y (ref. ^32^ and this study). We note that all mutants support yeast viability in place of WT Tim17 (Extended Data Fig. 3g). HA, hemagglutinin. **f**, Steric clashes between stendomycin and Mgr2. Mgr2 (gray) was positioned into the stendomycin-bound TIM23 based on the AlphaFold2-predicted Tim17-Mgr2 heterodimer (left, view from IMS). Stendomycin is depicted as spheres. Regions of major steric clashes (dashed circles) are shown in magnified insets. Cyan surfaces indicate clashing volumes. Numbers in red denote residue positions in stendomycin. **g**, IP of SPOT-tagged Tim17 from isolated mitochondria (Tim17-Myc was used as a negative control). Where indicated, mitochondria were prepared from cells overexpressing a stall-generating substrate (Grx5-S80-sfGFP; ref. ^20^) and/or treated with 3 µM stendomycin before SPOT-IP. Asterisk denotes the unprocessed form of Mgr2 (ref. ^58^). The experiments were repeated twice with similar results. Chr., chromosomal; IB, immunoblot. Source data

**Extended Data Fig. 3:**
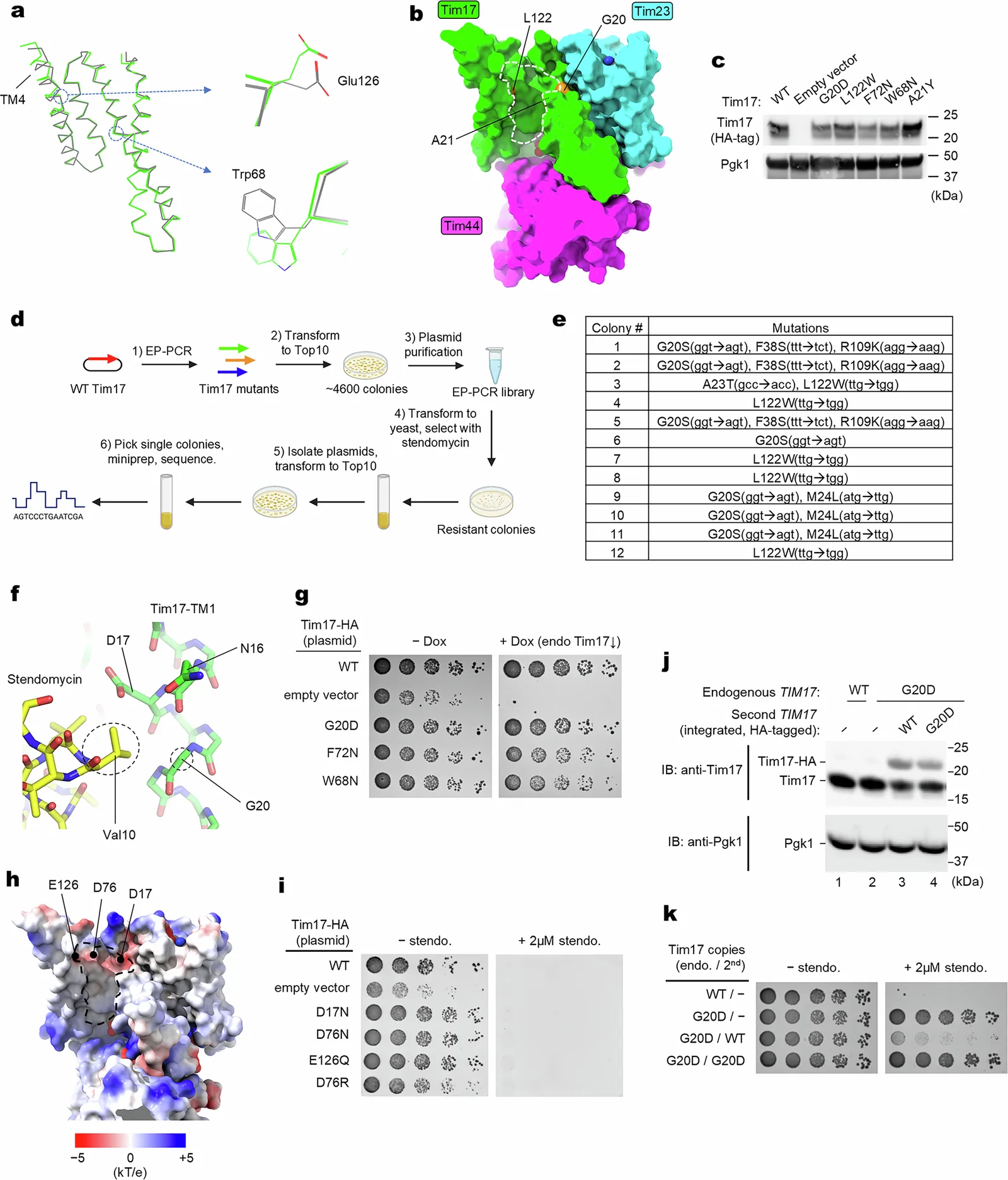
Mechanisms of stendomycin binding to TIM23 and resistance. (**a**) Structural overlay of Tim17 from the apo state (gray; PDB: 8SCX) and the stendomycin-bound structure (green). The root mean square deviation over 115 aligned Tim17 Cα atoms is 0.45 Å. Minor changes in the position of TM4 and sidechain rotamers of E126 and W68 are highlighted. (**b**) Positions of known stendomycin-resistance-conferring mutations in Tim17 (highlighted in orange). The structure of the core TIM23 complex is shown in surface representation. Stendomycin binding surface is outlined by white dashed line. (**c**) Expression of the indicated Tim17 mutants. Among the five tested mutants, G20D, L122W, and A21Y confer resistance to stendomycin (see Fig. 2e). Pgk1, loading control. The experiment was performed once. (**d**) Overview of error-prone (EP) PCR approach to identify stendomycin resistant mutations in Tim17. (**e**) Summary of DNA sequencing results for stendomycin-resistant Tim17 mutants (twelve randomly selected colonies). (**f**) Close-up view of the G20 position of Tim17. The Val10 side chain of stendomycin points toward a pocket surrounded by Tim17 residues, N16, D17, and G20. (**g**) Functionality of the indicated Tim17 mutants were confirmed using the strain yYC17b, where endogenous Tim17 expression is suppressed by doxycycline. (**h**) As in **b**, but showing the surface electrostatic potential. The positions of the three conserved acidic residues are indicated. Black dashed line, stendomycin-binding surface. (**i**) Mutations of conserved acidic residues do not confer stendomycin resistance. Yeast strain yYC17b was transformed with plasmids encoding the indicated Tim17 variants, and the serial dilutions of the resulting cells were spotted onto YPD agar plates with and without stendomycin. (**j**) Levels of Tim17 in yeast strains expressing WT Tim17 alone (lane 1), G20D Tim17 alone (lanes 2 and 4), and both WT and G20D (lane 3) were analyzed by immunoblotting (IB) using anti-Tim17 antibodies. Pgk1, loading control. The experiments were repeated twice with similar results. (**k**) Stendomycin sensitivity of strains used in panel **j** was tested on YPD agar medium in the absence or presence of stendomycin. Source data

Stendomycin binds to the concave cavity of Tim17, adopting a cork stopper-like structure (Fig. 2a–d). Since this cavity forms the polypeptide translocation path^20,25,26^, stendomycin may function as a molecular plug that directly competes with import substrates, explaining its inhibitory effects on mitochondrial protein import. Stendomycin binding does not induce major conformational changes in TIM23, as the structure is nearly identical to that of the idle state with the exceptions of a minor tilting of Tim17-TM4 and altered side-chain rotamers for Trp68 and Glu126 in the Tim17 cavity to accommodate stendomycin (Extended Data Fig. 3a). The binding is mediated largely by van der Waals interactions between the hydrophobic surfaces of stendomycin and the Tim17 cavity. Overall, stendomycin is ideally shaped to maximize contacts with the concave surface of the Tim17 cavity.

Previously, an L122W mutation in yeast Tim17 was shown to confer stendomycin resistance^32^. Our structure predicts that this mutation will cause steric clashes between the introduced tryptophan side chain and stendomycin (Extended Data Fig. 3b). Similarly, introducing a bulky amino acid at position 21 (A21Y), which would cause steric hindrance, also conferred resistance to stendomycin (Fig. 2e and Extended Data Fig. 3c). To identify additional resistance mechanisms, we generated a random mutant library of Tim17 using error-prone PCR and selected for resistant variants on stendomycin-containing medium (Extended Data Fig. 3d,e). All selected clones consistently included either L122W or G20S, suggesting that mutations in these two positions are most effective in resistance development. Unlike L122W, G20S does not seem to cause a major steric clash. However, near Gly20, the 10th residue (valine) of stendomycin sits in a relatively hydrophilic environment formed by Asn16 and Asp17 of Tim17 (Extended Data Fig. 3f). Thus, increased hydrophilicity by G20S may render stendomycin binding unfavorable in addition to producing minor steric effects. A G20D substitution, which was previously identified alongside L122W as a stendomycin-resistant variant^32^ (Fig. 2e), likely exerts a similar effect but with a greater steric clash.

At the center of the Tim17 cavity, two aromatic amino acids (Phe72 and Trp68) interface with stendomycin (Fig. 2b). Mutation of these to a smaller polar residue (F72N or W68N) did not confer resistance, suggesting that some reduction in hydrophobic contacts can be tolerated (Fig. 2e and Extended Data Fig. 3g). Stendomycin may form polar contacts between its stendomycidine residue and acidic amino acids (Asp76 and Glu126) in Tim17 (Fig. 2b and Extended Data Fig. 3h). However, mutating Asp76 or Glu126, including a charge reversal (D76R), did not result in stendomycin resistance, suggesting that polar interactions do not make major contributions to the binding (Extended Data Fig. 3i).

While stendomycin might exert cytotoxicity simply by blocking TIM23-dependent import, it remained possible that the effects were generated through an indirect, but Tim17 binding-dependent, dominant mechanism. To test this, we constructed a strain in which the endogenous *TIM17* locus harbors the stendomycin-resistant G20D mutation and introduced a second *TIM17* allele (wild-type (WT) or G20D) (Extended Data Fig. 3j,k). If cytotoxicity were solely due to import inhibition, the strain coexpressing WT and G20D Tim17 would retain stendomycin resistance conferred by the G20D variant. Conversely, a dominant effect would manifest as stendomycin sensitivity through binding to coexpressed WT Tim17. Growth assays showed that WT/G20D coexpressing cells indeed exhibited pronounced stendomycin-induced growth retardation compared with strains expressing G20D Tim17 alone, indicating the presence of a dominant component (below).

### Stendomycin mimics α-helical topogenic sequences

Stendomycin has a lasso-like chemical structure, consisting of a stem-like seven-residue linear segment and a seven-residue cyclic peptide ring (Fig. 1a). The tip of the stem is conjugated to an acyl chain, which faces the lipid phase in a hook-like conformation (Fig. 2a,b). The acyl chain likely reduces the interfacial energy between the peptide portion of stendomycin and membrane lipids and enhances its lipophilicity. Notably, parts of the stem and the cyclic peptide together form a left-handed α-helix (Fig. 2b,c). This region predominantly consists of D-amino acids, which accounts for the left-handedness of the helix.

The α-helical conformation of stendomycin has important implications for Tim17-substrate interactions. During initial engagement, the MTS is expected to bind within the Tim17 cavity. As MTSs are generally thought to form a helical conformation, the stendomycin-bound structure may thus mimic an MTS-engaged state (‘Discussion’).

While our structure revealed that the Tim17 cavity has an ideal shape for accommodating a short helix, stendomycin would interfere with Mgr2 binding. Structural superposition of Mgr2 using an AlphaFold model^36,37^ indicates clear steric clashes with stendomycin (Fig. 2f). To experimentally validate this observation, we treated isolated mitochondria with stendomycin and immunoprecipitated Tim17 to assess the copurification of Mgr2 (Fig. 2g). The results show that stendomycin substantially reduces the coimmunoprecipitation (co-IP) of Mgr2 (lanes 8 versus 10). The residual Mgr2 binding may stem from endogenous client proteins trapped in translocation intermediates, strengthening the Mgr2–Tim17 association^20^. In fact, increased Mgr2 co-IP efficiency (lanes 8 versus 9) and greater stendomycin-resistant Mgr2 binding were observed when a translocation-stalling model substrate was overexpressed before mitochondrial isolation (lanes 10 versus 11). These results also agree with the proposed model that Mgr2 dynamically associates with Tim17 in a translocation-dependent manner^20^.

### Stendomycin selectivity of the Tim17 and Tim22 cavities

**Fig. 3:**
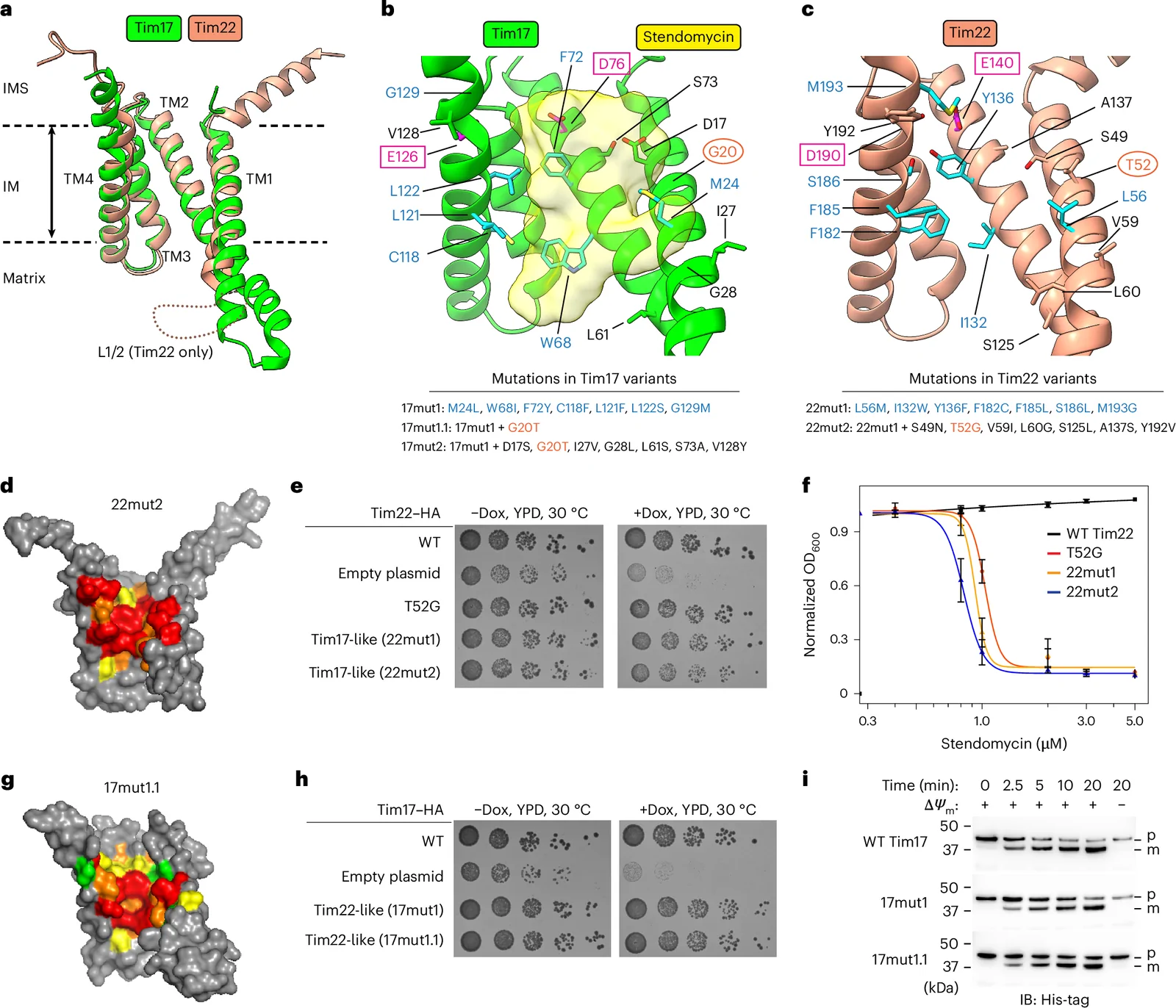
Stendomycin selectivity and interchangeability of the Tim17 and Tim22 cavities. **a**, Structural overlay of Tim17 (PDB 8SCX) and Tim22 (PDB 6LO8) showing the homologous backbone structures. **b**,**c**, Comparison of cavity-lining amino acids in Tim17 (**b**) and Tim22 (**c**). Residues highlighted with magenta boxes indicate acidic amino acids conserved across Tim17 and Tim22 proteins. Residues highlighted with orange ovals indicate equivalent but non-conserved Gly20 and Thr52 that are important for stendomycin sensitivity. Residues labeled in blue denote positions mutated in the mut1 versions of Tim17–Tim22 swapping mutants. In mut2 mutants, seven additional residues were substituted, including black-labeled positions and D17S/G20T (for 17mut2) or S49N/T52G (for 22mut2). The substitutions are also listed below the structure figures. **d**, Surface representation of Tim22 showing the locations of mutated residues in 22mut2. Red, mutated residues; orange, residues identical between Tim17 and Tim22; yellow, residues that are not included in mutation due to high similarity with Tim17 counterparts (A21:G53, D76:E140, A91:G155, S114:G178 and E126:D190 in Tim17:Tim22 pairs); and green, unchanged residues. Also Extended Data Fig. 4a. **e**, Yeast growth complementation assay with WT and mutant Tim22. Endogenous Tim22 expression was repressed by doxycycline (Dox). Shown are results from YPD medium at 30 °C. For additional conditions, see Extended Data Fig. 4c. **f**, Stendomycin dose–response curves derived from yeast growth assays. All strains carry the G20D mutation in the endogenous *TIM17* gene. IC_50_ values are 1.02 µM for T52G, 0.93 µM for 22mut1 and 0.85 µM for 22mut2. Data represent means ± s.e.m. of three independent experiments. **g**, As in **d**, but showing the Tim17 structure and the positions of mutated residues in 17mut1.1. **h**, As in **e**, but testing the functionality of 17mut1 and 17mut1.1. For additional conditions, see Extended Data Fig. 4k. **i**, Kinetics of in vitro mitochondrial import of Cyb2Δ-DHFR. Shown is a representative of three independent experiments. p, precursor form; m, mature imported form. Source data

Both Tim17 and Tim22 are essential for cell viability in yeast. The fact that a single amino acid mutation on Tim17 can confer stendomycin resistance indicates that stendomycin does not bind to Tim22, despite its structural similarity to Tim17 (Fig. 3a). We reasoned that understanding the cavity differences that dictate stendomycin selectivity could allow us to engineer stendomycin binding into Tim22.

**Extended Data Fig. 4:**
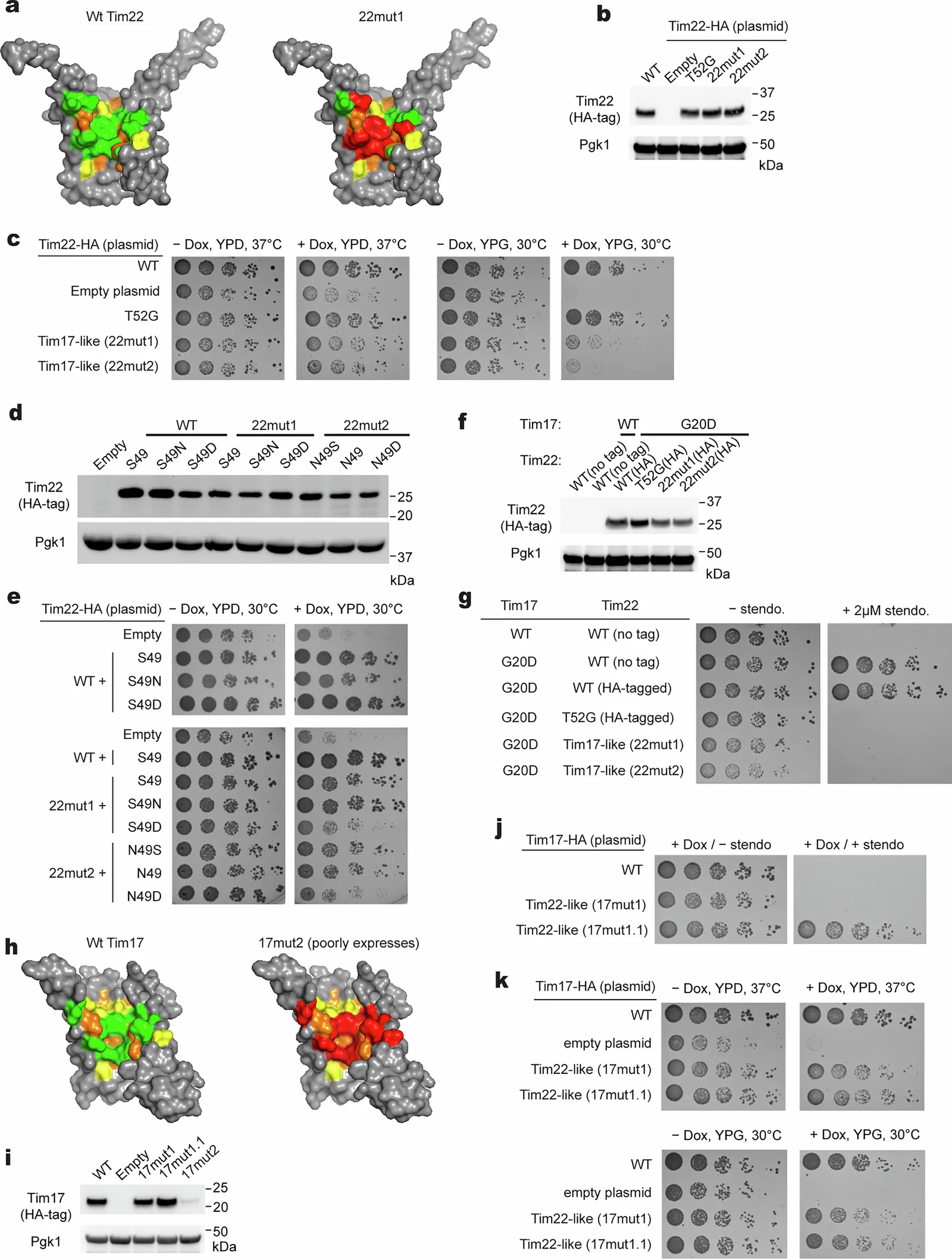
Cavity-swapping experiments. (**a**) As in Fig. 3d, but showing the maps for WT Tim22 and the 22mut1 variant. For the color code, see the Fig. 3 legend. (**b**) Immunoblot analysis verifying expression of the indicated Tim22 mutants in whole-cell lysates. Yeast strain yYC22 was transformed with plasmids (pYC22, empty pYTK-e115, or pYC109 to 111) encoding the indicated Tim22 variants. Pgk1, loading control. (**c**) Growth complementation assay of Tim22 variants. Yeast strain yYC22, where endogenous Tim22 expression is repressed by doxycycline (Dox), was transformed with plasmids (pYC22, empty pYTK-e115, or pYC109 to 111) expressing the indicated variants. Serial dilutions of cells were spotted on YPD or YPG agar plates and incubated at 37 °C or 30 °C. (**d,e**) As in **b** and **c**, but testing expression and growth complementation of the indicated Tim22 mutants carrying charge-modifying substitutions at position 49. (**f**) Immunoblot analysis verifying expression of the indicated Tim22 mutants in strains carrying the chromosomal copies of *TIM17* and *TIM22* replaced with the G20D mutation and the indicated modifications. (**g**) Strains from panel **f** were spotted on YPD agar plates with and without stendomycin to assess stendomycin sensitivity. (**h**) As in Fig. 3g, but showing the maps for WT Tim17 and the 17mut2 variant. For the color code, see the Fig. 3 legend. (**i**) As in **b**, but testing expression of the indicated Tim17 variants. (**j**) Test for stendomycin sensitivity of Tim17 variants. Note that 17mut1.1 contains an additional G20T mutation compared to 17mut1. (**k**) Growth complementation assay testing the functionality of 17mut1 and 17mut1.1 in yeast cells. Strain yYC17b was transformed with Tim17-expressing plasmids (pYC17a, empty pYTK-e115, pYC106 and pYC108). Endogenous Tim17 expression was repressed by doxycycline. Serial dilutions of cells were spotted on YPD or YPG agar plates and incubated at 37 °C or 30 °C. Experiments in **b**, **d**, **f**, and **i** were repeated twice with similar results. Source data

To test this idea, we compared the cryo-EM structures of Tim17 and Tim22 to survey differences in their cavities (Fig. 3b,c). Using this information, we designed three Tim22 variants: (1) a single amino acid substitution mutant T52G, (2) a combinatorial mutant (22mut1), in which seven residues in the Tim22 cavity were replaced with their stendomycin-contacting counterparts from Tim17, and (3) a further combinatorial mutant (22mut2), where seven additional mutations, including T52G, were incorporated into 22mut1 to more closely mimic the Tim17 cavity (Fig. 3d and Extended Data Fig. 4a). The T52G mutant was chosen because Thr52 in Tim22 corresponds to Gly20 in Tim17. As G20D and G20S mutations in Tim17 confer substantial stendomycin resistance (ref. ^32^ and this study), the inability of stendomycin to bind Tim22 could be due to the presence of a threonine at this position.

We first evaluated the functionality of the three Tim22 mutants using a yeast growth assay. All mutants were confirmed to be expressed and retain overall functionality (Fig. 3e and Extended Data Fig. 4b). Cells expressing 22mut2 grew similarly to the WT on glucose (yeast extract peptone dextrose (YPD)) medium, despite 14 residues in the cavity being substituted. However, both 22mut1 and 22mut2, but not T52G, displayed substantial defects on nonfermentable glycerol (yeast extract peptone glycerol (YPG)) medium, where mitochondrial function is more critical (Extended Data Fig. 4c). Taken together, replacing Tim22’s cavity with that of Tim17 retains the overall functionality of Tim22 despite suboptimal performance.

In our initial experiments, both 22mut1 and 22mut2 carried a neutral amino acid at position 49 (Ser and Asn, respectively), as in WT Tim22. However, the equivalent residue in Tim17 is Asp (D17), a negatively charged amino acid that is well conserved across Tim17 proteins. We therefore also examined the effect of a negative charge at this position (Extended Data Fig. 4d,e). Although an S49D mutation in the WT Tim22 background fully supported yeast growth, the same mutation caused a noticeable growth defect in 22mut1 and 22mut2. 22mut2/N49D exhibited a near-null growth phenotype, only marginally better than the empty plasmid control. Thus, introducing a negative charge at this position likely impairs Tim22 function modestly, and its combination with additional Tim17-mimicking mutations synergistically reduces activity.

Next, we tested the Tim22 variants for stendomycin sensitivity (Extended Data Fig. 4f,g). The variants were introduced into yeast to replace endogenous Tim22. These strains also carried the G20D mutation in *TIM17*, which abolishes stendomycin binding to Tim17, thereby isolating Tim22-specific effects in growth assays. On agar medium, all three Tim22 variants displayed clear sensitivity to stendomycin. Even the single T52G mutation enabled stendomycin binding to Tim22, despite the substantial surface differences between Tim22 and Tim17 cavities. While 22mut1 retains Thr52, this variant was also sensitive to stendomycin, suggesting that stendomycin binding is affected not only by the Thr52 (Gly20 in Tim17) position but also by other amino acids in the cavity.

To quantitatively evaluate the stendomycin-binding affinities among the Tim22 mutants, we measured IC_50_ values for growth inhibition in liquid cultures (Fig. 3f). The T52G mutant showed an IC_50_ of ~1.0 µM, whereas 22mut1 and 22mut2 exhibited IC_50_ values of 0.93 µM and 0.85 µM, respectively. Notably, the IC_50_ value for 22mut2 closely matches that of Tim17. Thus, the introduced Tim17-mimicking cavity mutations enhance stendomycin binding and make the cavity of Tim22 structurally more similar to that of Tim17.

### Tim17 and Tim22 cavities are functionally interchangeable

The observation that the Tim22 variants 22mut1 and 22mut2 are largely functional in the growth assay was unexpected. This prompted us to hypothesize that mutating the cavity of Tim17 to mimic that of Tim22 might also preserve the Tim17 function. We designed two Tim22-mimicking Tim17 variants, 17mut1 and 17mut2, equivalent to 22mut1 and 22mut2 (Fig. 3g and Extended Data Fig. 4h,i). We also generated a minor variant of 17mut1 (denoted 17mut1.1), which also includes a G20T mutation. While 17mut2 exhibited poor expression, 17mut1 and 17mut1.1 expressed well in yeast.

In growth complementation assays, where endogenous *TIM17* is repressed, 17mut1 and 17mut1.1 rescued cell growth as effectively as WT Tim17 (Fig. 3h). In stendomycin-dependent growth inhibition assays, 17mut1 was sensitive to stendomycin, whereas 17mut1.1 was resistant, indicating that the Tim22-mimicking substitutions in Tim17 do not interfere with stendomycin binding, but the additional G20T mutation abolishes it (Extended Data Fig. 4j).

These findings suggest that despite the functional divergence of the TIM23 and TIM22 complexes over the course of evolution, the cavities of Tim17 and Tim22 share a common core function. However, as with the Tim22 variants, there could be nuance in the activity of the Tim17 variants. Therefore, we measured the kinetics of Cyb2Δ-DHFR import in vitro using mitochondria expressing WT Tim17, 17mut1 or 17mut1.1 (Fig. 3i). The results indicated no major differences between the WT and variant Tim17. Similarly, we did not observe any notable growth defects with the variants at an elevated temperature (37 °C) or on nonfermentable medium, unlike 22mut1 and 22mut2 (Extended Data Fig. 4k). Taken together, our data indicate that the Tim17 and Tim22 cavities are largely interchangeable while maintaining their respective functions. Conversely, this suggests that their client specificities are mainly dictated by other subunits of the complexes (‘Discussion’).

### Conserved negative charges in Tim17 can be relocated

**Fig. 4:**
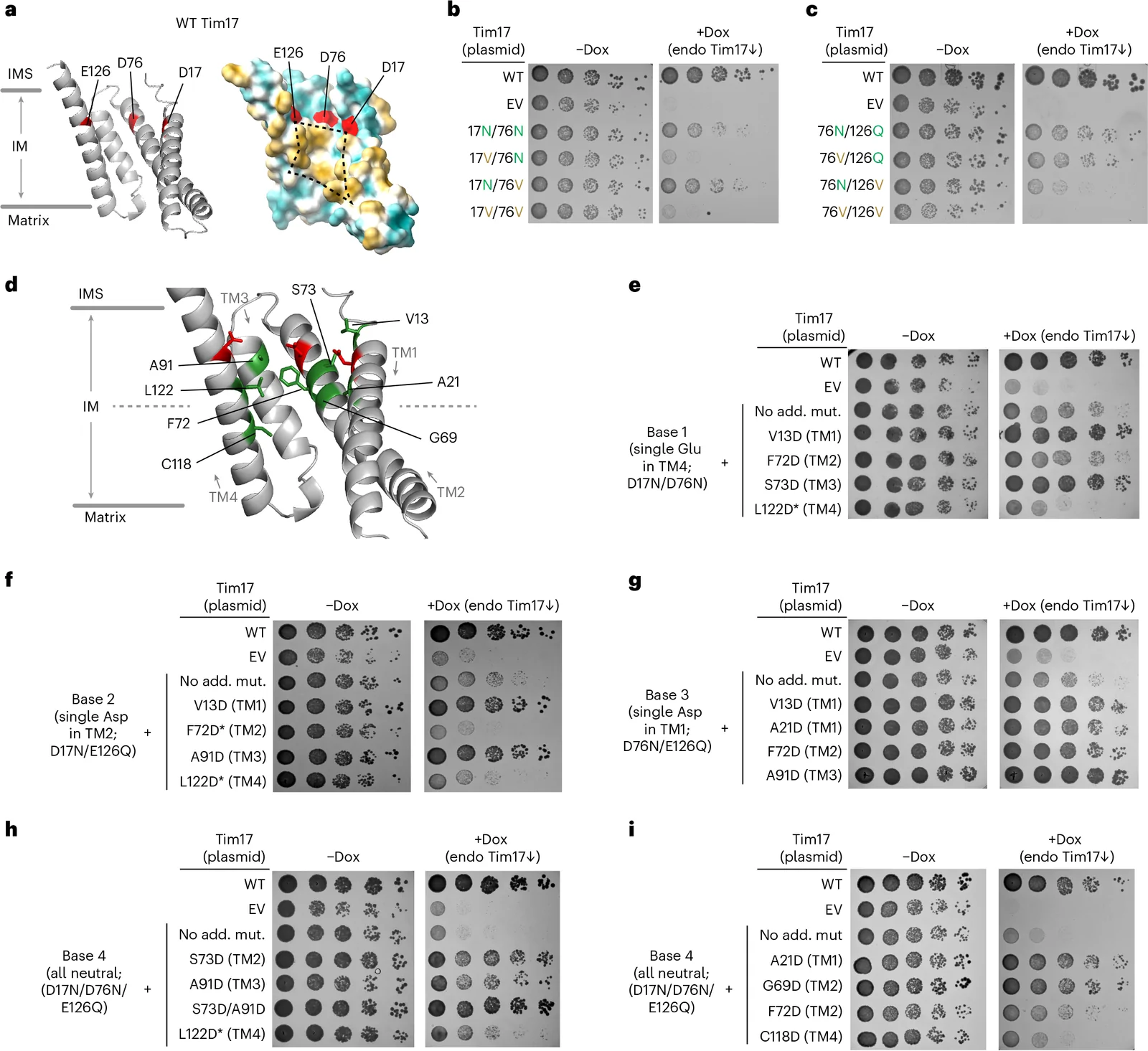
Positional flexibility of negative charges in the Tim17 cavity. **a**, Positions of conserved acidic amino acids in the Tim17 cavity. The left panel shows a ribbon diagram, and the right panel shows the surface representation colored by hydrophilicity (cyan, hydrophilic; tan, hydrophobic). Conserved acidic residues are highlighted in red. **b**,**c**, Yeast growth complementation assay using WT Tim17 and the indicated mutants (D17/D76 mutants in **b** and D76/E126 mutants in **c**). Endogenous Tim17 expression was repressed by Dox. **d**, Structural diagram of Tim17 indicating the positions (highlighted in green) where negatively charged residues were reintroduced into charge-neutralizing mutant backgrounds (base 1, D17N/D76N; base 2, D17N/E126Q; base 3, D76N/E126Q; and base 4, D17N/D76N/E126Q). The positions of D17, D76 and E126 residues are shown in red. **e**–**i**, As in **b** and **c**, but testing growth rescue by indicated charge-modified Tim17 mutants (base 1, base 2 and base 3 variants in **e**, **f** and **g**, respectively; base 4 variants split in **h** and **i**). Asterisk denotes mutants for which little or no expression was detected (Extended Data Fig. 5). Add. mut., additional mutations.

**Extended Data Fig. 5:**
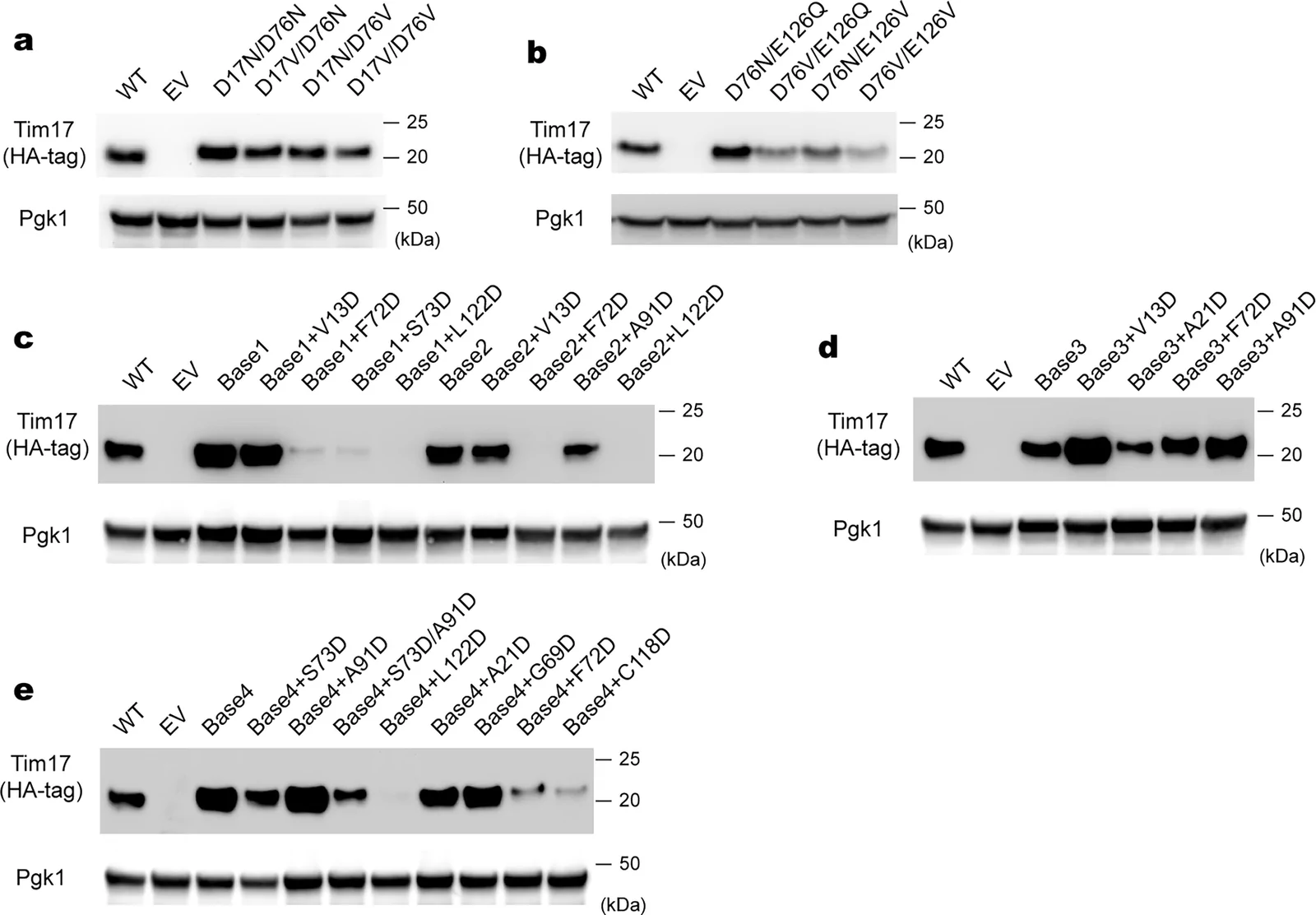
Expression test for Tim17 variants containing mutations in negatively charged residues. (**a,b**) Immunoblot analysis testing expression of Tim17 mutants in which negative charged residues were substituted with valine. Plasmid pYC17a expressing the indicated Tim17 variants was transformed into strain W303. Pgk1, loading control. (**c-e**) As in **a** and **b**, but testing expression of Tim17 variants with an aspartate residue(s) introduced at various positions within the cavity. Note that certain mutants failed to express (L122D in all backgrounds, and F72D in Base 2). The Base 1 + S73D mutant showed only weak expression, but still displayed functional rescue (see Fig. 4e). Experiments in **a**–**e** are repeated twice with similar results. Source data

The cavity of yeast Tim17 contains three highly conserved acidic residues, Asp17, Asp76 and Glu126, which are exposed to the IMS leaflet of the IM (Fig. 4a). Similarly, yeast Tim22 has two highly conserved acidic residues, Glu140 and Asp190, which correspond to Asp76 and Glu126 of Tim17, respectively^22^. Previous Tim17 studies showed that individual mutations of these glutamate or aspartate residues to neutral asparagine or glutamine result in no growth defects, while double mutations lead to substantial growth impairment, and a triple mutant is barely viable^20^. Similarly, mutations in Glu140 and Asp190 of Tim22 result in functional defects^22^. One reason for the functional importance of these residues would be their role in local membrane distortion (thinning) to reduce the energy barrier for the translocation or membrane insertion of client polypeptides^20,22^. Supporting this idea, replacing Asp17 or Glu126 of Tim17 with similarly sized but hydrophobic valine displayed much more severe growth defects compared with mutations to asparagine or glutamine (Fig. 4b,c and Extended Data Fig. 5a,b). Although D76V did not exacerbate the growth impairment compared with D76N, its combination with E126V was nearly inviable.

While the positions of these acidic amino acids are conserved in the cavity structure, we hypothesized that altering their positions may be tolerated for functionality if their main function is membrane thinning. We chose three partially defective Tim17 mutants, D17N/D76N, D17N/E126Q and D76N/E126Q (referred to as base 1, 2 and 3, respectively), each of which contains only a single acidic amino acid (E126, D76 and D17, respectively) in the cavity. Then, we incorporated one additional aspartate at different positions within the cavity for growth rescue (Fig. 4d–g and Extended Data Fig. 5c,d). The results show that aspartate at several alternative positions, such as V13D, A21D, S73D and A91D, can functionally substitute the native acidic amino acids. Mutants with two aspartate residues closely neighboring each other (for example, base 2 + A91D, base 3 + V13D and base 3 + A21D) also showed improved cell growth compared with their respective base mutants. Thus, increased negative charge density at a localized area can also support the Tim17 function well, provided that the variant is sufficiently expressed. D17N/D76N/E126Q (base 4) variants with a single aspartate residue introduced at various sites within the IMS side half of the cavity (A21, G69, S73 and A91) showed clear growth improvement (Fig. 4h,i and Extended Data Fig. 5e). Notably, the S73D/A91D mutant, where two aspartate residues are introduced at nonnative positions, supported cell growth as robustly as WT Tim17. These findings indicate that although acidic residues are essential for function, their exact placement within the cavity can vary without causing major functional disruption: an unexpected result considering their high evolutionary conservation.

### Physiological effects of stendomycin treatment

**Extended Data Fig. 6:**
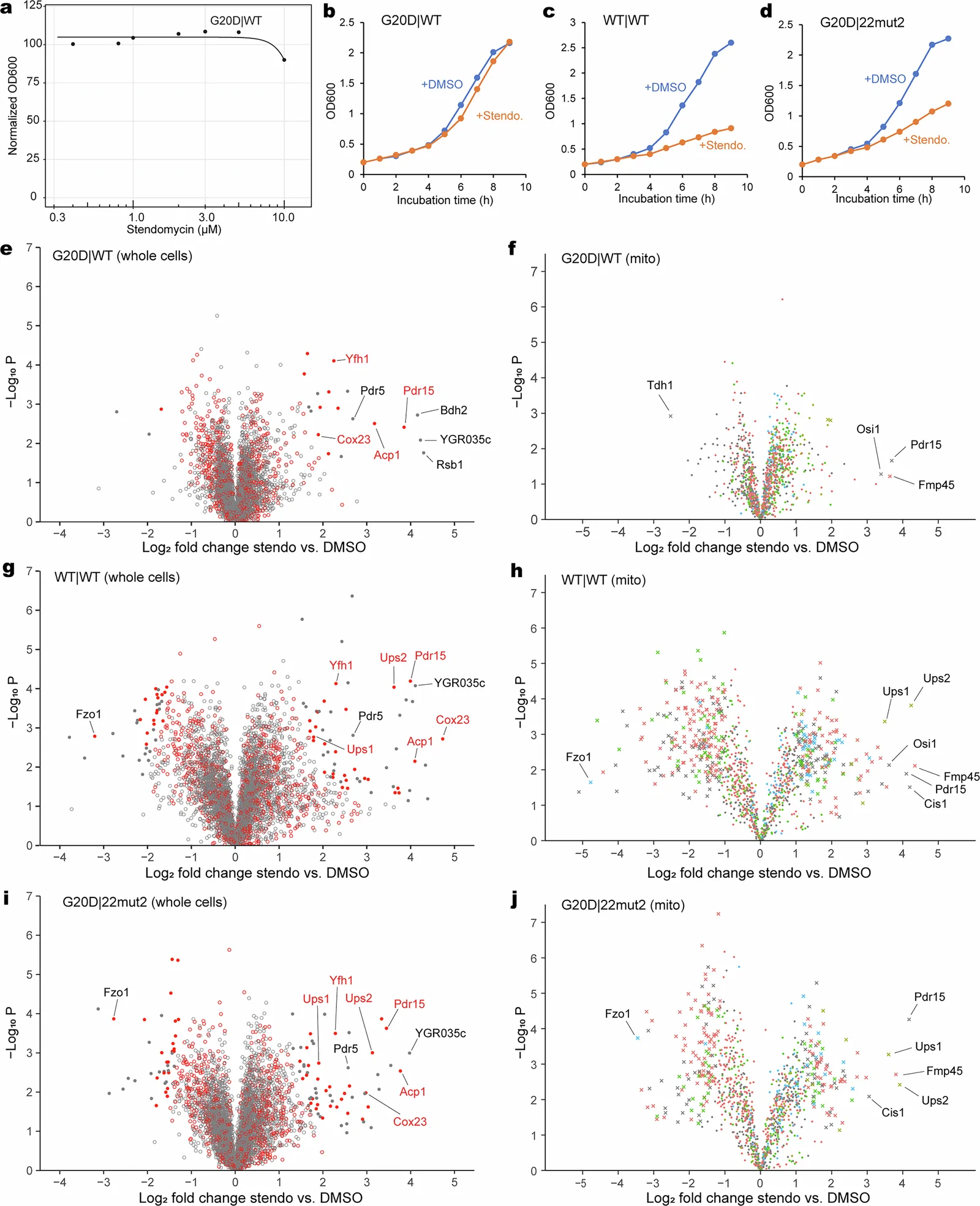
Quantitative mass spectrometry (MS) of the proteomic response to stendomycin treatment. (**a**) As in Fig. 3f, but showing an experiment with the stendomycin concentration up to 10 µM on the Tim17^G20D^|Tim22^WT^ strain (G20D|WT; strain yYC52). (**b**-**d**) Growth curves of stendomycin-resistant (G20D|WT) and stendomycin-sensitive (WT|WT and G20D|22mut2) strains. Stendomycin (3 µM) was added to the actively growing cultures in YPD medium at the zero time point. (**e**–**j**) Volcano plots showing the proteomic response of indicated strains to stendomycin treatment compared to DMSO control. Data were obtained from MS analysis of the whole-cell lysates (panels **e**, **g**, and **i**) and mitochondrial fractions (panels **f**, **h**, and **j**) from three independent experiments with *x*-axis values representing mean values. In panels **e**, **g**, and **i**, red and gray circles indicate mitochondrial and non-mitochondrial proteins, respectively; and closed and open circles represent statistically significant and non-significant changes, respectively, based on a two-sided Welch’s t-test with 1% FDR. In panels **f**, **h**, and **j**, only mitochondrial proteins are plotted with a color code on submitochondrial localization: blue, OM; olive, IMS; red-orange, IM; green, matrix; and gray, unknown or present in multiple compartments. Crosses (X), statistically significant changes; dots, non-significant changes, based on a two-sided Welch’s t-test with 1% FDR.

Next, we sought to investigate the cellular response on treatment with stendomycin. Defining the structural basis of stendomycin’s selectivity offered a unique opportunity to use stendomycin as a tool to specifically block Tim17 or Tim22. To this end, we analyzed the proteomic response of yeast cells treated with stendomycin using three selected strains: (1) WT|WT, yeast with WT Tim17 and WT Tim22, (2) G20D|WT, where the *TIM17* gene carries a stendomycin-resistant G20D mutation while Tim22 remains WT and (3) G20D|22mut2, where Tim22 includes mutations mimicking the stendomycin-sensitive cavity of Tim17. In growth assays, the G20D|WT strain exhibited strong stendomycin resistance with an IC_50_ of >10 µM (Extended Data Fig. 6a), whereas the WT|WT and G20D|22mut2 strains were sensitive (an IC_50_ of ~0.9 µM; Fig. 3f and Extended Data Fig. 1a) as expected for inhibition of the TIM23 and TIM22 complexes, respectively. In medium containing 3 µM stendomycin, the WT|WT and G20D|22mut2 strains grew linearly in contrast to the exponential growth observed in untreated cultures (Extended Data Fig. 6b–d). To gain insights into physiological changes, we conducted quantitative proteomics on both total-cell extracts and mitochondrial fractions after 5 hours of growth in YPD medium containing 3 µM stendomycin, quantifying 5,042 proteins (Extended Data Fig. 6e–j).

As expected, the samples from the stendomycin-resistant G20D|WT strain showed that a relatively small subset of proteins was significantly altered in abundance, most of which seem to be related to the general chemical stress response (Extended Data Fig. 6e,f). For example, among the highly upregulated proteins were Pdr5 and Pdr15, ABC transporters implicated in cellular detoxification^38,39^. Another example is the uncharacterized Cis1 paralog Ygr035c, which is known to be activated by the transcription factors Yrm1 and Yrr1, both of which are involved in multidrug resistance^40^. Similarly, expression of Bdh2 is known to be driven by drug-responsive transcription factors Pdr1 and Pdr3 (ref. ^41^). These proteins are commonly upregulated by stendomycin in all three strains. In addition, the mitochondrial proteins Acp1 and Yfh1 also exhibited increased abundance, although their roles in the stress response remain unclear.

By contrast, the stendomycin-sensitive WT|WT and G20D|22mut2 strains exhibited more pronounced proteomic perturbations (Extended Data Fig. 6g–j). Among upregulated proteins were the IMS proteins Ups1 and Ups2 (below), as well as the OM protein Cis1, a factor involved in the removal of import clients clogged in the TOM complex^42^. Conversely, Fzo1 levels decreased by approximately eightfold. Fzo1 (mitofusin), a mitochondrial fusion factor, was recently shown to rapidly degrade under mitochondrial stress, such as mitochondrial membrane potential (Δ*Ψ*_m_) dissipation^43^.

**Fig. 5:**
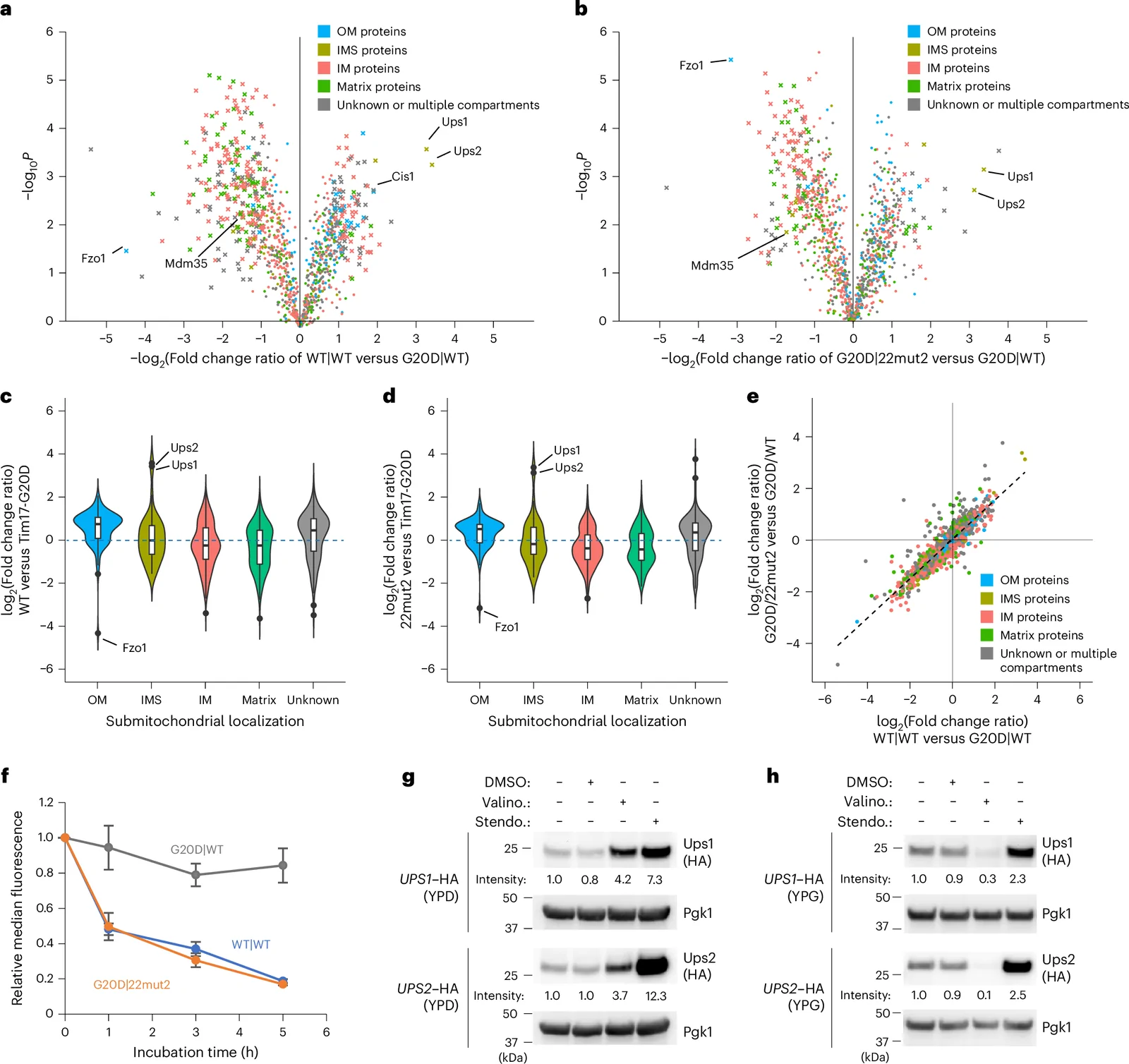
Stendomycin treatment induces mitochondrial membrane potential collapse and upregulation of Ups1 and Ups2. **a**,**b**, Volcano plot for the proteomic response to stendomycin treatment targeting Tim17 (**a**) or Tim22 (**b**). Panels **a** and **b** show protein abundance changes in the Tim17^WT^|Tim22^WT^ (WT|WT) strain and Tim17^G20D^|Tim22^mut2^ (G20D|22mut2), respectively, both relative to the Tim17^G20D^|Tim22^WT^ (G20D|WT) strain. Data points are color coded based on submitochondrial localization. The *x*-axis values represent mean values of three independent experiments. Crosses (x), statistically significant changes; dots, nonsignificant changes, based on a two-sided Welch’s *t*-test with 1% FDR. The data shown are from analysis of mitochondria-enriched fractions (for the whole-cell dataset, see Extended Data Fig. 6). **c**,**d**, Violin plots showing the distribution of fold changes in protein levels across mitochondrial subcompartments when Tim17 (**c**) or Tim22 (**d**) was targeted by stendomycin. White boxes and black bars indicate quartiles (25% and 75%) and medians, respectively. The data shown are from an analysis of mitochondrial-enriched fractions. **e**, Correlation plot showing individual mitochondrial proteins (dots) to compare protein abundance fold changes following Tim17 inhibition (*x* axis) and Tim22 inhibition (*y* axis). **f**, Mitochondrial membrane potential (Δ*Ψ*_m_) in intact yeast cells was measured by Rhodamine-123 staining and flow cytometry (also Extended Data Fig. 8a). Changes in Δ*Ψ*_m_ were monitored over time following addition of 3 µM stendomycin (means ± s.e.m. of three independent experiments). **g**,**h**, Immunoblotting analysis of cell lysates after treatment with 3 µM stendomycin (Stendo.), 45 µM valinomycin (Valino.) or DMSO (vehicle control) for 5 h. Endogenous Ups1 and Ups2 were C-terminally tagged with an HA epitope. Cells were cultured in YPD or YPG medium as indicated. Pgk1, loading control. The experiments were repeated twice with similar results. Source data

**Extended Data Fig. 7:**
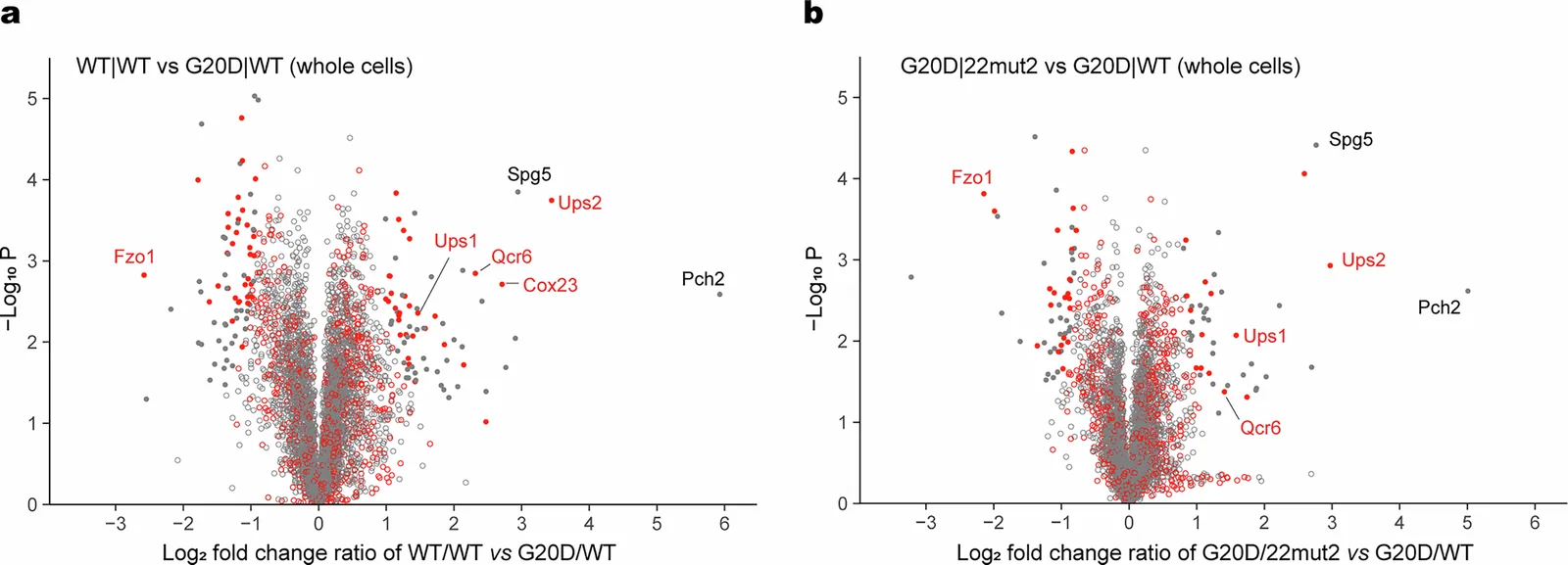
Whole-cell proteomic response of stendomycin-sensitive strains to stendomycin treatment compared to the stendomycin-resistant strain. (**a**,**b**) As in Fig. 5a and b, but showing the dataset from whole-cell lysate analysis (*x*-axis values represent mean values of three independent experiments). Red, mitochondrial proteins; gray, non-mitochondrial proteins. Closed circles, statistically significant changes; open circles, statistically non-significant changes, based on a two-sided Welch’s t-test with 5% FDR.

For additional proteomic comparisons, we derived ratios of stendomycin-induced fold changes: comparing WT|WT versus G20D|WT for Tim17 targeting, and G20D|22mut2 versus G20D|WT for Tim22 targeting (Fig. 5a,b and Extended Data Fig. 7). The mitochondrial fraction dataset revealed that out of 1,098 detected mitochondrial proteins, 383 and 134 were significantly (Welch’s *t*-test, 1% FDR) altered in these two sets, respectively. Examining changes across mitochondrial subcompartments, ~65% of IM and matrix proteins showed reduced abundance following stendomycin treatment (Fig. 5c). Although Tim22 primarily integrates multi-pass membrane proteins, targeting it with stendomycin also led to a decrease in the abundance of ~60% of matrix proteins (Fig. 5d). Another noteworthy observation is that ~70–75% of OM proteins increased in relative abundance, which suggests that their targeting remains generally unimpaired (Fig. 5c,d). Overall, abundance changes across submitochondrial compartments followed a similar pattern for Tim17 and Tim22 targeting. Similarly, per-protein comparisons also revealed a strong correlation in abundance changes between the two ratio sets (Fig. 5e).

**Extended Data Fig. 8:**
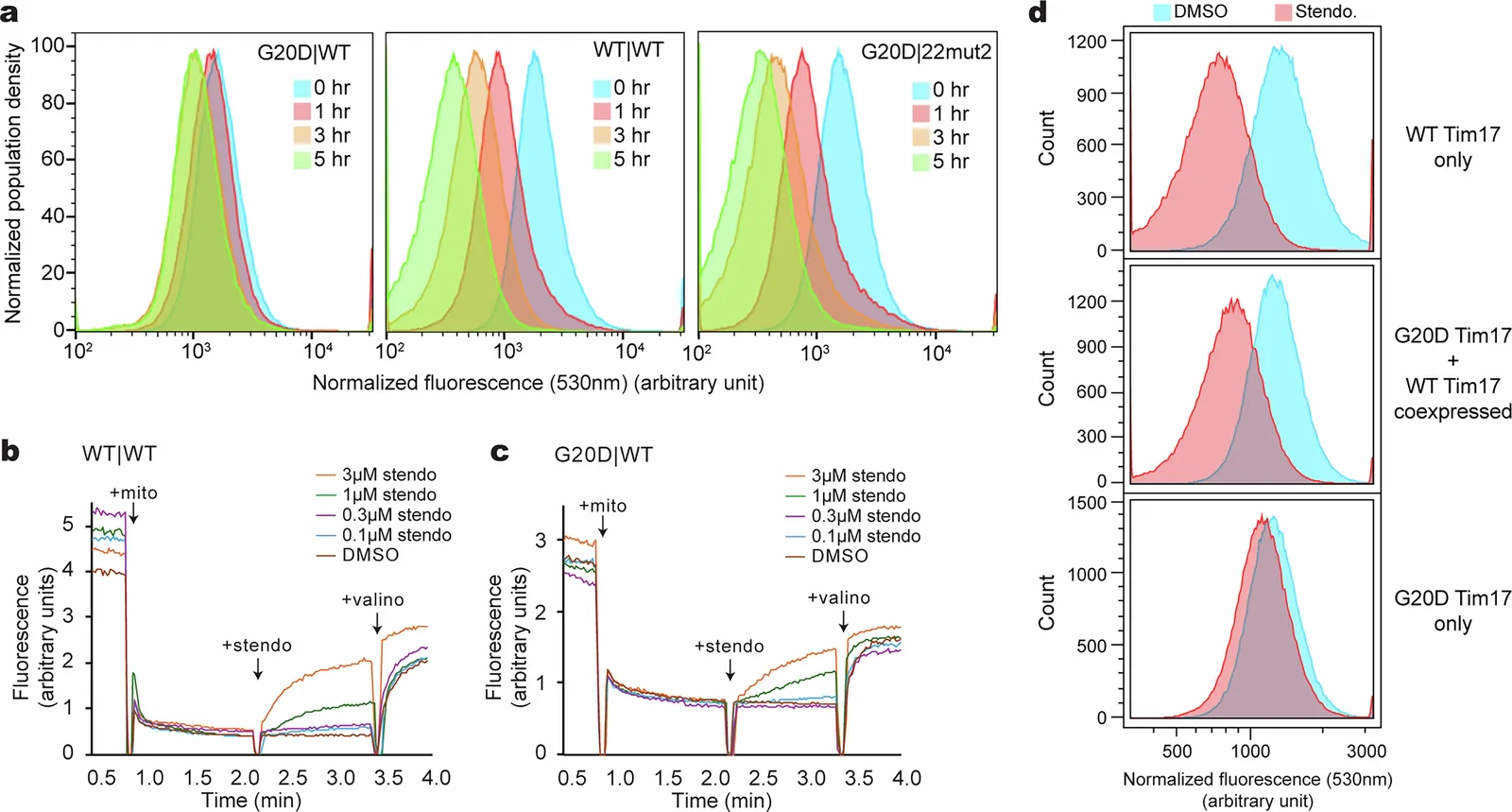
Mitochondrial membrane potential collapse upon stendomycin treatment. (**a**) Raw flow cytometry data showing fluorescence changes upon stendomycin treatment. Yeast cells growing in YPD medium were treated with 3 µM stendomycin for the indicated durations, stained with Rhodamine 123, and analyzed by flow cytometry as described in Methods. Fluorescence intensities (BL1-A) were normalized by side scatter values (SSC-A). (**b**) Effect of stendomycin on the membrane potential of isolated mitochondria from the WT|WT strain. (**c**) As in **b**, using mitochondria isolated from the G20D|WT strain. (**d**) As in **a**, but indicated yeast strains (WT W303, yKW976, and yKW977, respectively) were grown to an OD_600_ of ~0.5, and Δ*Ψ*_m_ was measured after treating cultures with 3 µM stendomycin or DMSO for 4 h.

Both the TIM23 and TIM22 complexes rely on Δ*Ψ*_m_ to drive protein translocation and membrane insertion^44,45^. The similar proteomic change patterns, despite targeted inhibition, suggested that Δ*Ψ*_m_ dissipation could be a common underlying driver of the perturbation. We therefore monitored Δ*Ψ*_m_ in intact yeast cells using flow cytometry following stendomycin treatment (Fig. 5f and Extended Data Fig. 8a). The results confirmed that WT|WT and G20D|22mut2 strains exhibited a substantial Δ*Ψ*_m_ decrease within 1 hour, followed by a further collapse over the next few hours. In contrast, the G20D|WT strain largely maintained Δ*Ψ*_m_, indicating that the effect depends on stendomycin binding to Tim17 or Tim22.

Previously, stendomycin collapsed Δ*Ψ*_m_ in mammalian cells at a micromolar concentration, whereas import inhibition occurs at submicromolar concentrations^32^. This raised the possibility that the mechanism of Δ*Ψ*_m_ collapse may not be directly related to import blockage. Therefore, we also measured Δ*Ψ*_m_ changes in purified mitochondria^46^. Both WT|WT and G20D|WT mitochondria showed similar rapid Δ*Ψ*_m_ collapse above 1 µM stendomycin, whereas little to no change in Δ*Ψ*_m_ was observed at <0.3 µM stendomycin, indicating ionic permeation at high stendomycin concentrations (Extended Data Fig. 8b,c). However, this leak is unlikely related to Δ*Ψ*_m_ collapse observed in the flow cytometry experiments given the Tim17 binding-independence and because intracellular stendomycin concentrations are probably substantially below 1 µM due to limited cell wall permeability. Yet, considering the dominant growth effect seen in Extended Data Fig. 3k, we reasoned that stendomycin binding to Tim17 or Tim22, rather than import blockage per se, may contribute to Δ*Ψ*_m_ collapse over time. Supporting this, stendomycin substantially decreased Δ*Ψ*_m_ in yeast coexpressing WT and G20D Tim17, confirming its dominant effects on Δ*Ψ*_m_ through Tim17 binding (Extended Data Fig. 8d).

**Extended Data Fig. 9:**
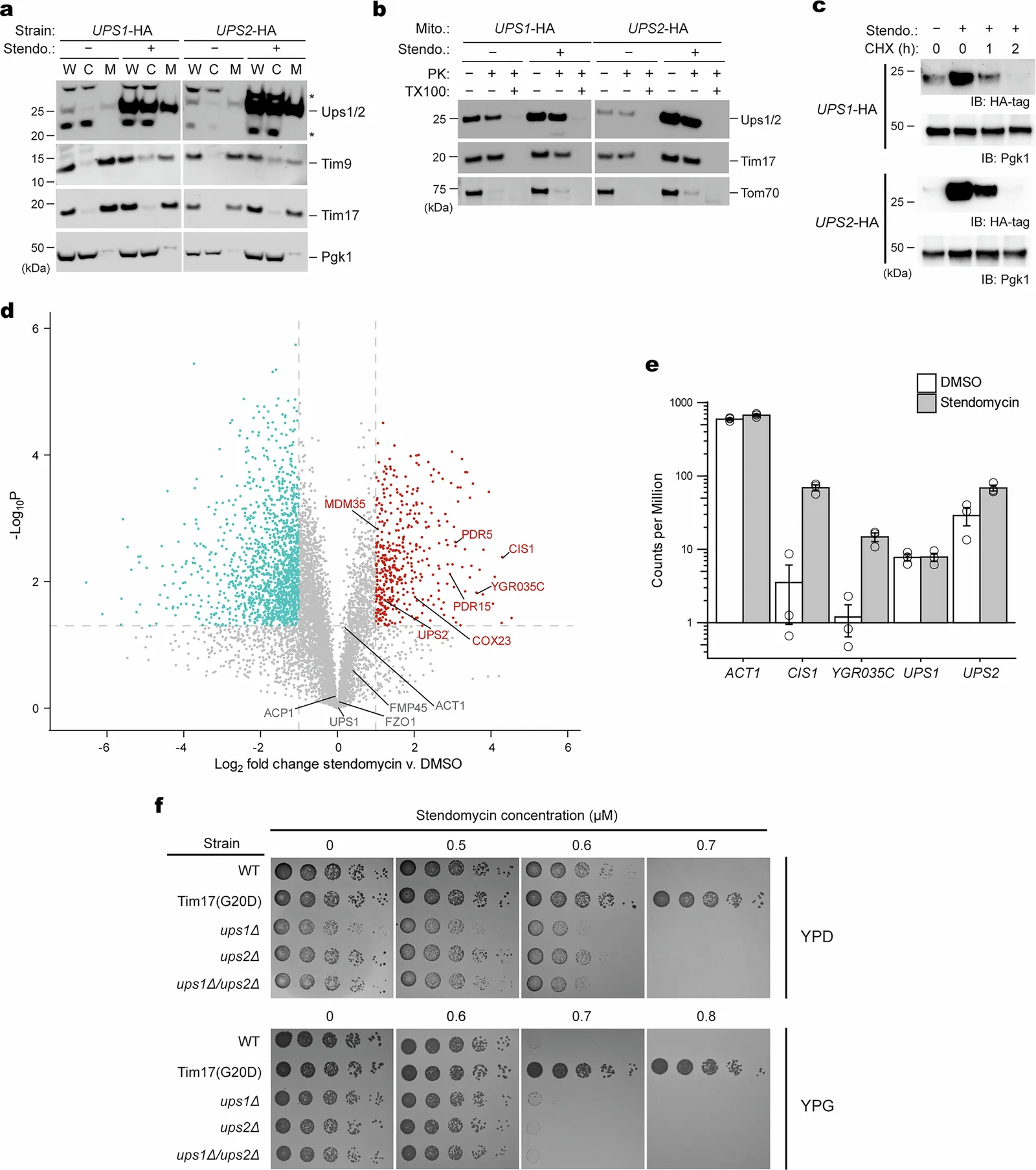
Upregulation of Ups1 and Ups2 upon stendomycin treatment. (**a**) Yeast strains with chromosomally HA-tagged *UPS1* and *UPS2* were treated with stendomycin for 3 h. Whole cell lysates (W) were subjected to fractionation to the cytosol (C) and mitochondria (M), and samples were analyzed by immunoblotting. Residual Tim9 in the cytosolic fraction might suggest incomplete import of Tim9 or decrease mitochondrial membrane integrity upon stendomycin treatment. α-Tim17 and α-Pgk1 blots are from the same gel. α-HA and α-Tim9 blots are from two separate gels loaded with equal volumes of the identical samples. (**b**) As in **a**, but the mitochondrial fraction was additionally subjected to proteinase K (PK) digestion. Where indicated, mitochondria were solubilized by Triton X-100 (TX100) before digestion. Tim17 and Tom70 were used for standards for IM and OM proteins. α-Tim17 and α-Tom70 blots were from the same gel, and α-HA blot is from a separate gel loaded with equal volumes of the identical samples. (**c**) The *UPS1-*HA and *UPS2-*HA cells were treated with cycloheximide (CHX) in the last 1 or 2 hours of the 3-h incubation with stendomycin prior to harvesting for SDS-PAGE and immunoblot analysis. Pgk1, loading control. Experiments in **a**–**c** are repeated twice with similar results. (**d**) Transcriptome-wide changes in WT yeast cells (RNA-seq) following 3 μM stendomycin treatment for 6 h. *x*-axis values (means of three independent experiments) represent transcriptional changes (normalized read counts) relative to the DMSO controls. (**e**) As in **d**, showing raw normalized read counts of indicated transcripts (means ± s.e.m. of three independent experiments). *ACT1* (actin) was included as a reference control. (**f**) Growth of WT, *ups1Δ*, *ups2Δ* and *ups1Δ*/*ups2Δ* strains was tested on YPD and YPG agar plates containing varying concentrations of stendomycin. Tim17-G20D mutant strain was included as a control. Source data

Among proteins altered in abundance by stendomycin, Ups1 and Ups2 stood out with pronounced (roughly tenfold) upregulation, regardless of whether Tim17 or Tim22 was targeted (Fig. 5a,b,g,h). Ups1 and Ups2 are small soluble IMS proteins of the conserved Ups/PRELI family that mediate phospholipid transfer between the OM and IM^47–50^, but their regulation by mitochondrial stress has not been reported. On stendomycin treatment, Ups1 and Ups2 accumulated in both cytosolic and mitochondrial fractions (Extended Data Fig. 9a,b). Cotreatment with cycloheximide abolished this upregulation, indicating dependence on active protein synthesis (Extended Data Fig. 9c). RNA sequencing (RNA-seq) analysis revealed only modest *UPS2* transcriptional induction (roughly twofold) and no change in *UPS1* (Extended Data Fig. 9d,e), pointing to a posttranscriptional or posttranslational mechanism. Δ*Ψ*_m_ dissipation might be an upstream trigger, as valinomycin treatment moderately elevated levels of both proteins (roughly fourfold) in YPD medium (Fig. 5g,h). Consistent with previous reports^50,51^, *ups1Δ* caused a mild growth defect in YPD medium, which could be suppressed by *ups2Δ* or by growth on YPG medium (Extended Data Fig. 9f; 0 μM stendomycin). At 0.6 µM stendomycin, the *ups1Δ*, *ups2Δ* and *ups1Δ/ups2Δ* strains all showed slightly reduced growth in YPD, but not YPG medium. Overall, these findings suggest that Ups1 and Ups2 upregulation may reflect a compensatory response to mitochondrial stress. Lastly, Mdm35, a binding partner of Ups1 and Ups2, was detected in our mass spectrometry experiments, but its levels moderately decreased following stendomycin treatment (Fig. 5a,b), suggesting that accumulated Ups1 and Ups2 likely exist in an Mdm35-free state.

## Discussion

**Fig. 6:**
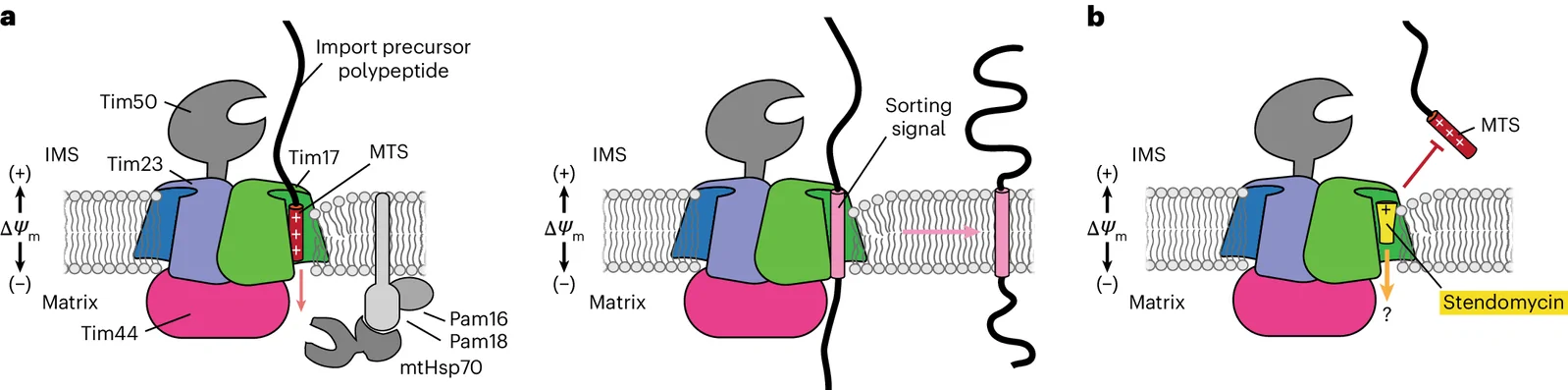
Roles of the Tim17 cavity in protein topogenesis and their inhibition by stendomycin. **a**, Normal functions of the TIM23 complex. Left: Tim17 locally thins the membrane within its cavity, facilitating the translocation of MTS of client polypeptides in a Δ*Ψ*_m_-dependent manner. The Tim17 cavity likely stabilizes the transition state in which the MTS is inserted across the IM. Right: some TIM23 client proteins contain a hydrophobic TM segment—also known as a sorting signal—located downstream of the MTS. This segment is inserted into the IM via the lateral opening of the Tim17 cavity, which may also stabilize the depicted intermediate state before insertion. **b**, Stendomycin binds stably within the Tim17 cavity, thereby inhibiting MTS engagement and subsequent import. In the presence of Δ*Ψ*_m_, stendomycin might translocate into the matrix on binding to Tim17, contributing to the dissipation of Δ*Ψ*_m_.

In this work, we used stendomycin, a natural-product inhibitor of the TIM23 complex, to study mitochondrial protein import mediated by the TIM23 and TIM22 complexes. Using cryo-EM, we first revealed that stendomycin binds in an α-helical conformation within the Tim17 cavity of the TIM23 complex, mimicking topogenic sequences as well as acting as a competitive inhibitor of import substrates (Fig. 6). We showed that with a small modification, Tim22, the catalytic subunit of the TIM22 complex, can also engage stendomycin. We further demonstrated that the Tim17 and Tim22 cavities tolerate substantial surface modifications while preserving their respective functions. Last, our study shows that stendomycin binding to either Tim17 or Tim22 collapses Δ*Ψ*_m_, ultimately disrupting the import activities of both complexes.

TIM23 and TIM22 have long been recognized as the central machineries for mitochondrial matrix and IM protein biogenesis, but their structural mechanisms have only recently been revealed. Previously, Tim23, the namesake subunit of TIM23, was believed to form a protein-conducting channel either as a homomultimer of Tim23 or a heteromer with Tim17 (refs. ^29–31^). Similarly, TIM22 was thought to form a channel composed of multiple Tim22 subunits^27,28^. However, recent cryo-EM structures have revealed that the protein translocase and insertase functions of these complexes are instead mediated by a single monomer of Tim17 or Tim22 (refs. ^20–22^). Our current study further validates this revised model.

Both Tim17 and Tim22 belong to the same protein family and share highly similar structures, with their four TMs forming a laterally open concave cavity within the IM. In the TIM23 complex, crosslinking experiments have confirmed that the Tim17 cavity is used in protein translocation^20,25,26^. While similar data are not yet available for TIM22, mutational studies suggest a critical role for the Tim22 cavity in its function^22^.

The stendomycin-bound Tim17 structure provides important clues about how the Tim17 and Tim22 cavities recognize topogenic sequences during protein topogenesis. Stendomycin adopts a ~2.5-turn helical conformation that snugly occupies the Tim17 cavity. Since inhibitors often mimic natural substrates, topogenic sequences of import clients may bind to the Tim17 and Tim22 cavities in a similar manner to drive topogenesis (Fig. 6). This includes MTSs, long thought to form an amphipathic helix^17,18,52^, and TM segments of client membrane proteins, which adopt a helical conformation as they integrate into the membrane. Interactions are likely strongest in the central hydrophobic region of the cavity, where ~2.5 turns can be accommodated. An analogous mechanism is observed in the endoplasmic reticulum translocon Sec61 complex, where inhibitors bind at an overlapping site that accepts a signal sequence^53,54^. The fact that stendomycin can bind to both Tim17 and Tim22 further indicates that the fitting is mediated by overall shape complementarity between the substrate’s α-helix and the concave cavity, rather than specific amino acid interactions. This would also explain how the Tim17 and Tim22 cavities can interact with diverse sequences of topogenic signals.

**Extended Data Fig. 10:**
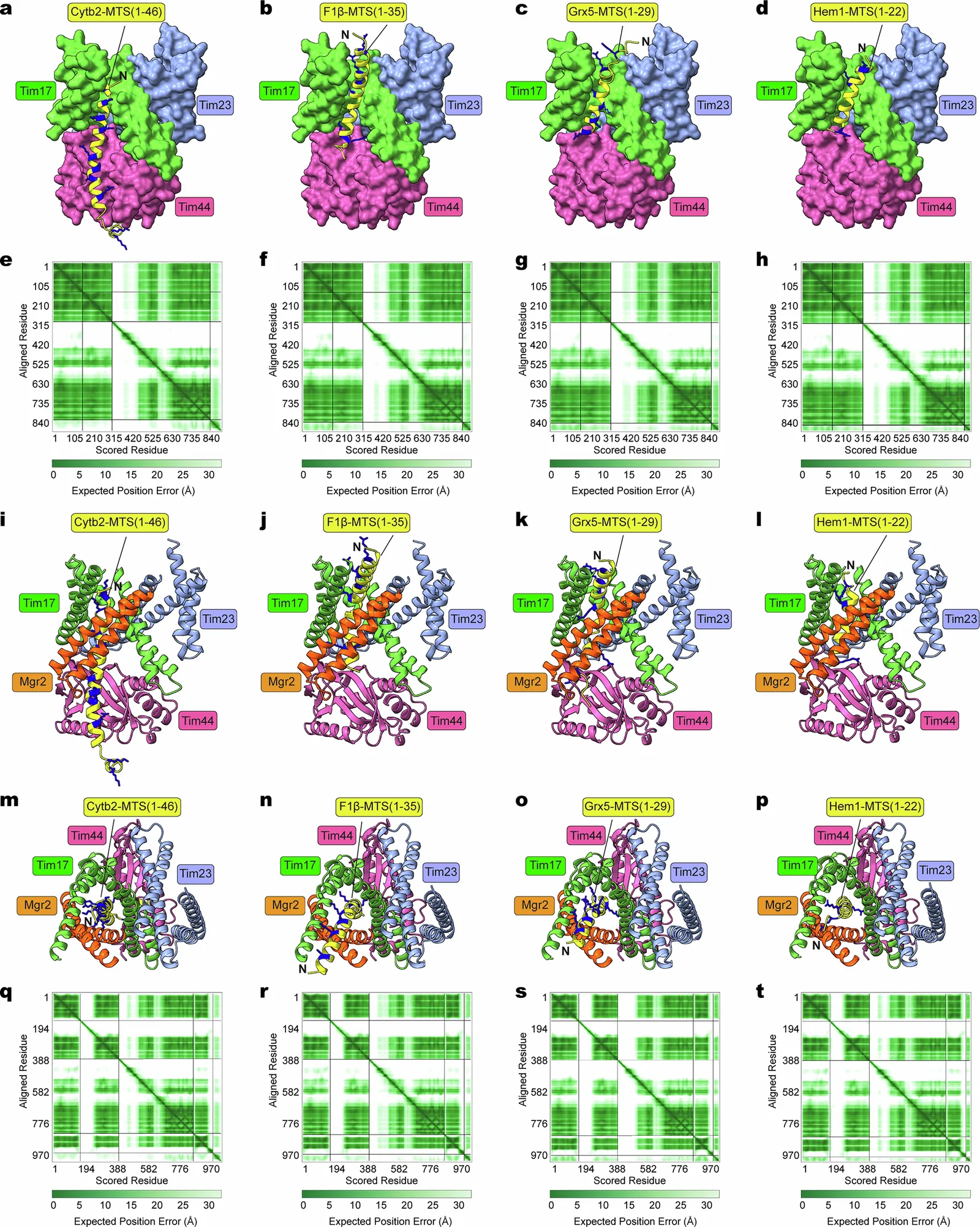
AlphaFold3 predictions of MTS peptides in the core TIM23 complex. (**a**–**d**) Four different MTS sequences (shown in yellow) were included in AlphaFold3 predictions of the Tim17-Tim23-Tim44 complex (displayed as a surface representation). Basic residues within MTS peptides are colored blue. For clarity, unstructured tails in the TIM23 complex are omitted. (**e**–**h**) Predicted aligned error (PAE) matrices corresponding to the predictions shown in **a**–**d**, Protein order: Tim17-Tim23-Tim44-MTS. (**i**–**l**) As in **a**–**d**, but with Mgr2 (orange) additionally included in the predictions. (**m**–**p**) As in **i**–**l**, but showing views from the IMS face. (**q**–**t**) the PAE matrices corresponding to the predictions shown in **i**–**p**. Protein order: Tim17-Tim23-Tim44-Mgr2-MTS.

Although experimental structures of MTS-bound TIM23 complexes will be needed for a detailed mechanistic understanding, AlphaFold3 predicts that MTSs would span the Tim17 cavity as an α-helix (Extended Data Fig. 10). These models have notable limitations, however: they are inconsistent with the expected N_matrix_–C_IMS_ orientation of MTSs and do not display consistent positioning of positively charged residues in MTSs relative to Tim17 and the lipid phase. It also remains unclear whether MTS binding is energetically favored for the lipid-exposed open cavity over the Mgr2-enclosed form. After submission of this work, a cryo-EM structure of Tim17-Mgr2 engaged with a hydrophilic translocating polypeptide was published^55^. Notably, while AlphaFold3 predictions placed helical MTSs within the Tim17–Mgr2 pore, this structure showed that the polypeptide is largely extended in the central hydrophobic constriction. As we observed for competition between Mgr2 and stendomycin, this region seems too narrow to accommodate an α-helix. Therefore, MTS recognition is more likely to occur through the lipid-exposed Tim17 cavity without Mgr2.

The ability of Tim22 variants to bind stendomycin and the retention of Tim17 and Tim22 functions after swapping large portions of the cavity-lining residues provide additional mechanistic insights. Despite their distinct client specificities and seemingly different functions, our findings suggest that both Tim17 and Tim22 share a common mechanism for driving topogenic processes. Although Tim17 can translocate long hydrophilic polypeptide segments across the IM, structural evidence suggests that both Tim17 and Tim22 originated as insertases. Forming a channel-like structure with Mgr2 likely enhances the translocase function of Tim17. However, since Mgr2 is not strictly required, the most fundamental function of the Tim17 cavity may not be facilitating the processive translocation of long polypeptides. Instead, initial insertion of topogenic sequences using the cavity, coupled with the electrophoretic driving force of Δ*Ψ*_m_, could be the most central function of Tim17 and Tim22. In the case of TIM23, following the initial translocation of the MTS, the presequence translocase-associated motor subcomplex further facilitates polypeptide translocation through the ATP-hydrolysis-driven actions of mitochondrial Hsp70 (refs. ^56,57^). In contrast, TIM22 lacks such a mechanism. Thus, the presence of presequence translocase-associated motor may be the key aspect distinguishing a translocase (TIM23) from an insertase (TIM22). In addition, distinct chaperone components on the IMS side, Tim50 in TIM23 and the small TIM complex in TIM22, may preselect their respective clients.

Previous studies, including our own, have shown that conserved acidic residues in the Tim17 and Tim22 cavities are functionally critical^20,22,25,26^. Their importance may arise from their role in local membrane thinning within the cavity or their ability to mediate electrostatic interactions with positively charged residues in client proteins, or both. While these possibilities are not mutually exclusive, our current data strongly support the local membrane thinning model, as increased hydrophobicity in the cavity correlates well with the severity of functional defects. In addition, acidic residue positions within the cavity could be varied substantially; as long as they remained on the IMS portion of the cavity, Tim17 retained its function. Overall, the charge density or hydrophilicity seems key for functionality. Membrane thinning may be important for the initial insertion of topogenic sequences or translocation of polar residues flanking these signals, or both. It may also facilitate the lateral release of clients’ TMs into the IM while preventing other single-spanning IM proteins from occupying the cavity, which could otherwise competitively inhibit topogenic processes.

Lastly, it is noteworthy that both cytotoxicity and Δ*Ψ*_m_ collapse are driven in part by a dominant component involving stendomycin binding to Tim17. One possibility is that partial import blockade leads to accumulation of unimported precursors, which in turn compromises Δ*Ψ*_m_ maintenance. However, this scenario is unlikely, as coexpression of an inactive Tim17 mutant alongside WT Tim17 did not produce dominant effects (Fig. 4b, −Dox). A more likely possibility is that stendomycin undergoes matrix translocation, analogous to MTSs, with each molecule carrying one positive charge down the potential gradient through its arginine-derived stendomycidine residue (Fig. 6b). Given the Δ*Ψ*_m_ dependency of TIM23 and TIM22 functions, mitochondrial depolarization would further impair import beyond the competitive inhibition mechanism of stendomycin. This combinatorial action may explain the exceptionally steep cell viability decline within a narrow stendomycin concentration range (for example, Fig. 3f).

In conclusion, our study reveals the structural basis of stendomycin-mediated mitochondrial protein import inhibition and provides mechanistic insights into the structure–function relationship of the evolutionarily conserved Tim17 and Tim22 cavities and their role in recognizing topogenic sequences. Important future directions include structure determination of topogenic sequence-bound Tim17 and Tim22, and elucidating mechanisms linking import inhibition or Δ*Ψ*_m_ collapse to downstream physiological effects, such as the upregulation of Ups1 and Ups2.

## Methods

### Materials and yeast growth media

Stendomycin was obtained as a mixture of congeners from *S. hygroscopicus* NRRL 2751 as described previously^34^. Peptidisc peptide was purchased from Peptidisc Laboratory (catalog number bulk peptidisc).

YPD medium was composed of 1% yeast extract, 2% peptone and 2% glucose. YPG medium was composed of 1% yeast extract, 2% peptone, 3% glycerol and 2% ethanol. Leu-drop out medium was composed of 5 g l^−1^ ammonium sulfate, 1.7 g l^−1^ yeast nitrogen base (United States Biological, catalog number 2030), 85.6 mg l^−1^ amino acids except Leu (United States Biological, catalog number D9539; uracil supplemented separately) and 2% glucose. For agar plates, 2% bacto-agar was included.

### Construction of plasmids and yeast strains

A list of yeast strains and plasmids used in this study is given in Supplementary Tables 2 and 3, respectively. Primers used are listed in Supplementary Table 4. The sequences of the plasmids and PCR products used in this study are included in Supplementary Table 5.

For purification of the core TIM23 complex (Tim17–23–44) under the *GAL1* promoter (Fig. 1c–e), the yeast strain ySS055 from a previous study^20^ was used. Procedures for generating other constructs and strains are described in Supplementary Information.

### Error-prone PCR and screening for stendomycin-resistant mutants

The coding sequence (CDS) of Tim17 was amplified by error-prone PCR using a Taq polymerase with 0.5 mM Mn^2+^, increased concentration (7 mM) of Mg^2+^, unequal concentrations of deoxynucleotide triphosphates (1 mM for deoxycytidine triphosphate and deoxythymidine triphosphate, 0.2 mM for deoxyadenosine triphosphate and deoxyguanosine triphosphate) as previously described in ref. ^59^. Ten cycles of amplification (primers used: numbers 2835 and 2836) were performed such that on average single or double mutations were introduced to the CDS. The PCR products were then ligated into pYC17a using restriction enzymes *Kpn*I and *Spe*I and introduced to TOP10 *Escherichia coli* cells (Invitrogen) by transformation. Roughly 4,600 *E. coli* colonies from multiple agar plates were collected, and plasmids were isolated. The plasmids were then transformed into yYC17b and selected on YPD agar plates with 2 µM stendomycin and 100 μg ml^−1^ nourseothricin. Stendomycin-resistant colonies were picked, and plasmids isolated from selected clones were sequenced by the Sanger method.

### Purification of stendomycin-bound TIM23

The core TIM23 complex was purified as described previously in ref. ^20^. Briefly, yeast strain ySS055 was grown in YPG medium in shaker flasks at 30 °C. Cells were induced with 50 nM β-estradiol on reaching an optical density at 600 nm (OD_600_) of ~0.7–1.0. After 13–15 h of induction, cells were collected by centrifugation. Cells were washed with deionized water, resuspended in prewarmed dithiothreitol (DTT)-containing buffer (100 mM Tris-H_2_SO_4_ pH 9.4 and 10 mM DTT; 2 ml g^−1^ wet weight of cells) and incubated at 30 °C for 30 min to 1 h. Cells were then collected by centrifugation (3,000*g* for 5 min), washed with spheroplasting buffer (20 mM potassium phosphate pH 7.4 and 1.2 M sorbitol; 7 ml g^−1^ wet weight of cells) and resuspended in the buffer containing homemade lyticase. After incubation at 30 °C for 1–2 h with lyticase, cells were collected by centrifugation (3,000*g* for 5 min), washed with the spheroplasting buffer and resuspended in ice-cold homogenization buffer (10 mM Tris-HCl pH 7.4, 0.6 M sorbitol, 1 mM EDTA and 2 mM phenylmethyl sulfonyl fluoride (PMSF); 6 ml g^−1^ wet weight of cells). All subsequent steps were performed at 4 °C. The yeast spheroplasts were then homogenized with 40 strokes of a Dounce homogenizer and diluted twofold with the homogenization buffer. Crude mitochondria were subsequently isolated through steps of differential centrifugation. The suspension was first centrifuged at 1,500*g* for 5 min to remove cell debris and nuclei, and centrifuged again at 4,000*g* for 5 min. The supernatant from this step was then subjected to further centrifugation at 20,000*g* for 20 min (Beckman JA-17 rotor) to isolate the mitochondrial fraction. The mitochondrial pellet was washed with a buffer (buffer SEM) containing 10 mM MOPS-KOH pH 7.2, 250 mM sucrose and 1 mM EDTA, and collected at 17,000*g* for 20 min. The supernatant was removed, and the mitochondrial pellets (~500 mg each) were frozen in liquid nitrogen and stored at −80 °C until use.

To purify the core TIM23 complex, mitochondrial pellets were first thawed and resuspended in ice-cold lysis buffer (buffer L; using 3× volume of the mitochondrial pellet volume) containing 50 mM Tris-HCl pH 7.5, 200 mM NaCl, 1 mM EDTA, 10% glycerol, 5 µg ml^−1^ aprotinin, 5 µg ml^−1^ leupeptin, 1 µg ml^−1^ pepstatin A and 2 mM PMSF. All subsequent steps were carried out at 4 °C. The suspension was briefly homogenized with ten strokes of a Dounce homogenizer. Mitochondria were then solubilized with a detergent mixture of 0.4% glycol-diosgenin (GDN) (Anatrace) and 0.6% lauryl maltose neopentyl glycol (Anatrace). After a 1.5 h incubation, the lysate was clarified by centrifugation at 20,000*g* for 30–45 min (Beckman JA-17 rotor) and the supernatant was incubated with Sepharose beads conjugated with anti-BC2 nanobody^60^ for 2 h. The beads were then washed with approximately 30 column volumes of a wash buffer (buffer W) containing 25 mM Tris-HCl pH 7.5, 100 mM NaCl, 1 mM EDTA and 0.02% GDN. The TIM23 complex was eluted by incubating the beads with ~50 µg ml^−1^ HRV 3C protease for 1 h. Cleaved protein was concentrated using an Amicon Ultra (100,000 cutoff; Cytiva). The complex was then reconstituted to peptidiscs by mixing with the NSPr peptide (Peptidisc Laboratory) at a ratio of 1.2:1 (w/w, TIM23/NSPr) and incubated on ice for 45 min. Then, anti-Tim44 Fab was added to the complex at a molar ratio of 1:2 (TIM23/Fab), and the mixture was incubated on ice for another 15 min. The sample was then injected into a Superose 6 Increase 10/300 GL column (Cytiva) equilibrated with buffer W, containing 25 mM Tris-HCl pH 7.5, 100 mM NaCl, 1 mM EDTA and 0.02% GDN. Peak fractions were pooled and concentrated to ~4 mg ml^−1^ using an Amicon Ultra. Next, 100 μM of stendomycin was added on ice for 1 h. Last, 3 mM fluorinated fos-choline-8 (Anatrace) was added to the sample before cryo-EM grid preparation.

### Cryo-EM grid preparation and data acquisition

To prepare cryo-EM grids, 3 μl of the sample were applied to a glow-discharged (PELCO easiGlow; 0.39 mBar, 25–30 mA, 40–45 s) gold holey carbon grid (Quantifoil R 1.2/1.3, 400 mesh). The grid was blotted for 4 s and plunge-frozen in liquid-nitrogen-cooled liquid ethane using Vitrobot Mark IV (FEI) operated at 4 °C and 100% humidity. Whatman No. 1 filter paper was used to blot the samples.

The dataset was collected on a Titan Krios G3i electron microscope (FEI), operated at an acceleration voltage of 300 kV and equipped with a Gatan Quantum Image Filter (slit width of 20 eV). Dose-fractionated images (50 electrons per Å^2^ applied over 50 frames) were recorded on a K3 direct electron detector (Gatan) using the super-resolution mode at the physical pixel size of 1.05 Å. The datasets were collected using SerialEM^61^ with image-beam-shift multiple recording (18 recordings per stage movement). Coma induced by image-beam shift was corrected by beam tilt compensation in SerialEM. Target defocus values were set from 0.8 μm to 1.6 μm.

### Cryo-EM data analysis

For the summary of cryo-EM image processing, see Extended Data Fig. 1f. Briefly, the videos were processed for motion correction, contrast transfer function estimation and automated particle picking using Warp^62^. A minor fraction of micrographs were discarded due to ice contamination. From the 3,348 micrographs, 523,352 particles (box size of 256 pixels) were picked and extracted in Warp. Particle images then were imported into cryoSPARC v4 (ref. ^63^), and two-dimensional (2D) classification, ab initio reconstruction and heterogenous refinement were performed. One class (Class 1; 115,916 particles) from the heterogeneous refinement showed good density features for the core TIM23 complex and bound Fab molecule. This class was used to generate 2D templates for repicking particles in cryoSPARC. The videos were reprocessed in cryoSPARC for tile-based motion correction and contrast transfer function estimation. In total, 1,029,852 particles were picked, and their images (box size of 256 pixels) were extracted from 3,348 micrographs in cryoSPARC. Particle images were then subjected to a series of classification and refinement as shown in Extended Data Fig. 1f. The final reconstruction was refined to a nominal resolution of 3.2 Å from 105,256 particles. DeepEMhancer^64^ was used to sharpen and filter the map shown in Fig. 1.

### Atomic model building

An atomic model of apo core TIM23 (Protein Data Bank (PDB) 8SCX) was fitted into the sharpened map generated by cryoSPARC’s nonuniform refinement using UCSF Chimera^65^. Initial atomic coordinates for stendomycin were generated using the Grade2 web server (version 1.3.1rc1; https://grade.globalphasing.org) and fitted into the density feature of stendomycin in the map. We used the stendomycin IV structure, which has a minor difference in the acyl tail compared with stendomycin I, as a representative of a mixture of congeners^34,66^. The models were inspected and subjected to multiple rounds of local refinement in Coot^67^. Then, model refinement was performed using the phenix.real_space_refine program of Phenix^68^ with the refinement resolution limit set to the overall resolution of the map (the default sharpened map from cryoSPARC was used). Additional planar restraints were applied to the stendomycidine side chain of stendomycin during the refinement. Structural model validation was performed using MolProbity^69^ in the Phenix package (Supplementary Table 1). Protein electrostatics were calculated using the Adaptive Poisson-Boltzmann Solver^70^ with default parameters (with monovalent ion concentrations of 0.15 M each) built in PyMOL (Schrödinger). UCSF Chimera and PyMOL were used to prepare structural figures in the paper.

### Yeast growth assay

In the liquid-culture-based experiments, yeast strains W303 (Extended Data Fig. 1a) and yYC52 to yYC55 (Fig. 3f and Extended Data Fig. 6a) were first grown to an OD_600_ of ~0.8–1.0 in YPD. Cells were then diluted to an OD_600_ of 0.1 with fresh YPD containing indicated concentration of stendomycin or dimethylsulfoxide (DMSO) as a control, and grown for 15 h at 30 °C with shaking (220 rpm). OD_600_ was measured for each sample and normalized with respect to the value from the DMSO control. Dose–response curve and IC_50_ were calculated using R software (version 4.5.0) and ggplot2 (version 3.5.1) and drc (version 3.0-1) packages, fitting into the four-parameter log-logistic function model (LL.4) in the drc package. For the experiments in Extended Data Fig. 6b–d, overnight YPD cultures of yYC52, W303 and yYC56 were diluted to an OD_600_ of 0.2 with fresh YPD containing 3 μM stendomycin and grown at 30 °C. OD_600_ was measured at indicated time points.

In agar plate-based experiments (spot assays), starter cultures were grown at 30 °C to an OD_600_ of 0.7–1.5 in YPD medium under appropriate selection (100 μg ml^−1^ nourseothricin) where indicated, and then diluted to an OD_600_ of 0.1 in fresh media. After fivefold serial dilution, 5 μl were spotted on agar plates. The plates were incubated at the indicated temperature (30 °C unless indicated otherwise) for 2–3 days before imaging. The following strains and agar medium were used for specific experiments: (1) Fig. 2e and Extended Data Fig. 3i: yeast strain yYC17b with the pYC17a plasmid (WT or mutant) on YPD-nourseothricin with or without 2 μM stendomycin; (2) Fig. 3e,h and Extended Data Figs. 3g and 4c,e,k: yeast strains yYC17b and yYC22 transformed with plasmids pYC17a and pYC22, respectively, on YPD or YPG agar plates with 100 μg ml^−1^ nourseothricin, and with or without 10 μg ml^−1^ doxycycline; (3) Extended Data Fig. 4g: yeast strains W303 and yYC52–yYC56 on YPD plates with or without 2 μM stendomycin; (4) Extended Data Fig. 4j: yeast strains yYC17b transformed with pYC17a, on YPD-nourseothricin agar plates containing 10 μg ml^−1^ doxycycline and with or without 2 μM stendomycin; (5) Fig. 4: yYC17b transformed with pYC17a on YPD-nourseothricin plates with or without 10 μg ml^−1^ doxycycline; and (6) Extended Data Fig. 9f: yeast strains W303, yYC52 (TIM17(G20D)), yYC59 (*ups1Δ*), yYC60 (*ups2Δ*) and yYC61 (*ups1*Δ/*ups2*Δ) on YPD or YPG containing the indicated concentration of stendomycin.

### In vitro mitochondrial protein import assay

C-terminally His-tagged Cyb2MTS-Cyb2Δ-DHFR, Grx5MTS-Cyb2Δ-DHFR and F1bMTS-Cyb2Δ-DHFR were expressed in *E. coli* and purified as described in a previous study^20^. In brief, *E. coli* BL21(DE3) was transformed with pYC002, pYC101 or pYC102. Cells were grown in Luria-Bertani medium to an OD_600_ of ~0.6, and the expression was induced with 0.5 mM isopropylthio-β-galactoside overnight at 10 °C. Cells were lysed by sonication in 40 mM Tris-HCl pH 7.5, 300 mM NaCl, 1 mM PMSF, 10 mM DTT and 0.1% Triton X-100. After clarification (Sorvall SS-34 rotor; 1 h at 23,000 *g*), proteins were purified with cobalt resin (Thermo Scientific, catalog number 89966). The eluate was dialyzed against 0.1 M sodium phosphate buffer pH 6.5 and further purified by a HiTrap SP HP column (Cytiva) and a NaCl gradient elution. Purified substrates were concentrated to 0.25 mg ml^−1^ in a storage buffer (10 mM MOPS-KOH pH 7.2, 150 mM NaCl, 1 mM EDTA and 10% glycerol), frozen and stored at −80 °C. Mitochondria were isolated as described above using yeast cells (W303 for Fig. 1b and Extended Data Fig. 1b, and yYC64–yYC66 for Fig. 3i) grown to an OD_600_ of ~0.8–1.0 in YPG medium. Isolated mitochondria were frozen at −80 °C at 5 mg ml^−1^ (based on the Bradford assay using BSA as control) in buffer SEM until use.

For protein import assays, 200 μg of mitochondria was mixed with 4 μg indicated substrate in 400 μl of import assay buffer (10 mM MOPS-KOH pH 7.2, 250 mM sucrose, 80 mM KCl, 5 mM MgCl_2_ and 3% w/v BSA) and supplemented with 2 mM ATP, 2 mM NADH and 2 μM methotrexate (MTX; TCI America, catalog number M16641). Where indicated, mitochondria were preincubated with 2 μM valinomycin (Sigma Aldrich, catalog number V0627) for 5 min on ice to dissipate the membrane potential, before setting up the import reactions. After a 20-min incubation at 25 °C, mitochondria were pelleted and washed twice with the SEM buffer. Samples were analyzed by SDS–PAGE and immunoblotting.

### Co-IP

For the experiment in Fig. 2g, the yeast strain yKW936 and yKW937 were transformed with pYC007, a *CEN6*-*ARS4* plasmid that expresses the stall-inducing substrate Grx5-S80-sfGFP under an inducible *GAL1* promoter^20^. Cells were initially cultured in leucine-drop out synthetic medium containing 2% glucose. Substrate expression was induced with 50 nM β-estradiol for 2 h at 30 °C. Crude mitochondria were isolated as previously described and incubated with 1.5 µM stendomycin or DMSO for 30 min at 25 °C. Following incubation, samples were solubilized in buffer L containing 1% digitonin (Calbiochem, catalog number 300410) for 1.5 h at 4 °C. The resulting lysate was cleared by centrifugation and the supernatant was incubated with BC2 nanobody-conjugated beads for 1 h at 4 °C. Beads were subsequently washed with buffer W containing 0.05% digitonin, and bound proteins were eluted in SDS sample buffer for analyzed by immunoblotting.

### Proteomics

To prepare samples for mass spectrometry, yeast strains W303, yYC52 and yYC56 were grown in YPD to an OD_600_ of ~0.2 and grown for another 6 h after adding 3 µM stendomycin or DMSO. For whole-cell samples, yeast cells were pretreated with 0.1 M NaOH for 20 min on ice and then resuspended in buffer L2 (0.2 M 4-(2-hydroxyethyl)piperazine-1-propanesulfonic acid pH 8.5, 8 M urea and cOmplete protease inhibitor cocktail (Roche)). The cells were lysed by bead beating, and solubilized with 0.5% *n*-dodecyl-D-maltoside (DDM). For mitochondrial samples, crude mitochondria were prepared as described above and solubilized in buffer L2 containing 0.5% DDM. Cleared lysates were frozen for mass spectrometry.

Protein concentrations were determined by Bradford assay. Protein extracts (100 µg for whole extracts, 50 µg for mitochondria-enriched extracts) were subjected to disulfide bond reduction with 5 mM tris(2-carboxyethyl)phosphine (10 min at 55 °C) and alkylation with 20 mM iodoacetamide (15 min at room temperature). Methanol–chloroform precipitation was performed before protease digestion. In brief, four parts of neat methanol were added to each sample and vortexed. One part of chloroform was then added to the sample and vortexed. Finally, three parts of water were added to the sample and vortexed. The sample was centrifuged at 20,000*g* for 5 min at room temperature and washed twice with 100% methanol. Samples were resuspended in 100 mM EPPS (pH 8.5) containing 0.1% RapiGest and digested overnight at 37 °C with trypsin at a 100:1 protein/protease ratio. The digestion efficiency of a small aliquot was tested. Samples were then subjected to tandem mass tag (TMT) labeling.

Proline-based reporter isobaric TMT (TMTpro) labeling of peptide samples resuspended in 100 mM EPPS pH 8.5, was carried out as follows. TMTpro reagent was added to samples, along with acetonitrile to achieve a final acetonitrile concentration of approximately 30% (v/v). Following incubation at room temperature for 1 h, the labeling efficiency of a small aliquot was tested for each set and the reaction was then quenched with hydroxylamine to a final concentration of 0.5% (v/v) for 15 min. The TMTpro-labeled samples were pooled together at a 1:1 ratio. The sample was vacuum centrifuged to near dryness and subjected to C18 solid-phase extraction. Downstream liquid chromatography with tandem mass spectrometry and data analysis procedures are described in Supplementary Information.

### Transcriptome analysis (RNA-seq)

WT W303 cells were grown to an OD_600_ of ~1.0 in YPD medium, diluted to an OD_600_ of 0.2 and cultured further in YPD supplemented with 3 µM stendomycin or DMSO for 6 h. Cells (5 OD_600_ units) were collected by centrifugation at 3,000*g* and washed once with distilled water. Total RNA was extracted using a Monarch Spin RNA Isolation Kit (New England Biolabs, catalog number T2010S) following the manufacturer’s protocol, with mechanical lysis performed using glass beads. RNA integrity was verified by agarose gel electrophoresis. A total of 400–600 ng of RNA from each sample (three independent biological replicates per condition) was subjected to Illumina sequencing using a 3′-end counting approach (Plasmidsaurus). The three DMSO-treated and three stendomycin-treated samples yielded approximately 5.7, 5.4, 5.8, 6.5, 5.8 and 5.8 million deduplicated, uniquely mapped reads, respectively. Data were analyzed and visualized in RStudio using counts per million as the unit of measurement.

### Mitochondrial membrane potential assays

For flow cytometry-based assays (Fig. 5f and Extended Data Fig. 8a), yeast strains W303, yYC52 and yYC56 were grown in YPD to an OD_600_ of ~1.0 and treated with 3 µM stendomycin for indicated time. Equal numbers of cells (0.05 OD_600_ units) were collected for each strain at each time point, washed with deionized water and stained with 200 nM Rhodamine-123 (Rh123; Biotium catalog number 70010) for 10 min at room temperature as described previously in ref. ^71^. Flow cytometry analysis was performed on Attune NxT Acoustic Focusing Cytometer (Invitrogen) with excitation at 488 nm and emission at 530 nm (30-nm bandpass). The data were analyzed with FlowJo v.10.10.0. Single cells were selected by a standard gating procedure using FSC-H versus FSC-A plots, and the derived value BL1-A/SSC-A was plotted. For Fig. 5f, median fluorescence intensity at each time point was normalized with respect to the value at *t* = 0. For Extended Data Fig. 8d, yeast cells were grown in YPD to an OD_600_ of 0.5 and treated with 3 µM stendomycin for 4 h. Rhodamine-123 staining and flow cytometry analysis were performed as described above.

For membrane potential measurement using isolated mitochondria (Extended Data Fig. 8b,c), crude mitochondria were isolated from yeast strains W303 or yYC52 as described above. Experiments were conducted in a reaction buffer (20 mM potassium phosphate pH 7.4, 600 mM sorbitol, 10 mM MgCl_2_, 0.5 mM EDTA, 0.1% (w/v) BSA) at 25 °C (ref. ^46^). The following components were added in the given order while fluorescence at 670 nm on excitation at 622 nm was monitored with a spectrofluorometer (Photon Technology International): 1 µM 3,3′-dipropylthiadicarbocyanine iodide (DiSC3(5); TCI America catalog number D4456), 1 mM NADH, 1 mM ATP, 15 µg mitochondria, stendomycin at indicated concentration and 2 µM valinomycin (Sigma Aldrich; catalog number V0627).

### Mitochondrial fractionation and protease protection assay

To determine the submitochondrial localization of Ups1 and Ups2 (Extended Data Fig. 9a), yeast strains yYC57 and yYC58 were grown in YPD medium to an OD_600_ of ~1.0 at 30 °C. Cells were treated with either DMSO (control) or 1.5 µM stendomycin for 3 h at 30 °C. Mitochondria were isolated using a modified fractionation protocol. Briefly, ~20 OD_600_ units of cells were collected by centrifugation (3,000*g*, 10 min), washed with water and incubated in DTT-containing buffer (100 mM Tris-H_2_SO_4_ pH 9.4, 10 mM DTT) for 15 min at 30 °C. Spheroplasts were generated by resuspending cells in buffer containing 20 mM potassium phosphate pH 7.4, 1.2 M sorbitol and homemade lyticase for 15 min at 30 °C. Spheroplasts were briefly spun down and resuspended in ice-cold homogenization buffer (0.6 M sorbitol, 10 mM Tris-HCl pH 7.4, 1 mM EDTA, 1 mM PMSF and 0.2% BSA) and disrupted using 15 strokes in a precooled Dounce homogenizer with a tight-fitting pestle. The resulting homogenate was centrifuged at 600*g* for 10 min at 4 °C to remove intact cells and nuclei. The supernatant was further centrifuged at 6,500*g* for 10 min at 4 °C to yield a crude mitochondrial pellet and a cytosolic supernatant fraction.

For proteinase K protection assays (Extended Data Fig. 9b), mitochondrial pellets were resuspended in buffer SEM. Proteinase K treatments were performed in 40 µl of reaction volumes at a final concentration of 50 µg ml^−1^ proteinase K, in the presence or absence of 0.5% Triton X-100. Samples were subsequently analyzed by SDS–PAGE and immunoblots.

### Immunoblotting and antibodies

Expression levels of proteins for yeast growth assays were determined by diluting overnight yeast cultures in YPD or YPG medium with appropriate selection (100 μg ml^−1^ nourseothricin) where indicated and growing them to a mid-log phase at 30 °C. Equal numbers of cells (0.5 OD_600_ units) were collected and lysed in a NaOH lysis solution (0.1 M NaOH, 50 mM EDTA, 2% SDS and 2% β-mercaptoethanol) with heating. The samples were neutralized with 0.1 M acetic acid and analyzed by SDS–PAGE.

For experiments in Fig. 5g,h, yeast strains yYC57 and yYC58 were grown in YPD or YPG to an OD_600_ of ~1.0. The cells were treated with 0.5% DMSO, 45 µM valinomycin, 3 µM stendomycin or no treatment for 4 h at 30 °C before being collected and lysed as described above.

For experiments in Extended Data Fig. 9c, yeast strains yYC57 and yYC58 were grown in YPD to an OD_600_ of ~1.0. The cells were treated with DMSO or 3 µM stendomycin for 3 h at 30 °C. During this period, 0.25 mg ml^−1^ cycloheximide was added for the indicated time.

Immunoblotting experiments were performed using antibodies against Pgk1 (a gift from J. Thorner; 1:1,000 dilution), Tim17 and Tim9 (gifts from C. Koehler; 1:250 dilution), Tom70 (a gift from N. Pfanner; 1:250), hemagglutinin-tag (Invitrogen, catalog number 26183; 1:5,000 dilution), His-tag (Invitrogen, catalog number MA1-21315, 1:1,000 dilution) and GFP-tag (Proteintech, catalog number 66002-1-IG; 1:20,000 dilution). For Spot-tag blots, we used homemade BC2-nanobodies, which were described previously^20^. Secondary antibodies conjugated to horseradish peroxidase used in this study were goat anti-rabbit (Thermo Scientific, catalog number 31460; 1:10,000 dilution) and goat anti-mouse (Thermo Scientific, catalog number 31430; 1:10,000 dilution).

### Reporting summary

Further information on research design is available in the Nature Portfolio Reporting Summary linked to this article.

## Online content

Any methods, additional references, Nature Portfolio reporting summaries, source data, extended data, supplementary information, acknowledgements, peer review information; details of author contributions and competing interests; and statements of data and code availability are available at https://doi.org/10.1038/s41589-026-02304-z.

## Supplementary information

Supplementary Information Supplementary Methods and Tables 1–5.

Reporting Summary.

## Source data

Source Data Figs. 1, 3 and 5. Statistical source data.

Source Data Figs. 2, 3 and 5, and Source Data Extended Data Figs. 1, 3–5 and 9. Uncropped gel and blot images.

## Data Availability

The cryo-EM map and atomic model of the stendomycin-bound TIM23 complex are available from the Electron Microscopy Data Bank (accession no. EMD-49111) and PDB (accession ID 9N84). The MS TMTpro proteomic data have been deposited in the MassIVE repository under the dataset identifier MSV000098464. The RNA-seq data have been deposited in the National Center for Biotechnology Information Gene Expression Omnibus repository under the accession ID GSE336173. Data are available from the corresponding author upon request. Source data are provided with this paper.
