# Supplementary material for: Topogenic sequence recognition at TIM complexes revealed by a stendomycin-bound structure
Source: Nat Chem Biol. 2026 Aug 26;22(11):1863–74. doi: 10.1038/s41589-026-02304-z (PMC13710162; doi:10.1038/s41589-026-02304-z)
Supplement: Supplementary file 1 [file 41589_2026_2304_MOESM1_ESM.pdf]

nature chemical biology

Supplementary information https://doi.org/10.1038/s41589-026-02304-z

Topogenic sequence recognition at TIM complexes revealed by a stendomycin-bound structure

In the format provided by the authors and unedited

Supplementary Methods

Construction of plasmids and yeast strains

For yeast growth complementation assays for Tim17 and Tim22, yeast strains yYC17b (ref. [20]) and yYC22 were used, where the chromosomal expression of Tim17 or Tim22 can be repressed in the presence of doxycycline. To generate yYC22, the tetracycline response element (TRE) and KanMX cassette were amplified from the genome of yeast strain TH_5187 (Horizon Discovery; cat. #YSC1180-202219224) by a 2-step PCR with primers #3305/3306, and #3307/3308 (all primers were synthesized by Integrated DNA Technologies), transformed into the SS150 strain by a standard lithium acetate/polyethylene glycol transformation method and selected on YPD agar plates with 300 µg/mL G418. Chromosomal integration was confirmed by PCR (primers used: #3309 and #3310).

For testing stendomycin sensitivity of Tim22 mutants, the endogenous Tim17 was replaced with Tim17(G20D) in W303 and yYC22, generating yYC52 and yYC51, respectively. G20D mutation was first introduced to pYC17a by PCR-based site-directed mutagenesis. The gene fragment spanning G20D was amplified by PCR (primers used: #1702 and #2955, for DNA sequence, see TIM17[G20D]), transformed into W303 or yYC22, and selected on YPD agar plates with 2 µM stendomycin. For yeast growth assay with endogenous Tim22 mutants and mass spectrometry, strains yYC53, yYC54, yYC55, and yYC56 were generated with a similar strategy. Plasmids pYC22, pYC109, pYC110, pYC111 were used as templates to amplify gene fragments spanning Tim22-2xHA (WT, 22mut1, 22mut2 and T52G, respectively) and NatMX by a 2-step PCR with #4764/4765 and #4764/4766. These gene fragments were then transformed into yYC52, and selected with YPD agar plates with 100 µg/mL nourseothricin. All the strains with altered endogenous TIM17 or TIM22 were confirmed by Sanger sequencing. For testing a dominant component of stendomycin sensitivity, yeast strain yKW976 and yKW977 were generated. These strains were made from yYC52 by integrating an extra copy of Tim17-2xHA under a constitutive *HHF1* promoter into the *HO* locus (integration plasmids pKW860 for WT Tim17 and pKW861 for G20D Tim17 were used).

For co-immunoprecipitation assays (Fig. 2g), the parent strain yMLT62 (a gift from J. Thorner) was modified to generate yKW936 (*TIM17::2xSPOT*) and yKW937 (*TIM17::2xMyc*). Specifically, PCR products encoding either a 2xSPOT or a 2xMyc tag (for DNA sequence, see TIM17-2xSpot and Tim17-2xMyc) were transformed into yMLT62 to tag the endogenous *TIM17* at its C-terminus via homologous recombination. Transformants were selected on YPD agar plates supplemented with 500 ug/mL hygromycin B. The resulting strains were further modified by introducing pYC112, linearized with *Not*I endonuclease to integrate and express N-terminally 2xHA-tagged Mgr2 from the *HO* locus. Colonies were selected on YPD agar plates with 300 µg/mL G418 and confirmed by PCR. Lastly, plasmid pYC007 from previous study[20] was transformed into yKW936 and yKW937 to enable the inducible expression of a stalled Tim17 substrate (Grx5-S80-sfGFP) under a *GAL1* promoter.

For in-vitro protein import assays (Fig. 3i), the plasmids pYC113, pYC114, and pYC115 were used as templates to amplify gene fragments spanning Tim17-2xSpot (WT, 17mut1 and 17mut1.1, respectively) and HphMX by a 2-step PCR with #5638/5639 and #5638/5640. These gene fragments were transformed to W303, resulting in yYC64 (WT), yYC65 (17mut1), and yYC66 (17mut1.1), respectively.

To test the expression levels of Ups1 and Ups2 (Fig. 5 g,h, and Extended Data Fig. 9), a 2xHA tag was introduced immediately before the stop codons of endogenous chromosomal *UPS1* or *UPS2* genes. Gene fragments containing a 5' homology arm immediately before the stop codon of *UPS1* (or *UPS2*), DNA fragment encoding a 2xHA-tag, a nourseothricin resistance cassette, and a 3' homology arm downstream of *UPS1* (or *UPS2*) were amplified by a 2-step PCR (primers #5316/5317 and #5318/5286 for *UPS1*, #5319/5320 and #5321/5163 for *UPS2*) and introduced into W303 (for DNA sequence, see, UPS1-2xHA and UPS2-2xHA). Chromosomal integration was confirmed by PCR (primers: #5287/5288 for *UPS1* and #5164/5165 for *UPS2*) and immunoblotting.

For yeast growth assays with Ups1/2 depletion (Extended Data Fig. 9f), the chromosomal *UPS2* was first deleted from W303 by transformation of a PCR product amplified from pYTK79 (ref. [72]) as the template and using primers #5160/5161 (first round) and #5162/5163 (second round). Colonies were selected on a hygromycin-containing plate, resulting in yYC60 (*ups2Δ*). Then, chromosomal *UPS1* was deleted from W303 and yYC60 by transformation of a PCR product amplified from pYTK74 (ref. [72]) as template using primers #5283/5284 and #5285/5286, generating yYC59 (*ups1Δ*) and yYC61 (*ups1Δ/usp2Δ*), respectively. The deletion of *UPS2* and *UPS1* was confirmed by PCR (primers used: #5287/5288 for *UPS1* and #5164/5165 for *UPS2*).

Proteomics Analysis

Offline basic pH reversed-phase fractionation

The TMTpro-labeled sample was resuspended in 100 µl of 10 mM NH_4HCO_3, pH 8.0, and fractionated using basic pH reverse phase HPLC (ref. [73]). Briefly, samples were off-line fractionated over a 90 min run, into 96 fractions by high pH reverse-phase HPLC (Agilent LC1260) through an aeris peptide xb-c18 column (Phenomenex), with mobile phase A containing 5% acetonitrile and 10 mM NH_4HCO_3 in LC-MS grade H_2O, and mobile phase B containing 90% acetonitrile and 10 mM NH_4HCO_3 in LC-MS grade H_2O (both pH 8.0). The 96 resulting fractions were then pooled in a non-continuous manner[74] into 24 fractions (whole proteome) or 18 fractions (mitochondria enriched). Fractions were vacuum centrifuged to near dryness. Each consolidated fraction was desalted via StageTip, dried again via vacuum centrifugation, and reconstituted in 5% acetonitrile, 1% formic acid for LC-MS/MS processing.

Proteomics analysis using TMT

Mass spectrometry data were collected using an Orbitrap Eclipse Tribrid mass spectrometer coupled to an UltiMate 3000 RSLCnano system liquid chromatography (LC) pump. Peptides were separated on a 100 µm inner diameter microcapillary column packed in-house with ~40 cm of HALO Peptide ES-C18 resin (2.7 µm, 160 Å, Advanced Materials Technology, Wilmington, DE) with a gradient consisting of 5%–28% (0-72 min), 28-45.5% (72-93 min) (acetonitrile, 0.1% FA) over a total 105 min run at ~350 nl/min. For analysis, we loaded 1/10 of each fraction onto the column. Each analysis used the Multi-Notch MS^3-based TMT method[75], to reduce ion interference compared to MS^2 quantification[76], combined with the FAIMS Pro Interface[77] and combined with Real-Time Search analysis software(ref. [78,79]). The scan sequence began with an MS^1 spectrum (Orbitrap analysis; resolution 60,000 at 200 Th; mass range 375−1500 m/z; automatic gain control (AGC) target 4×10^5; maximum injection time 50 ms). Precursors for MS^2 analysis were selected using a cycle type of 1.25 sec/CV method (FAIMS CV=-40/-60/-80). MS^2 analysis consisted of collision-induced dissociation

(quadrupole ion trap analysis; Turbo scan rate; AGC 1.0×10^4; isolation window 0.5 Th; normalized collision energy (NCE) 35; maximum injection time 35 ms). Monoisotopic peak assignment was used, and previously interrogated precursors were excluded using a dynamic window (90 s ±10 ppm). Following the acquisition of each MS^2 spectrum, a synchronous-precursor-selection (SPS) API-MS^3 scan was collected on the top 10 most intense ions b or y-ions matched by the online search algorithm in the associated MS^2 spectrum[77,78]. MS^3 precursors were fragmented by high-energy collision-induced dissociation (HCD) and analyzed using the Orbitrap (NCE 65; AGC 2.5×10^5; maximum injection time 200 ms, resolution was 50,000 at 200 Th). The closeout was set at two peptides per protein per fraction so that MS^3s were no longer collected for proteins having two peptide-spectrum matches (PSMs) that passed the quality filters[78].

Data analysis

Mass spectra were converted to mzXML(ref. [80]) and processed using the open-source Comet search engine (2020.01 rev. 4) software pipeline (ref. [81]) with the S. cerevisiae entries from UniProt (strain ATCC 204508 / S288c downloaded on 2020-07-16), with contaminants and reverse decoy sequences appended. Searches were conducted using a 50 ppm precursor ion tolerance and a 0.6 Da product ion tolerance to maximize sensitivity, combined with Comet database searching and linear discriminant analysis (LDA) (ref. [82,83]). TMTpro tags on lysine residues and peptide N-termini (+304.207 Da), along with carbamidomethylation of cysteines (+57.021 Da), were set as static modifications, while oxidation of methionine residues (+15.995 Da) was configured as a variable modification. Peptide-spectrum matches (PSMs) were adjusted to a 1% false discovery rate (FDR), with filtering via LDA to further refine the dataset, resulting in a final protein-level FDR of 1% (ref. [82,84,85]). Moreover, protein assembly was guided by principles of parsimony to produce the smallest set of proteins necessary to account for all observed peptides. For TMT-based reporter ion quantitation, the summed signal-to-noise (S:N) ratio for each TMT channel was identified, and the centroid that best matches the expected mass of the TMT reporter ion was determined, using an integration tolerance of 0.003 Da. Proteins were quantified by summing reporter ion counts across matching PSMs (ref. [86]). PSMs with a signal-to-noise ratio below 180 or isolation specificity below 50% for reporter ions were excluded from subsequent analysis. Reporter ion intensities were corrected based on the isotopic impurities of the TMT reagents, as specified by the manufacturer. For the whole proteome, signal-to-noise (S:N) measurements of proteins were summed and normalized so that the total signal across all proteins in each reporter ion channel was equal, correcting for unequal protein loading (i.e., column normalization). For the mito-enriched fractions, signal-to-noise (S:N) measurements of mitochondrial proteins only were summed and normalized so that the total signal across all proteins in each reporter ion channel was equal, correcting for unequal mito protein loading (i.e., mitochondria-specific column normalization). Finally, the abundance of each protein was expressed as a scaled value of the total, with the summed S/N ratio across all channels set to 180, thereby providing a relative abundance (RA) measurement.

Protein quantification values were exported for further analysis in Microsoft Excel, Prism, R in RStudio, and Perseus (ref. [87]).

Additional References

72. Lee, M. E., DeLoache, W. C., Cervantes, B. & Dueber, J. E. A Highly Characterized Yeast Toolkit for Modular, Multipart Assembly. *ACS Synth. Biol.* **4**, 975–986 (2015).
73. Wang, Y. *et al.* Reversed-phase chromatography with multiple fraction concatenation strategy for proteome profiling of human MCF10A cells. *PROTEOMICS* **11**, 2019–2026 (2011).
74. Paulo, J. A. *et al.* Quantitative mass spectrometry-based multiplexing compares the abundance of 5000 *S. cerevisiae* proteins across 10 carbon sources. *J. Proteomics* **148**, 85–93 (2016).
75. McAlister, G. C. *et al.* MultiNotch MS3 Enables Accurate, Sensitive, and Multiplexed Detection of Differential Expression across Cancer Cell Line Proteomes. *Anal. Chem.* **86**, 7150–7158 (2014).
76. Paulo, J. A., O'Connell, J. D. & Gygi, S. P. A Triple Knockout (TKO) Proteomics Standard for Diagnosing Ion Interference in Isobaric Labeling Experiments. *J. Am. Soc. Mass Spectrom.* **27**, 1620–1625 (2016).
77. Schweppe, D. K. *et al.* Characterization and Optimization of Multiplexed Quantitative Analyses Using High-Field Asymmetric-Waveform Ion Mobility Mass Spectrometry. *Anal. Chem.* **91**, 4010–4016 (2019).
78. Schweppe, D. K. *et al.* Full-Featured, Real-Time Database Searching Platform Enables Fast and Accurate Multiplexed Quantitative Proteomics. *J. Proteome Res.* **19**, 2026–2034 (2020).
79. Erickson, B. K. *et al.* Active Instrument Engagement Combined with a Real-Time Database Search for Improved Performance of Sample Multiplexing Workflows. *J. Proteome Res.* **18**, 1299–1306 (2019).
80. Rad, R. *et al.* Improved Monoisotopic Mass Estimation for Deeper Proteome Coverage. *J. Proteome Res.* **20**, 591–598 (2021).
81. Eng, J. K., Jahan, T. A. & Hoopmann, M. R. Comet: An open-source MS/MS sequence database search tool. *PROTEOMICS* **13**, 22–24 (2013).
82. Huttlin, E. L. *et al.* A Tissue-Specific Atlas of Mouse Protein Phosphorylation and Expression. *Cell* **143**, 1174–1189 (2010).
83. Beausoleil, S. A., Villén, J., Gerber, S. A., Rush, J. & Gygi, S. P. A probability-based approach for high-throughput protein phosphorylation analysis and site localization. *Nat. Biotechnol.* **24**, 1285–1292 (2006).
84. Savitski, M. M., Wilhelm, M., Hahne, H., Kuster, B. & Bantscheff, M. A Scalable Approach for Protein False Discovery Rate Estimation in Large Proteomic Data Sets [S]. *Mol. Cell. Proteomics* **14**, 2394–2404 (2015).
85. Elias, J. E. & Gygi, S. P. Target-Decoy Search Strategy for Mass Spectrometry-Based Proteomics. *Methods Mol. Biol. Clifton NJ* **604**, 55–71 (2010).
86. McAlister, G. C. *et al.* Increasing the Multiplexing Capacity of TMTs Using Reporter Ion Isotopologues with Isobaric Masses. *Anal. Chem.* **84**, 7469–7478 (2012).
87. Tyanova, S. *et al.* The Perseus computational platform for comprehensive analysis of (prote)omics data. *Nat. Methods* **13**, 731–740 (2016).

Supplementary Table 1. Cryo-EM data collection, refinement and validation statistics

	TIM23-stendomycin complex (PDB:9N84, EMDB: 49111)
**Data collection and processing**	
Magnification	81,000x
Voltage (kV)	300
Electron exposure (e^{-}/Å^2)	50
Defocus range (μm)	−0.8 to −1.6
Pixel size (Å)	1.05
Symmetry imposed	*C*1
Initial particle images (no.)	523,352
Final particle images (no.)	105,256
Map resolution (Å)	3.2
FSC threshold	0.143
Map resolution range (Å)	2.74 (highest), 7.49 (75% percentile)
**Refinement**	
Initial model used	PDB 8SCX
Model resolution (Å)	3.3
FSC threshold	0.5
Map sharpening *B* factor (Å^2)	83
Model composition	
Non-hydrogen atoms	5,854
Protein residues	760
Ligands	3
B factors (Å^2)	
Protein	69.03
Ligand	67.46
R.m.s. deviations	
Bond lengths (Å)	0.003
Bond angles (°)	0.530
**Validation**	
MolProbity score	1.39
Clashscore	7.10
Poor rotamers (%)	0
Ramachandran plot	
Favored (%)	98.13
Allowed (%)	1.87
Disallowed (%)	0
CaBLAM outliers (%)	1.09

Supplementary Table 2. List of yeast strains used in this study

Name	Genotype/Description	Reference
yMLT62	*MAT***a** *leu2-0*::*pACT1-GEV*::*HIS3, rps9Δ, mek1Δ, his3-1, met15-0, ura3-0*	A gift from J. Thorner
W303-1A	*MAT***a** *leu*2-3/112, *ura3*-1, *trp*1-1, *his3*-11/15, *ade*2-1, *can*1-100	A gift from J. Thorner
ySS150	W303-1A *URA3*::P_{CMV}*-tTA*	Sim et al. 2023
ySS055	yMLT62 *ura3-0*::pSS077(Tim23/17-Spot/44[–GS])::*URA3* *HO*::pSS082(Pam16/18)::*LEU2*	Sim et al. 2023
yYC17b	ySS150 P_{TIM17}*::KanMX-tetO7-*P_{CYC1}	Sim et al. 2023
yYC22	ySS150 P_{TIM22}*::KanMX-tetO7-*P_{CYC1}	This study
yYC51	yYC22 *TIM17(G20D)*	This study
yYC52	W303 *TIM17(G20D)*	This study
yYC53	yYC52 *TIM22(WT)-2xHA::NatMX*	This study
yYC54	yYC52 *TIM22(T52G)-2xHA::NatMX*	This study
yYC55	yYC52 *TIM22(22mut1)-2xHA::NatMX*	This study
yYC56	yYC52 *TIM22(22mut2)-2xHA::NatMX*	This study
yYC57	W303 *UPS1-2xHA::NatMX*	This study
yYC58	W303 *UPS2-2xHA::NatMX*	This study
yYC59	W303 *ups1Δ::URA3*	This study
yYC60	W303 *ups2Δ::HphMX*	This study
yYC61	yYC60 *ups1Δ::URA3*	This study
yYC64	yYC17b *LEU2::pTIM17-Tim17(WT)-2xSPOT::HphMX*	This study
yYC65	yYC17b *LEU2::pTIM17-Tim17(17mut1)-2xSPOT::HphMX*	This study
yYC66	yYC17b *LEU2::pTIM17-Tim17(17mut1.1)-2xSPOT::HphMX*	This study
yKW936	yMLT62 *TIM17-2xSPOT::HphMX HO::*P_{ALD6}*::2xHA-Mgr2::KanMX*	This study
yKW937	yMLT62 *TIM17-2xMyc::HphMX HO::*P_{ALD6}*::2xHA-Mgr2::KanMX*	This study
yKW976	yYC52 *HO::*P_{HHF1}*-TIM17(wt)-2xHA::LEU2*	This study
yKW977	yYC52 *HO::*P_{HHF1}*-TIM17(G20D)-2xHA::LEU2*	This study

Supplementary Table 3. List of plasmids

Name	Description	Reference
pYTK-e102	*LEU2* integration vector containing a *HphMX* marker (made from YTK parts)	This study
pYTK-e104	*HO* integration vector containing a *HphMX* marker (made from YTK parts)	This study
pYTK-e106	*HO* integration vector containing a *LEU2* marker (made from YTK parts)	This study
pYTK-e115	CEN/ARS plasmid containing a *NatMX* marker	This study
pYC002	pET32a Cyb2MTS-Cyb2Δ-DHFR	Sim et al. 2023
pYC101	pET32a Grx5MTS-Cyb2Δ-DHFR	This study
pYC102	pET32a F1bMTS-Cyb2Δ-DHFR	This study
pYC007	pYTK-e112 *pGAL1*-Grx5-S80-sfGFP-2xTEV-ALFA	Sim et al. 2023
pYC17a	pYTK-e115 *pTIM17*-Tim17-2xHA	Sim et al. 2023
pYC22	pYTK-e115 *pTIM22*-Tim22-2xHA	This study
pYTK74	YTK vector for genomic knockout containing a URA3 marker	Lee et al. 2015
pYTK79	YTK vector for genomic knockout containing a HphMX marker	Lee et al. 2015
pYC105	pYTK-e102-pTIM17-Tim17-2xSPOT	This study
pYC106	pYTK-e115 *pTIM17*-Tim17(17mut1)-2xHA	This study
pYC107	pYTK-e115 *pTIM17*-Tim17(17mut2)-2xHA	This study
pYC108	pYTK-e115 *pTIM17*-Tim17(17mut1.1)-2xHA	This study
pYC109	pYTK-e115 *pTIM22*-Tim22(22mut1)-2xHA	This study
pYC110	pYTK-e115 *pTIM22*-Tim22(22mut2)-2xHA	This study
pYC111	pYTK-e115 *pTIM22*-Tim22(T52G)-2xHA	This study
pSS254	pYTK-e105 *pMGR2*-Strep-Mgr2-tMGR2	Sim et al. 2023
pYC112	pYTK-e105 *pMGR2*-2xHA-Mgr2-tMGR2	This study
pSS122	pYTK-e112 *pTIM17*-Tim17-Spot	Sim et al. 2023
pYC113	pYTK-e102 *pTIM17*-Tim17-Spot	This study
pKW860	pYTK-e106 *pHHF1*-Tim17-2xHA (*pHHF1* originates from YTK015)	This study
pKW861	pYTK-e106 *pHHF1*-Tim17G20D-2xHA (*pHHF1* originates from YTK015)	This study

Supplementary Table 4. List of primers

Name	Sequence (including notes)
YC_3305	ctaaacgggatagtagagatagctgccaaataagatcaGGCGTTAGTATCGAATCG<br>(To replace the endogenous *TIM22* promoter with the TRE promoter. Uppercase for sequence specific to TRE-kanMX and lower case for sequences homologous to yeast chromosomal sequences directly before the starting codon of Tim22. Step1)
YC_3306	ctggacttatctgctccaaaccaaatcctgtgtacaccatGGATCCCCCGAATTG<br>(To replace the endogenous *TIM22* promoter with the TRE promoter. Uppercase for sequence specific to TRE-kanMX and lower case for sequences homologous to the N-terminus of Tim22. Step1)
YC_3307	ctatttttctgtcactactaaacgggatagtaCTAAACGGGATAGTAGAG<br>(To extend the homology arm.)
YC_3308	gagttagttcattataaggtttcttttgagCTGGACTTATCTGCTCC<br>(To extend the homology arm)
YC_3309	GACGATGCATCAGTC<br>(To confirm chromosomal integration of the TRE cassette into the *TIM22* locus)
YC_3310	CAGGCCTAAAACACC<br>(To confirm chromosomal integration of the TRE cassette into the *TIM22* locus)
SS_1702	ACTGCTATTGTTCAACAAAG<br>(To introduce G20D mutation to endogenous TIM17)
YC_2955	CGTGATCGAACTGTTC<br>(To introduce G20D mutation to endogenous TIM17)
YC_3311	CAATATGAGCGGTTACG<br>(Amplify TIM22 with endo promoter from gDNA, insert into pYC17a right before HA-tags by EcoRI/SpeI)
YC_3312	gaaaactagtTTCTTTAAAATCGTTTTGAGG<br>(Amplify TIM22 with endo promoter from gDNA, insert into pYC17a right before HA-tags by EcoRI/SpeI)
YC_5043	GTTCCAGATTACGCTTAG<br>(To introduce TIM17 mutants 17mut1 and 17mut2 into pYC17a by Gibson assembly)
YC_5044	CGAATGATCGGCTGA<br>(To introduce TIM17 mutants 17mut1 and 17mut2 into pYC17a by Gibson assembly)
YC_5045	ATGTCAGCCGATCATTC<br>(To introduce TIM17 mutants 17mut1 and 17mut2 into pYC17a by Gibson assembly)
YC_5046	CTAAGCGTAATCTGGAAC<br>(To introduce TIM17 mutants 17mut1 and 17mut2 into pYC17a by Gibson assembly)
YC_3311	CAATATGAGCGGTTACG<br>(To clone TIM22 into pYC17a with EcoRI/SpeI)
YC_3312	gaaaactagtTTCTTTAAAATCGTTTTGAGG<br>(To clone TIM22 into pYC17a with EcoRI/SpeI)
YC_4764	AATTCCTAACGTTGCTCC<br>(To replace endogenous TIM22 with TIM22-2xHA[WT, T52G, 22mut1, or 22mut2] and NatMX)
YC_4765	ctcccagggaattttttgctttactagtaacagtagttctgCAGTATAGCGACCAGC<br>(To replace endogenous TIM22 with TIM22-2xHA[WT, T52G, 22mut1, or 22mut2] and NatMX)
YC_4766	aaaaatgatcggcccagagtctagttaaagcatgattaattCTCCCAGGGAATTTTTTG<br>(To replace endogenous TIM22 with TIM22-2xHA[WT, T52G, 22mut1, or 22mut2] and NatMX)
YC_3890	gctaagactcttcttcgttaccaaaatcggatgtatttgagcTCTACCGTTCCAAAATC<br>(To replace Cyb2-MTS with Grx5-MTS by reverse PCR)
YC_3891	ccggaggatgggggaaaatgaccttatgggattgaattttgggagaaaCATATGTATATCTCCTTCTTAAAG<br>(To replace Cyb2-MTS with Grx5-MTS by reverse PCR)
YC_3798	gctccgcttctatccacttcgtggaaaagatgtatggcctcaTCTACCGTTCCAAAATC<br>(To replace Cyb2-MTS with F1b-MTS by reverse PCR)
YC_3799	ggattgtttggctgctttaaaagcagcacgggatgtagcagtatatagtcttggcaaaacCATATGTATATCTCCTTCTTAAAG<br>(To replace Cyb2-MTS with F1b-MTS by reverse PCR)

YC_5316	ggcatttgttatccaaaaactcgaagaggcgagaaatcctcagtttGGGGGCGGTTCT<br>(To add 2xHA tag to the C-terminus of UPS1, step1)
YC_5317	ttcgctcgcccatggtgatatctttaaagatctttaaatgCGGCGTTAGTATCGAATC<br>(To add 2xHA tag to the C- terminus of UPS1, step1)
YC_5318	aacgttaagaaaagcagaatgggcatGGCATTTGTTATCCAAAAAC<br>(To add 2xHA tag to the C- terminus of UPS1, step2)
YC_5286	ggacgtgaccaaatgcgagctgcactagttttctcaatTTCGCTCGCCCATG<br>(To add 2xHA tag to the C- terminus of UPS1, step2)
YC_5319	cagcaaaatattgacttgtttagagacgcatacaaccacgaaaatGGGGGCGGTTCT<br>(To add 2xHA tag to the C- terminus of UPS2, step1)
YC_5320	gaatagatagacagtagaggaatataagtagtatgcagtgCGGCGTTAGTATCGAATC<br>(To add 2xHA tag to the C- terminus of UPS2, step1)
YC_5321	actgaaaggagcggcagaaccatcttaCAGCAAAATATTGACTTGT<br>(To add 2xHA tag to the C- terminus of UPS2, step2)
YC_5163	cattatttacttttctatttacaacaaaagaacaaatGAATAGATAGACAGTAGAGG<br>(To add 2xHA tag to the C- terminus of UPS2, step2; To deplete UPS2, step2)
YC_5160	aagcatcaataggacgaaagaatagatttcagactaagatCTGTGGATAACCGTAGTCG<br>(To deplete UPS2, step1)
YC_5161	gaatagatagacagtagaggaatataagtagtatgcagtgGGGCGTTTTTTATTGGTC<br>(To deplete UPS2, step1)
YC_5162	gataaaattatcatgcgaagtgatgtacatatttttgaatttAAGCATCAATAGGACG<br>(To deplete UPS2, step2)
YC_5164	CGTTGATTGGTCAGAG<br>(To confirm the depletion of UPS2)
YC_5165	CCGTCTACAAGGTTAAC<br>(To confirm the depletion of UPS2)
YC_5283	ttctgagacggcggtaagatatccttaagagttgcaCTGTGGATAACCGTAGTCG<br>(To deplete UPS1, step1)
YC_5284	ttcgctcgcccatggtgatatctttaaagatctttaaatgGGGCGTTTTTTATTGGTC<br>(To deplete UPS1, step1)
YC_5285	gacagatggatgagtaatcatattcaataagtattgtctggcTTCTGAGACGGCGG<br>(To deplete UPS1, step2)
YC_5286	ggacgtgaccaaatgcgagctgcactagttttctcaatTTCGCTCGCCCATG<br>(To deplete UPS1, step2)
YC_5287	GTATTTCTCGCTCTCCC<br>(To confirm chromosomal deletion of *UPS1*)
YC_5288	TTGTTGAACCAGCGC<br>(To confirm chromosomal deletion of *UPS1*)
YC_2835	GCGTTATGGGTACCTCAGC<br>(For Error-prone PCR)
YC_2836	ATAGGGATAACTAGTAGCTTGCAGAG<br>(For Error-prone PCR)

Supplementary Table 5. DNA sequences

(Note, sequences are inserts only; the plasmid backbones are not included.)

Name	DNA sequence	Reference
TIM17(G20D)<br>(PCR product<br>to introduce<br>G20D<br>mutantion to<br>endogenose<br>TIM17)	ACTGCTATTGTTCAACAAAGTGGGTAACCCGGCATTCTTGCGCCAAAGTTTCCATTTTTATGCC<br>AAATCCAGAGGATAAAAGCACTATTCTCATCAAAAGATGGAAAAGCTGTGAAAGAGTCAAGTTC<br>AGAAGAAAGCGTTAAGAAAAATAGAGTACACGGGAGCGTT**atg**tcagccgatcattcgagagatccatgtccta<br>tagtcatactaaatgatttcggtgatgcttttgccatgggtgccattggtggtgttgtttggcatgggattaaaggttttagaaattcgccattaggtg<br>agcgtggttcaggagctatgagcgccattaaagcgcgtgctcccgtactgggtggtaattttggtgtgtggggtggtttattttcgacttttgattg<br>cgctgtgaaggccgttagaaagagagaggacccatggaatgctatcattgcagggtttttcactggtggcgctttagctgtaagaggtggttg<br>gaggcatacaaggaacagttcgatcacg<br>(Uppercase, part of TIM17 promoter; bold, start codon of TIM17; underlined, G20D mutation)	This study
TIM22<br>(PCR product<br>to be cloned<br>into pYC17a by<br>EcoRI/SpeI)	CAATATGAGCGGTTACGTTTTTACAAGGACACCTTTACATTATGAATTCCTAACGTTGCTCCAAT<br>GACGCTGCAATAGCTTATGTTTATCTGCAACGTACTTTTAATAGTTATTCCTTCTTGAATCTCTA<br>TAAGTATATCTATACCGGGTGACAATTTGATATTTTCGTACGCCGTTATGTGTTGGTTTTTGGTC<br>TTACTGATAAAATGGCATCGAGCTTACAACTGAAGAGAAAGACGATGCATCAGTCAACTTAATT<br>CTCTAATAAAACAGACAGCATTTCCTCGGATCCATTTCGTACATATAAAATCCGAAGCTTTTCAG<br>GACAAAATACTATTTTTCTGTCACTACTAAACGGGATAGTACTAAACGGGATAGTAGAGATAGC<br>TGCCAAATAAGATCAAGAAATTGTGATTTTAAATACTTTATACGAAG**atg**gtgtacacaggatttggtttggag<br>cagataagtccagctcaaaagaaaccttataatgaactaactccggaagaacagggtgaacggggtgctgaaatgatcatgaacttcatg<br>acttcctgtcctggaaaatcagttgtgagtggtgtaacagggtttgcactgggtggtgttttaggcctgttcatggcgtcaatggcatatgataca<br>cctcttcatacacctacacctgcaaatacagcagcaactgcaactgcgggaaatataggtgtgggtggaatatcaagaacagttcagcaa<br>atatctgatttacctttccggcagcaaatgaaacttcaatttaccgatatgggtaaaaaatcctactctagtgctaagaattttggctacattggta<br>tgatatatgctggggtggagtgtgtcatagagtcacttagagctaaaaatgatatttacaacggagtaaccgcagggtttttaccggtgctgg<br>gctggcatacaaagctggaccacaagctgccttaatgggtggtgccgggtttgctgccttttcagctgcaattgatttgtatatgaaaagtgag<br>gatggtagaccacctcaaaacgattttaaagaaACTAGTTTTC<br>(Lowercase, CDS of TIM22; bold, start codon of TIM22; underlined, EcoRI and SpeI sites)	This study
17mut1<br>(PCR product<br>to be cloned<br>into pYC17a)	**atg**tcagccgatcattcgagagatccatgtcctatagtcatactaaatgatttcggtggtgcttttgccTTGggtgccattggtggtgttgtttgg<br>catgggattaaaggttttagaaattcgccattaggtgagcgtggttcaggagctatgagcgccattaaagcgcgtgctcccgtactgggtggt<br>aattttggtgtgATTggtggtttaTATtcgacttttgattgcgctgtgaaggccgttagaaagagagaggacccatggaatgctatcattgca<br>gggtttttcactggtggcgctttagctgtaagaggtggttggaggcatacaaggaacagttcgatcacgTTTgcttgtTTTTCTggtgtga<br>ttgaaggtgtgATGctaatgtttcaaagatatgctgcttggcaagccaaacctatggctcctcctttgcccgaagcaccttcctctcaacctct<br>gcaagctactagt*tatccctatgacgtcccggactatgcaggatcctacccatacgatgttccagattacgct***tag**<br>(Bold, start and stop codons of TIM17; uppercase, mutants; italic, 2xHA tag)	This study
17mut1.1<br>(PCR product<br>to be cloned<br>into pYC17a)	**atg**tcagccgatcattcgagagatccatgtcctatagtcatactaaatgatttcggtACTgcttttgccTTGggtgccattggtggtgttgtttg<br>gcatgggattaaaggttttagaaattcgccattaggtgagcgtggttcaggagctatgagcgccattaaagcgcgtgctcccgtactgggtgg<br>taattttggtgtgATTggtggtttaTATtcgacttttgattgcgctgtgaaggccgttagaaagagagaggacccatggaatgctatcattgc<br>agggtttttcactggtggcgctttagctgtaagaggtggttggaggcatacaaggaacagttcgatcacgTTTgcttgtTTTTCTggtgtg<br>attgaaggtgtgATGctaatgtttcaaagatatgctgcttggcaagccaaacctatggctcctcctttgcccgaagcaccttcctctcaacctc<br>tgcaagctactagt*tatccctatgacgtcccggactatgcaggatcctacccatacgatgttccagattacgct***tag**<br>(Bold, start and stop codons of TIM17; uppercase, mutants; italic, 2xHA tag)	This study
17mut2<br>(PCR product<br>to be cloned<br>into pYC17a)	**atg**tcagccgatcattcgagagatccatgtcctatagtcatactaaatTCTttcggtACTgcttttgccTTGggtgccGTTTTGggtgtt<br>gtttggcatgggattaaaggttttagaaattcgccattaggtgagcgtggttcaggagctatgagcgccattaaagcgcgtgctcccgtaTC<br>TggtggtaattttggtgtgATTggtggtttaTATGCTacttttgattgcgctgtgaaggccgttagaaagagagaggacccatggaatgct<br>atcattgcagggtttttcactggtggcgctttagctgtaagaggtggttggaggcatacaaggaacagttcgatcacgTTTgcttgtTTTTC<br>TggtgtgattgaaggtTATATGctaatgtttcaaagatatgctgcttggcaagccaaacctatggctcctcctttgcccgaagcaccttcct<br>ctcaacctctgcaagctactagt*tatcccta*tgacgtcccggactatgcaggatcctacccatacgatgttccagattacgct**tag**<br>(Bold, start and stop codons of TIM17; uppercase, mutants; italic, 2xHA tag)	This study
22mut1<br>(Gene fragment<br>synthesized by<br>Twist)	caatccgccctcactacaaccggaattcctaacgttgctccaatgacgctgcaatagcttatgtttatctgcaacgtacttttaatagttattccttc<br>ttgaatctctataagtatatctataccgggtgacaatttgatattttcgtacgccgttatgtgttggtttttggtcttactgataaaatggcatcgagctt<br>acaactgaagagaaagacgatgcatcagtcaacttaattctctaataaaacagacagcatttcctcggatccatttcgtacatataaaatcc<br>gaagcttttcaggacaaaatactatttttctgtcactactaaacgggatagtactaaacgggatagtagagatagctgccaaataagatcaa<br>gaaattgtgattttaaatactttatacgaag**atg**gtgtacacaggatttggtttggagcagataagtccagctcaaaagaaaccttataatgaa<br>ctaactccggaagaacagggtgaacggggtgctgaaatgatcatgaacttcatgacttcctgtcctggaaaatcagttgtgagtggtgtaac<br>agggtttgcaATGggtggtgttttaggcctgttcatggcgtcaatggcatatgatacacctcttcatacacctacacctgcaaatacagcagc<br>aactgcaactgcgggaaatataggtgtgggtggaatatcaagaacagttcagcaaatatctgatttacctttccggcagcaaatgaaacttc<br>aatttaccgatatgggtaaaaaatcctactctagtgctaagaattttggctacTGGggtatgataTTTgctggggtggagtgtgtcatagag<br>tcacttagagctaaaaatgatatttacaacggagtaaccgcagggtttttaccggtgctgggctggcatacaaagctggaccacaagctgc<br>cttaatgggtggtgccgggTGTgctgccTTGTTAgctgcaattgatttgtatGGTaaaagtgaggatggtagaccacctcaaaacga<br>ttttaaagaaactagt*tatccctatgacgtcccggactatgcaggatcctatccatatgacgttccagattacgct***tga**atagccaatttcgacg<br>aacgatgaatgttttatatttgtaaatatcacctatcttaatattctgcacgaccgtcccagaatttaagaacgcttaataatttccagaactactgt<br>tactagtaaagcaaaaaaattccctgggagaattaatcatgctttaactagactctgggccgatcatttttgctggaagaaaatgggaagtgt<br>aaaaacgatacccttttccaattttcccatgtcgctgcagctactctggcgtcgatgaggga	This study

	(Bold, start and stop codons of TIM22; uppercase, mutants; italic, 2xHA tag; underlined EcoRI and PstI sites)	
22mut2<br>(Gene fragment synthesized by Twist)	caatccgccctcactacaaccggaattcctaacgttgctccaatgacgctgcaatagcttatgtttatctgcaacgtacttttaatagttattccttc<br>ttgaatctctataagtatatctataccgggtgacaatttgatattttcgtacgccgttatgtgttggtttttggtcttactgataaaatggcatcgagctt<br>acaactgaagagaaagacgatgcatcagtcaacttaattctctaataaaacagacagcatttcctcggatccatttcgtacatataaaatcc<br>gaagcttttcaggacaaaatactatttttctgtcactactaaacgggatagtactaaacgggatagtagagatagctgccaaataagatcaa<br>gaaattgtgattttaaatactttatacgaag**atg**gtgtacacaggatttggtttggagcagataagtccagctcaaaagaaaccttataatgaa<br>ctaactccggaagaacagggtgaacggggtgctgaaatgatcatgaacttcatgacttcctgtcctggaaaatcagttgtgAATggtgtaG<br>GTgggtttgcaATGggtggtATTGGTggcctgttcatggcgtcaatggcatatgatacacctcttcatacacctacacctgcaaataca<br>gcagcaactgcaactgcgggaaatataggtgtgggtggaatatcaagaacagttcagcaaatatctgatttacctttccggcagcaaatga<br>aacttcaatttaccgatatgggtaaaaaatcctactctTTGgctaagaattttggctacTGGggtatgataTTTTCTggggtggagtgtg<br>tcatagagtcacttagagctaaaaatgatatttacaacggagtaaccgcagggtttttttaccggtgctgggctggcatacaaagctggacca<br>caagctgccttaatgggtggtgccgggTGTgctgccTTGTTAgctgcaattgatttgGTTGGTaaaagtgaggatggtagaccac<br>ctcaaaacgattttaaagaa*actagttatccctatgacgtcccggactatgcaggatcctatccatatgacgttccagattacgct***tga**atagc<br>caatttcgacgaacgatgaatgttttatatttgtaaatatcacctatcttaatattctgcacgaccgtcccagaatttaagaacgcttaataatttcc<br>agaactactgttactagtaaagcaaaaaaattccctgggagaattaatcatgctttaactagactctgggccgatcatttttgctggaagaaa<br>atgggaagtgtaaaaacgatacccttttccaattttcccatgtcgctgcagctactctggcgtcgatgaggga<br>(Bold, start and stop codons of TIM22; uppercase, mutants; italic, 2xHA tag; underlined EcoRI and PstI sites)	This study
TIM17-2xSpot<br>(PCR product to introduce a Spot-tag to chromosomal *TIM17*)	GATATGCTGCTTGGCAAGCCAAACCTATGGCTCCTCCTTTGCCCGAAGCACCTTCCTCTCAAC<br>CTCTGCAAGCTgctagtggtaccctggaggtgttatttcagggcccgactgctagcggccctgatagagttagagcagtctcacattg<br>gtcttctggtggaggttctggcggtggttcaactccagatagagtacgtgctgtttctcactggagttcttgataaggcgcgccacttctaaataa<br>gcgaatttcttatgatttatgatttttattattaaataagttataaaaaaaataagtgtatacaaattttaaagtgactcttaggttttaaaacgaaaa<br>ttcttattcttgagtaactctttcctgtaggtcaggttgctttctcaggtatagtatgaggtcgctcttattgaccacacctctaccggcagatccgct<br>agggataacagggtaatatagatctgtttagcttgcctcgtccccgccgggtcacccggccagcgacatggaggcccagaataccctcctt<br>gacagtcttgacgtgcgcagctcaggggcatgatgtgactgtcgcccgtacatttagcccatacatccccatgtataatcatttgcatccatac<br>attttgatggccgcacggcgcgaagcaaaaattacggctcctcgctgcggacctgcgagcagggaaacgctcccctcacagacgcgttg<br>aattgtccccacgccgcgcccctgtagagaaatataaaaggttaggatttgccactgaggttcttctttcatatacttccttttaaaatcttgctag<br>gatacagttctcacatcacatccgaacataaacaacc**atg**ggtaaaaagcctgaactcaccgcgacgtctgtcgagaagtttctgatcgaa<br>aagttcgacagcgtctccgacctgatgcagctctcggagggcgaagaatctcgtgctttcagcttcgatgtaggagggcgtggatatgtcctg<br>cgggtaaatagctgcgccgatggtttctacaaagatcgttatgtttatcggcactttgcatcggccgcgctcccgattccggaagtgcttgacat<br>tggggaattcagcgagagcctgacctattgcatctcccgccgtgcacagggtgtcacgttgcaagacctgcctgaaaccgaactgcccgct<br>gttctgcagccggtcgcggaggccatggatgcgatcgctgcggccgatcttagccagacgagcgggttcggcccattcggaccgcaagg<br>aatcggtcaatacactacatggcgtgatttcatatgcgcgattgctgatccccatgtgtatcactggcaaactgtgatggacgacaccgtcagt<br>gcgtccgtcgcgcaggctctcgatgagctgatgctttgggccgaggactgccccgaagtccggcacctcgtgcacgcggatttcggctcca<br>acaatgtcctgacggacaatggccgcataacagcggtcattgactggagcgaggcgatgttcggggattcccaatacgaggtcgccaac<br>atcttcttctggaggccgtggttggcttgtatggagcagcagacgcgctacttcgagcggaggcatccggagcttgcaggatcgccgcggct<br>ccgggcgtatatgctccgcattggtcttgaccaactctatcagagcttggttgacggcaatttcgatgatgcagcttgggcgcagggtcgatgc<br>gacgcaatcgtccgatccggagccgggactgtcgggcgtacacaaatcgcccgcagaagcgcggccgtctggaccgatggctgtgtag<br>aagtactcgccgatagtggaaaccgacgccccagcactcgtccgagggcaaaggaa**taa**tcagtactgacaataaaaagattcttgtttt<br>caagaacttgtcatttgtatagtttttttatattgtagttgttctattttaatcaaatgttagcgtgatttatattttttttcgcctcgacatcatctgcccaga<br>tgcgaagttaagtgcgcagaaagtaatatcatgcgtcaatcgtatgtgaatgctggtcgctatactgctgtcgattcgatactaacgccgccat<br>ccagtgtcgaaaacgagctccaattcatcgatgatatcagatccactagtggcctatgcggGACGTATCGCACTAGCCTCCT<br>TTACGTTTTTACTTTATTTCAGCCTTTTATTTCAAGATTACCAACCATTTCTC<br>(Uppercase, homology arms; bold, start and stop codons of hygromycin B phosphotransferase)	This study
TIM17-2xMyc<br>(PCR product to introduce a Spot-tag to chromosomal *TIM17*)	GATATGCTGCTTGGCAAGCCAAACCTATGGCTCCTCCTTTGCCCGAAGCACCTTCCTCTCAAC<br>CTCTGCAAGCTgctagtggtaccctggaggtgttatttcagggcccgactgctagcggcgaacaaaagttgatttctgaagaagattt<br>gaacggtgaacaaaagctaatctccgaggaagacttgtgataaggcgcgccacttctaaataagcgaatttcttatgatttatgatttttattatt<br>aaataagttataaaaaaaataagtgtatacaaattttaaagtgactcttaggttttaaaacgaaaattcttattcttgagtaactctttcctgtaggt<br>caggttgctttctcaggtatagtatgaggtcgctcttattgaccacacctctaccggcagatccgctagggataacagggtaatatagatctgtt<br>tagcttgcctcgtccccgccgggtcacccggccagcgacatggaggcccagaataccctccttgacagtcttgacgtgcgcagctcaggg<br>gcatgatgtgactgtcgcccgtacatttagcccatacatccccatgtataatcatttgcatccatacattttgatggccgcacggcgcgaagca<br>aaaattacggctcctcgctgcggacctgcgagcagggaaacgctcccctcacagacgcgttgaattgtccccacgccgcgcccctgtaga<br>gaaatataaaaggttaggatttgccactgaggttcttctttcatatacttccttttaaaatcttgctaggatacagttctcacatcacatccgaacat<br>aaacaacc**atg**ggtaaaaagcctgaactcaccgcgacgtctgtcgagaagtttctgatcgaaaagttcgacagcgtctccgacctgatgc<br>agctctcggagggcgaagaatctcgtgctttcagcttcgatgtaggagggcgtggatatgtcctgcgggtaaatagctgcgccgatggtttct<br>acaaagatcgttatgtttatcggcactttgcatcggccgcgctcccgattccggaagtgcttgacattggggaattcagcgagagcctgacct<br>attgcatctcccgccgtgcacagggtgtcacgttgcaagacctgcctgaaaccgaactgcccgctgttctgcagccggtcgcggaggccat<br>ggatgcgatcgctgcggccgatcttagccagacgagcgggttcggcccattcggaccgcaaggaatcggtcaatacactacatggcgtg<br>atttcatatgcgcgattgctgatccccatgtgtatcactggcaaactgtgatggacgacaccgtcagtgcgtccgtcgcgcaggctctcgatg<br>agctgatgctttgggccgaggactgccccgaagtccggcacctcgtgcacgcggatttcggctccaacaatgtcctgacggacaatggcc<br>gcataacagcggtcattgactggagcgaggcgatgttcggggattcccaatacgaggtcgccaacatcttcttctggaggccgtggttggctt<br>gtatggagcagcagacgcgctacttcgagcggaggcatccggagcttgcaggatcgccgcggctccgggcgtatatgctccgcattggtc	This study

	ttgaccaactctatcagagcttggttgacggcaatttcgatgatgcagcttgggcgcagggtcgatgcgacgcaatcgtccgatccggagcc<br>gggactgtcgggcgtacacaaatcgcccgcagaagcgcggccgtctggaccgatggctgtgtagaagtactcgccgatagtggaaacc<br>gacgccccagcactcgtccgagggcaaaggaa**taa**tcagtactgacaataaaaagattcttgttttcaagaacttgtcatttgtatagtttttta<br>tattgtagttgttctattttaatcaaatgttagcgtgatttatattttttttcgcctcgacatcatctgcccagatgcgaagttaagtgcgcagaaagta<br>atatcatgcgtcaatcgtatgtgaatgctggtcgctatactgctgtcgattcgatactaacgccgccatccagtgtcgaaaacgagctccaatt<br>catcgatgatatcagatccactagtggcctatgcggGACGTATCGCACTAGCCTCCTTTACGTTTTTACTTTATTT<br>CAGCCTTTTATTTCAAGATTACCAACCATTTCTC<br>(Uppercase, homology arms; bold, start and stop codons of hygromycin B phosphotransferase)	
pALD6-2xHA-MGR2	TAAGGGCATGATAGAATTGGATTATGTAAAAGGTGAAGATACCATTGTAGAAGCAACCAGCAC<br>GTCGCCGTGGCTGATGAAGTCTCCTCTTGCCCGGGCCGCAGAAAAGAGGGGCAGTGGCCTG<br>TTTTTCGACATAAATGAGGGGCATGGCCAGCACCAAGACGTCATTGTTGCATATGGCGTATCC<br>AAGCCGAAACGGCGCTCGCCTCATCCCCACGGGAATAAGGCAGCCGACAAAAGAAAAACGAC<br>CGAAAAGGAACCAGAAAGAAAAAAGAGGGTGGGCGCGCCGCGGACGTGTAAAAAGATATGCA<br>TCCAGCTTCTATATCGCTTTAACTTTACCGTTTTGGGCATCGGGAACGTATGTAACATTGATCT<br>CCTCTTGGGAACGGTGAGTGCAACGAATGCGATATAGCACCGACCATGTGGGCAAATTCGTAA<br>TAAATTCGGGGTGAGGGGGATTCAAGACAAGCAACCTTGTTAGTCAGCTCAAACAGCGATTTA<br>ACGGTTGAGTAACACATCAAAACACCGTTCGAGGTCAAGCCTGGCGTGTTTAACAAGTTCTTG<br>ATATCATATATAAATGTAATAAGAAGTTTGGTAATATTCAATTCGAAGTGTTCAGTCTTTTACTTC<br>TCTTGTTTTATAGAAGAAAAAACATCAAGAAACATCTTTAACATACACAAACACATACTATCAGA<br>ATACAAGATCT**ATG***tatccctatgacgtcccggactatgcaggatcctatccatatgacgttccagattacgct*cctcctcttccacaa<br>aattatgcgcaacagcagccttcgaattgggacaaattcaaaatggggttgatgatgggtactaccgtcggtgtctgcacaggcatcctattt<br>ggtggatttgccatcgcaactcaaggcccaggtcctgatggtgtagttagaacactagggaaatacattgctggttcagcgggtacctttggg<br>ctatttatgtccatcgggtctataatcagaagtgatagtgaaagtagtccaatgtcccatcctaacctgaacctacagcaacaggcaagactg<br>gaaatgtggaagcttcgtgccaaatacggtatacgtaaggac**tga**GGGGGAAATTTTGCATATATTTACGCTTCCCC<br>ATCGTTTTCTTAAACATTGTTACTTATCTGCTTATTTTTATTTAAGATATCTATCTACCTGTTTATA<br>ACTCTCTCAATACCATGTATTTAAGTTCATCACGACGAGGCTTATTTACTTAATATATTTGTATAC<br>GTGGGAAATGTCTAATATGTATACCGGTTTGCATCTTATTTTGGGTGGGGTCACTAACGGCAAG<br>ACTCATAAAATAAAGTATTCGCTAAGCACTCTGTTATATTTGTAAACTTTGAGAAAGGAAATGGG<br>AACCAAAGCATATAATAGACAAT<br>(Uppercase, pALD6 and tMGR2; bold, start and stop codons of MGR2; italic, 2xHA tag)	This study
pYC113 (pYTK-e102 pTIM17-Tim17-Spot)	GAATTCctccagcattataaagcatatctaacaataccattcgggttatactgaatagcccacgcacgggttcggggttgtaacgattata<br>tgcatttcataacatgccactgtcagtggcgccacggaaggatagcgggactttatcaaatcattaatttctgtgcataaagtgtatagtatattc<br>atatagatatgtatatatatcattgaatacagaaatcttcgggtgtatacaacgaacattcatcggacgcctttttttactgctattgttcaacaaag<br>tgggtaacccggcattcttgcgccaaagtttccatttttatgccaaatccagaggataaaagcactattctcatcaaaagatggaaaagctgt<br>gaaagagtcaagttcagaagaaagcgttaagaaaaatagagtacacgggagcgtt**atg**tcagccgatcattcgagagatccatgtcctat<br>agtcatactaaatgatttcggtggtgcttttgccatgggtgccattggtggtgttgtttggcatgggattaaaggttttagaaattcgccattaggtg<br>agcgtggttcaggagctatgagcgccattaaagcgcgtgctcccgtactgggtggtaattttggtgtgtggggtggtttattttcgacttttgattg<br>cgctgtgaaggccgttagaaagagagaggacccatggaatgctatcattgcagggtttttcactggtggcgctttagctgtaagaggtggttg<br>gaggcatacaaggaacagttcgatcacgtgtgcttgtttgttgggtgtgattgaaggtgtgggactaatgtttcaaagatatgctgcttggcaag<br>ccaaacctatggctcctcctttgcccgaagcaccttcctctcaacctctgcaagctACTAGT*cctgatagagttagagcagtctcacattgg*<br>*tcttctggtggaggttctggcggtggttcaactccagatagagtacgtgctgtttctcactggagttct***tag**gcatgtagacattatatgagccatt<br>ttttcatcgttgttggaagtacccttattcggcatgtttttttgttacataaatgacgtatcgcactagcctcctttacgtttttactttatttcagccttttattt<br>caagattaccaaccatttctctcaaccatgtacatattatatattgaaaaagtaccatacttcatctctgaagagaaaatatcaacaagccaa<br>aagctagtgcagaaactggatccgcaCTGCAG<br>(Uppercase, *Eco*RI, *Spe*I, *Pst*I sites; bold, start and stop codons; italic, 2xSpot tag)	This study
pYC101 (pET32a Grx5MTS-Cyb2Δ-DHFR)	**ATG**TTTCTCCCAAAATTCAATCCCATAAGGTCATTTTCCCCCATCCTCCGGGCTAAGACTCTTC<br>TTCGTTACCAAAATCGGATGTATTTGAGCtctaccgttccaaaatccaagtcgttcgaacaagactcaagttccgtggcg<br>tatctgaactggcataatggccaaatcgacaacgagccgaaactggatatgaataaacaaaagatttcgcccgctgaagttgccaagcat<br>aacaagcccgatgattcgtgggttgtgatcaatggttacgtatacgacttaacgcgtttcctgccaaatcatccaggtgggcaggatgttatca<br>agtttaacgccgggaaagatgtcactgctatttttgaaccactgcacgctcctaatgtcatcgataagtatattgctcccgaaaaaaaaattggg<br>tcccctgcaaggatccggcggtaccgttcgtccattgaactgcatcgtcgccgtgtcccaaaatatggggattggcaagaacggtgaccttc<br>cctggcctccgctccgcaacgagttcaagtacttccaacgtatgaccacaacctcttcagtggaaggtaaacagaatctggtgattatgggt<br>cgcaaaacctggttctccattcctgagaagaatcgtcctttaaaggaccgtattaatattgttctcagtcgtgaactcaaagaaccaccacgtg<br>gagctcattttcttgccaaaagtttggatgatgccttacgtcttattgaacaaccggaattggcaagtaaagtagacatggtttggattgtcgga<br>ggcagttctgtttaccaggaagccatgaatcaaccaggccacctccgcctctttgtgacacgcatcatgcaggaatttgaaagtgacacgttt<br>ttcccagaaattgatttggggaaatataaacttctcccagaatacccaggcgtcctctctgaggtccaggaggaaaaaggcatcaagtata<br>agtttgaagtctacgagaagaaagacctcgag*caccaccaccaccaccac***tga**<br>(Uppercase, Grx5-MTS; bold, start and stop codons; italic, 6xHis tag)	This study
pYC102 (pET32a F1bMTS-Cyb2Δ-DHFR)	**ATG**GTTTTGCCAAGACTATATACTGCTACATCCCGTGCTGCTTTTAAAGCAGCCAAACAATCCG<br>CTCCGCTTCTATCCACTTCGTGGAAAAGATGTATGGCCTCAtctaccgttccaaaatccaagtcgttcgaacaa<br>gactcaagttccgtggcgtatctgaactggcataatggccaaatcgacaacgagccgaaactggatatgaataaacaaaagatttcgccc<br>gctgaagttgccaagcataacaagcccgatgattcgtgggttgtgatcaatggttacgtatacgacttaacgcgtttcctgccaaatcatccag<br>gtgggcaggatgttatcaagtttaacgccgggaaagatgtcactgctatttttgaaccactgcacgctcctaatgtcatcgataagtatattgct<br>cccgaaaaaaaaattgggtcccctgcaaggatccggcggtaccgttcgtccattgaactgcatcgtcgccgtgtcccaaaatatggggattg<br>gcaagaacggtgaccttccctggcctccgctccgcaacgagttcaagtacttccaacgtatgaccacaacctcttcagtggaaggtaaaca	

	gaatctggtgattatgggtcgcaaaacctggttctccattcctgagaagaatcgtcctttaaaggaccgtattaatattgttctcagtcgtgaact<br>caaagaaccaccacgtggagctcattttcttgccaaaagtttggatgatgccttacgtcttattgaacaaccggaattggcaagtaaagtaga<br>catggtttggattgtcggaggcagttctgtttaccaggaagccatgaatcaaccaggccacctccgcctctttgtgacacgcatcatgcagga<br>atttgaaagtgacacgtttttcccagaaattgatttggggaaatataaacttctcccagaatacccaggcgtcctctctgaggtccaggagga<br>aaaaggcatcaagtataagtttgaagtctacgagaagaaagacctcgag*caccaccaccaccaccac***tga**<br>(Uppercase, Grx5-MTS; bold, start and stop codons; italic, 6xHis tag)	
UPS1-HA<br>(PCR product to introduce 2xHA tag to the C-terminus of UPS1)	AACGTTAAGAAAAGCAGAATGGGCATGGCATTTGTTATCCAAAAACTCGAAGAGGCGAGAAAT<br>CCTCAGTTTgggggcggttcttatccctatgacgtcccggactatgcaggatcctatccatatgacgttccagattacgcttaaggcgcg<br>ccacttctaaataagcgaatttcttatgatttatgatttttattattaaataagttataaaaaaaaataagtgtatacaaattttaaagtgactcttaggt<br>tttaaaacgaaaattcttattcttgagtaactctttcctgtaggtcaggttgctttctcaggtatagtatgaggtcgctcttattgaccacacctctacc<br>ggcagatccgctagggataacagggtaatatagatctgtttagcttgcctcgtccccgccgggtcacccggccagcgacatggaggccca<br>gaataccctccttgacagtcttgacgtgcgcagctcaggggcatgatgtgactgtcgcccgtacatttagcccatacatccccatgtataatca<br>tttgcatccatacattttgatggccgcacggcgcgaagcaaaaattacggctcctcgctgcagacctgcgagcagggaaacgctcccctca<br>cagacgcgttgaattgtccccacgccgcgcccctgtagagaaatataaaaggttaggatttgccactgaggttcttctttcatatacttcctttta<br>aaatcttgctaggatacagttctcacatcacatccgaacataaacaacc**atg**ggtaccactcttgacgacacggcttaccggtaccgcacc<br>agtgtcccgggggacgccgaggccatcgaggcactggatgggtccttcaccaccgacaccgtcttccgcgtcaccgccaccggggacg<br>gcttcaccctgcgggaggtgccggtggacccgcccctgaccaaggtgttccccgacgacgaatcggacgacgaatcggacgacgggg<br>aggacggcgacccggactcccggacgttcgtcgcg**tac**ggggacgacggcgacctggcgggcttcgtggtcgtctcgtactccggctgg<br>aaccgccggctgaccgtcgaggacatcgaggtcgccccggagcaccgggggcacggggtcgggcgcgcgttgatggggctcgcgac<br>ggagttcgcccgcgagcggggcgccgggcacctctggctggaggtcaccaacgtcaacgcaccggcgatccacgcgtaccggcggat<br>ggggttcaccctctgcggcctggacaccgccctgtacgacggcaccgcctcggacggcgagcaggcgctctacatgagcatgccctgcc<br>cctaatcagtactgacaataaaaagattcttgttttcaagaacttgtcatttgtatagtttttttatattgtagttgttctattttaatcaaatgttagcgtg<br>atttatatttttttcgcctcgacatcatctgcccagatgcgaagttaagtgcgcagaaagtaatatcatgcgtcaatcgtatgtgaatgctggtcg<br>ctatactgctgtcgattcgatactaacgccgCATTTAAAGATCTTTAAAGATATCACCATGGGCGAGCGAAATT<br>GAGAAAACTAGTGCAGCTCGCATTTGGTCACGTCC<br>(Uppercase, homology arms; bold, start and stop codons of nourseothricin N-acetyl transferase)	This study
UPS2-HA<br>(PCR product to introduce 2xHA tag to the C-terminus of UPS2)	ACTGAAAGGAGCGGCAGAACCATCTTACAGCAAAATATTGACTTGTTTAGAGACGCATACAAC<br>CACGAAAATgggggcggttcttatccctatgacgtcccggactatgcaggatcctatccatatgacgttccagattacgcttaaggcgc<br>gccacttctaaataagcgaatttcttatgatttatgatttttattattaaataagttataaaaaaaaataagtgtatacaaattttaaagtgactcttag<br>gttttaaaacgaaaattcttattcttgagtaactctttcctgtaggtcaggttgctttctcaggtatagtatgaggtcgctcttattgaccacacctcta<br>ccggcagatccgctagggataacagggtaatatagatctgtttagcttgcctcgtccccgccgggtcacccggccagcgacatggaggcc<br>cagaataccctccttgacagtcttgacgtgcgcagctcaggggcatgatgtgactgtcgcccgtacatttagcccatacatccccatgtataat<br>catttgcatccatacattttgatggccgcacggcgcgaagcaaaaattacggctcctcgctgcagacctgcgagcagggaaacgctcccct<br>cacagacgcgttgaattgtccccacgccgcgcccctgtagagaaatataaaaggttaggatttgccactgaggttcttctttcatatacttccttt<br>taaaatcttgctaggatacagttctcacatcacatccgaacataaacaacc**atg**ggtaccactcttgacgacacggcttaccggtaccgca<br>ccagtgtcccgggggacgccgaggccatcgaggcactggatgggtccttcaccaccgacaccgtcttccgcgtcaccgccaccgggga<br>cggcttcaccctgcgggaggtgccggtggacccgcccctgaccaaggtgttccccgacgacgaatcggacgacgaatcggacgacgg<br>ggaggacggcgacccggactcccggacgttcgtcgcg**tac**ggggacgacggcgacctggcgggcttcgtggtcgtctcgtactccggct<br>ggaaccgccggctgaccgtcgaggacatcgaggtcgccccggagcaccgggggcacggggtcgggcgcgcgttgatggggctcgcg<br>acggagttcgcccgcgagcggggcgccgggcacctctggctggaggtcaccaacgtcaacgcaccggcgatccacgcgtaccggcgg<br>atggggttcaccctctgcggcctggacaccgccctgtacgacggcaccgcctcggacggcgagcaggcgctctacatgagcatgccctg<br>cccctaatcagtactgacaataaaaagattcttgttttcaagaacttgtcatttgtatagtttttttatattgtagttgttctattttaatcaaatgttagcg<br>tgatttatatttttttcgcctcgacatcatctgcccagatgcgaagttaagtgcgcagaaagtaatatcatgcgtcaatcgtatgtgaatgctggt<br>cgctatactgctgtcgattcgatactaacgccgCACTGCATACTACTTATATTCCTCTACTGTCTATCTATTCATT<br>TGTTCTTTTGTTGTAAATAGAAAAGTAAATAATG<br>(Uppercase, homology arms; bold, start and stop codons of nourseothricin N-acetyl transferase)	This study
