# Supplementary material for: Topogenic sequence recognition at TIM complexes revealed by a stendomycin-bound structure
Source: Nat Chem Biol. 2026 Aug 26;22(11):1863–74. doi: 10.1038/s41589-026-02304-z (PMC13710162; doi:10.1038/s41589-026-02304-z)
Supplement: Supplementary file 2 [file 41589_2026_2304_MOESM2_ESM.pdf]

nature portfolio

Corresponding author(s): Eunyong Park

Last updated by author(s): Jun 23, 2026

Reporting Summary

Nature Portfolio wishes to improve the reproducibility of the work that we publish. This form provides structure for consistency and transparency in reporting. For further information on Nature Portfolio policies, see our Editorial Policies and the Editorial Policy Checklist.

Statistics

For all statistical analyses, confirm that the following items are present in the figure legend, table legend, main text, or Methods section.

n/a	Confirmed	
☐	☒	The exact sample size (*n*) for each experimental group/condition, given as a discrete number and unit of measurement
☐	☒	A statement on whether measurements were taken from distinct samples or whether the same sample was measured repeatedly
☐	☒	The statistical test(s) used AND whether they are one- or two-sided<br>*Only common tests should be described solely by name; describe more complex techniques in the Methods section.*
☒	☐	A description of all covariates tested
☒	☐	A description of any assumptions or corrections, such as tests of normality and adjustment for multiple comparisons
☐	☒	A full description of the statistical parameters including central tendency (e.g. means) or other basic estimates (e.g. regression coefficient) AND variation (e.g. standard deviation) or associated estimates of uncertainty (e.g. confidence intervals)
☐	☒	For null hypothesis testing, the test statistic (e.g. *F*, *t*, *r*) with confidence intervals, effect sizes, degrees of freedom and *P* value noted<br>*Give P values as exact values whenever suitable.*
☒	☐	For Bayesian analysis, information on the choice of priors and Markov chain Monte Carlo settings
☒	☐	For hierarchical and complex designs, identification of the appropriate level for tests and full reporting of outcomes
☒	☐	Estimates of effect sizes (e.g. Cohen's *d*, Pearson's *r*), indicating how they were calculated

Our web collection on statistics for biologists contains articles on many of the points above.

Software and code

Policy information about availability of computer code

Data collection	Cryo-EM data was collected with SerialEM v4.0; mass spec data was collected on an Orbitrap Eclipse Tribrid mass spectrometer with software supplied by the manufacturer
Data analysis	For cryo-EM and structural analysis: Warp v1.0, cryoSPARC v4.0.2, Coot v0.9.8.1, Phenix v1.19, Grade2 web server v1.3.1rc1, MolProbity v4.5, Adaptive Poisson-Boltzmann Solver v3.0, PyMOL v2.5.4, Chimera v1.16, ChimeraX v1.5, AlphaFold2 v2.2.0. For mass spec: Comet search engine (2020.01 rev.4), Microsoft Excel, Prism, RStudio, Perseus.

For manuscripts utilizing custom algorithms or software that are central to the research but not yet described in published literature, software must be made available to editors and reviewers. We strongly encourage code deposition in a community repository (e.g. GitHub). See the Nature Portfolio guidelines for submitting code & software for further information.

Data

Policy information about availability of data

All manuscripts must include a data availability statement. This statement should provide the following information, where applicable:

- Accession codes, unique identifiers, or web links for publicly available datasets
- A description of any restrictions on data availability
- For clinical datasets or third party data, please ensure that the statement adheres to our policy

The cryo-EM map and atomic model of the stendomycin-bound TIM23 complex are available from the Electron Microscopy Data Bank (EMDB accession no.

EMD-49111) and Protein Data Bank (PDB accession ID. 9N84). The MS TMTpro proteomic data have been deposited in the MassIVE repository under the dataset identifier MSV000098464. The RNA-seq data have been deposited in the NCBI GEO repository under the accession ID GSE336173.

Research involving human participants, their data, or biological material

Policy information about studies with human participants or human data. See also policy information about sex, gender (identity/presentation), and sexual orientation and race, ethnicity and racism.

Reporting on sex and gender	N/A
Reporting on race, ethnicity, or other socially relevant groupings	N/A
Population characteristics	N/A
Recruitment	N/A
Ethics oversight	N/A

Note that full information on the approval of the study protocol must also be provided in the manuscript.

Field-specific reporting

Please select the one below that is the best fit for your research. If you are not sure, read the appropriate sections before making your selection.

☒ Life sciences ☐ Behavioural & social sciences ☐ Ecological, evolutionary & environmental sciences

For a reference copy of the document with all sections, see nature.com/documents/nr-reporting-summary-flat.pdf

Life sciences study design

All studies must disclose on these points even when the disclosure is negative.

Sample size	No statistical methods were used to predetermine sample size. For cryo-EM data, sample sizes were determined by the availability of microscope time. Within the confines of limited microscope time, data was collected until we were able to refine a high resolution 3D reconstruction map that enabled sufficiently accurate modeling of our structures. Yeast used in the growth assays were grown until log phase and then plated, following standard practices in the field. The growth assay contains millions of cells, and we are only looking at the growth of the population of a specific yeast strain. For biochemical experiments, the amount of proteins used was chosen based on commonly used sample sizes in the field.
Data exclusions	No data was systematically excluded. The process of generating 3D maps from cryo-EM particles involves sorting for particles that are damaged, have weak signal, or are unlikely to refine correctly. This is a standard practice in the field and was implemented using Warp and cryoSPARC.
Replication	There was no attempt to replicate cryo-EM results because sufficient data was collected in the initial trial and limited microscope time, as described above. Most biochemical experiments have been replicated with at least two independent experiments (some protein expression verification experiments were performed once). The number of replications for each experiment is stated in the figure legends.
Randomization	N/A
Blinding	N/A

Reporting for specific materials, systems and methods

We require information from authors about some types of materials, experimental systems and methods used in many studies. Here, indicate whether each material, system or method listed is relevant to your study. If you are not sure if a list item applies to your research, read the appropriate section before selecting a response.

Materials & experimental systems

n/a	Involved in the study
☐	☒ Antibodies
☒	☐ Eukaryotic cell lines
☒	☐ Palaeontology and archaeology
☒	☐ Animals and other organisms
☒	☐ Clinical data
☒	☐ Dual use research of concern
☒	☐ Plants

Methods

n/a	Involved in the study
☒	☐ ChIP-seq
☐	☒ Flow cytometry
☒	☐ MRI-based neuroimaging

Antibodies

Antibodies used	Mouse monoclonal anti-HA-tag (Invitrogen #26183, 1:5000), mouse monoclonal anti-His-tag (Invitrogen #MA1-21315, 1:1000), mouse monoclonal anti-GFP-tag (Roche #11814460001, 1:1000), rabbit monoclonal anti Spot-tag (homemade, 1:500), rabbit polyclonal anti-Pgk1, rabbit polyclonal anti-Tim17 (gift from C. Koehler, 1:250), rabbit polyclonal anti-Tim9 (gift from C. Koehler, 1:250), and rabbit polyclonal anti-Tom70 (gift from N. Pfanner, 1:250). Secondary antibodies conjugated to HRP used in this study were goat anti-rabbit (Thermo #31460, 1:10,000) and goat anti-mouse (Thermo #31430, 1:10,000).
Validation	The anti-HA, anti-His, and anti-GFP antibodies are validated by relative expression and immunoblotting, as described on their manufacturer's websites. The anti-Pgk1 antibody was described in Baum P, Thorner J, Honig L (1978) Proc. Natl. Acad. Sci. USA 75:4962-4966 and many other papers by J. Thorner. Anti-Tim17, Tim9, and Tom70 antibodies are previously used by many publications by the Koehler group and the Pfanner group, and we further validated them by immunoblotting analysis for molecular sizes and subcellular fractionation behaviors of the targets.

Plants

Seed stocks	N/A
Novel plant genotypes	N/A
Authentication	N/A

Flow Cytometry

Plots

Confirm that:

☒ The axis labels state the marker and fluorochrome used (e.g. CD4-FITC).

☒ The axis scales are clearly visible. Include numbers along axes only for bottom left plot of group (a 'group' is an analysis of identical markers).

☒ All plots are contour plots with outliers or pseudocolor plots.

☒ A numerical value for number of cells or percentage (with statistics) is provided.

Methodology

Sample preparation	Yeast strains were grown to mid-log phase, treated with stendomycin for indicated time. Equal number of cells were collected for each strain, washed with deionized water, and stained with 200 nM rhodamine 123 for 10min at room temperature before loaded to the instrument.
Instrument	Attune NxT Acoustic Focusing Cytometer (Invitrogen)
Software	FlowJo v10.10.0
Cell population abundance	About 1 millon yeast cells (0.05 OD) were collected for each measurement.

Gating strategy

Intact cells were selected by SSC-A vs FSC-A plots. Single cells were selected by FSC-H vs FSC-A plots.

☐ Tick this box to confirm that a figure exemplifying the gating strategy is provided in the Supplementary Information.
