# Supplementary material for: Topogenic sequence recognition at TIM complexes revealed by a stendomycin-bound structure
Source: Nat Chem Biol. 2026 Aug 26;22(11):1863–74. doi: 10.1038/s41589-026-02304-z (PMC13710162; doi:10.1038/s41589-026-02304-z)
Supplement: Supplementary file 3 [file 41589_2026_2304_MOESM4_ESM.pdf]

Fig. 2g

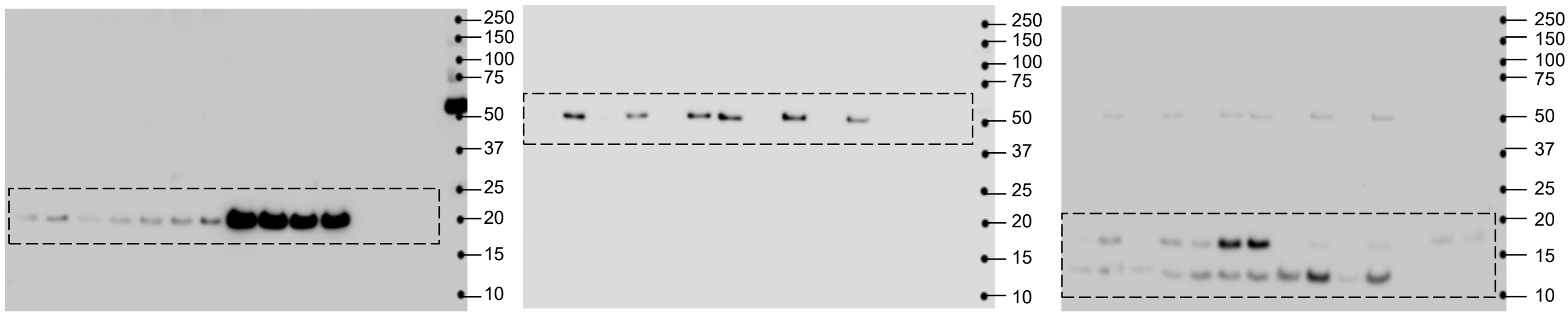

IP: α-SPOT-tag (Tim17)
IB: α-Tim17

IP: α-SPOT-tag (Tim17)
IB: α-GFP (substrate)

IP: α-SPOT-tag
IB: α-HA-tag (Mgr2)

All three blots are from the same membrane.

Fig. 3i

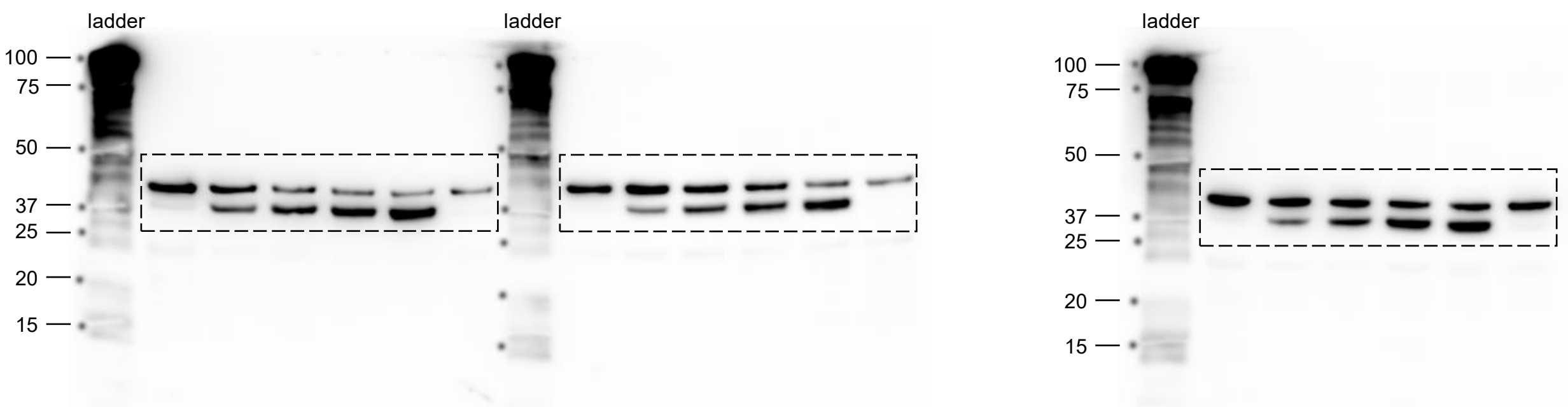

IB: α-His-tag (Left: WT Tim17; Right: 17mut1)

IB: α-His-tag (17mut1.1)

Fig. 5g

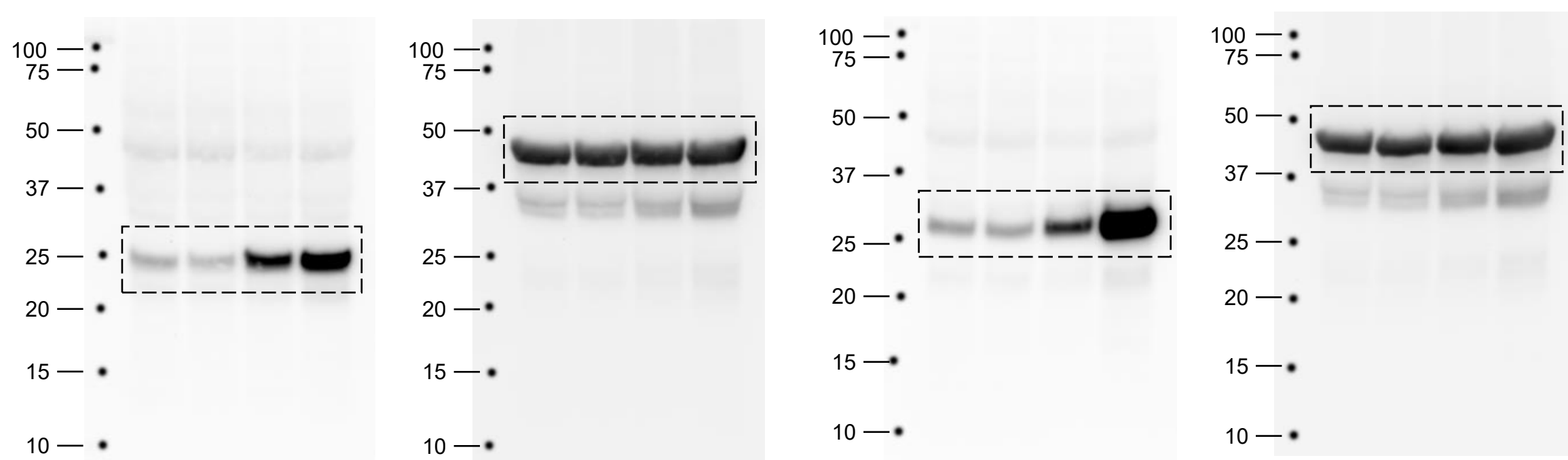

IB: α-HA-tag (Ups1)

IB: α-Pgk1

Both blots are from the same membrane.

IB: α-HA-tag (Ups2)

IB: α-Pgk1

Both blots are from the same membrane.

Fig. 5h

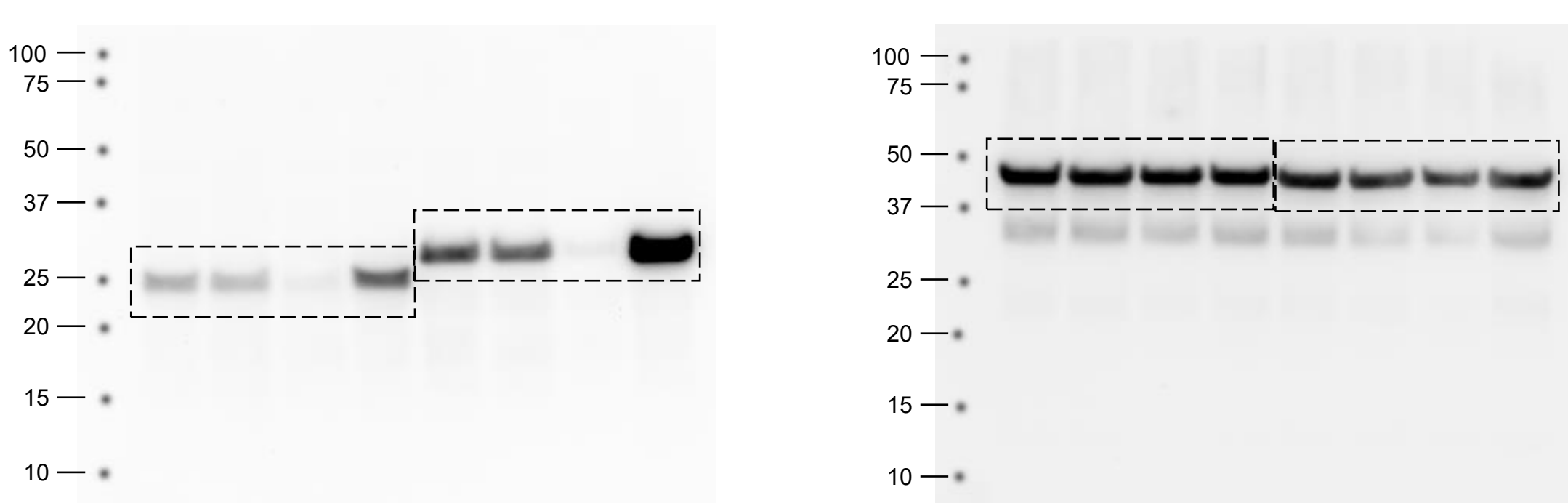

IB: α-HA-tag
Left: Ups1(exposure was enhanced for Fig. 5h top panel)
Right: Ups2

IB: α-Pgk1
Left: for Ups1
Right: for Ups2

Both blots are from the same membrane.

Extended Data Fig. 1b

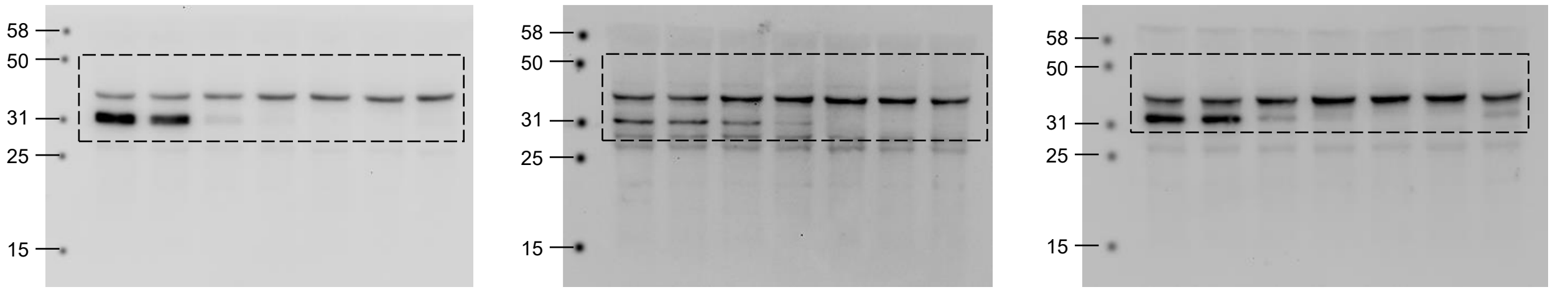

IB: α-His-tag (Cyb2-MTS)

IB: α-His-tag (Grx5-MTS)

IB: α-His-tag (F1β-MTS)

Extended Data Fig. 1d

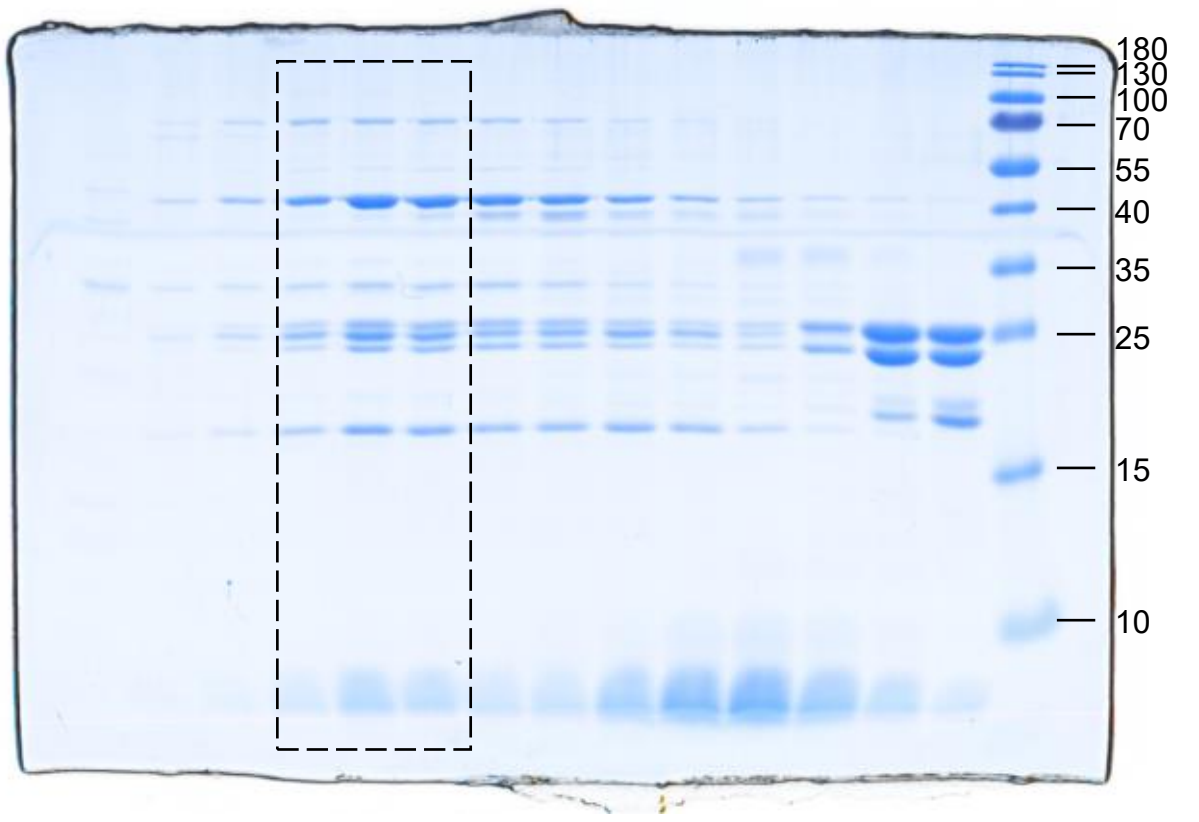

Extended Data Fig. 3c

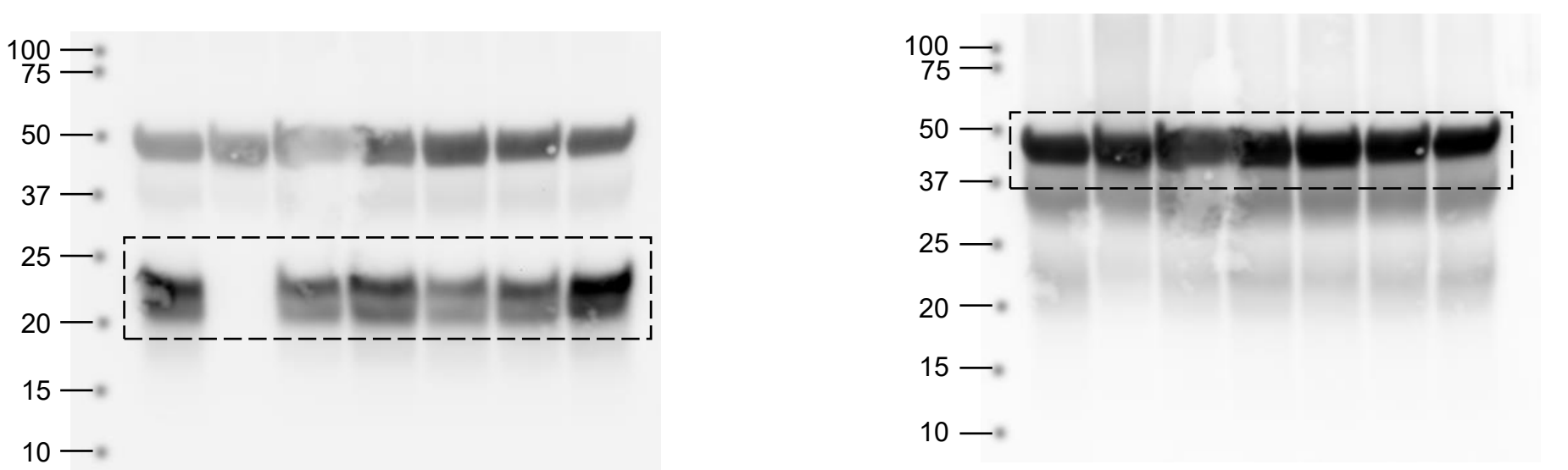

IB: α-HA-tag (Tim17)

IB: α-Pgk1

Both blots are from the same membrane.

Extended Data Fig. 3j

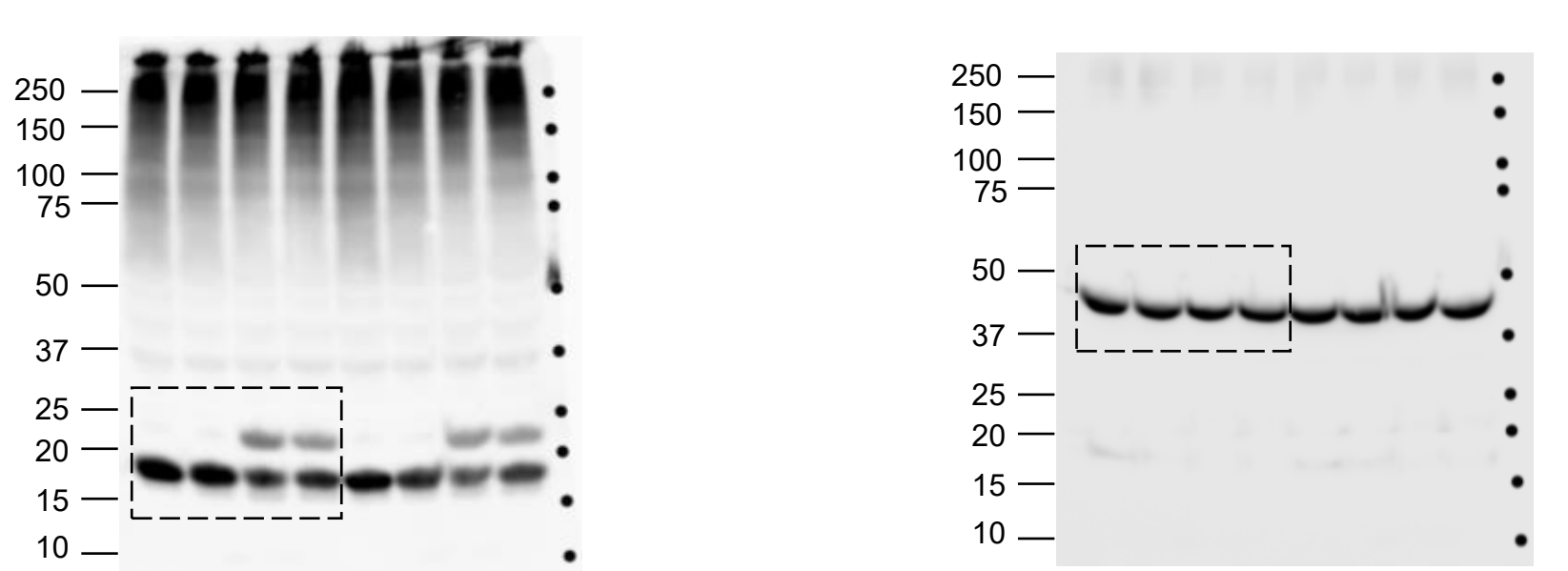

IB: α-HA-tag (Tim17)

IB: Pgk1

Both blots are from the same membrane.

Extended Data Fig. 4b

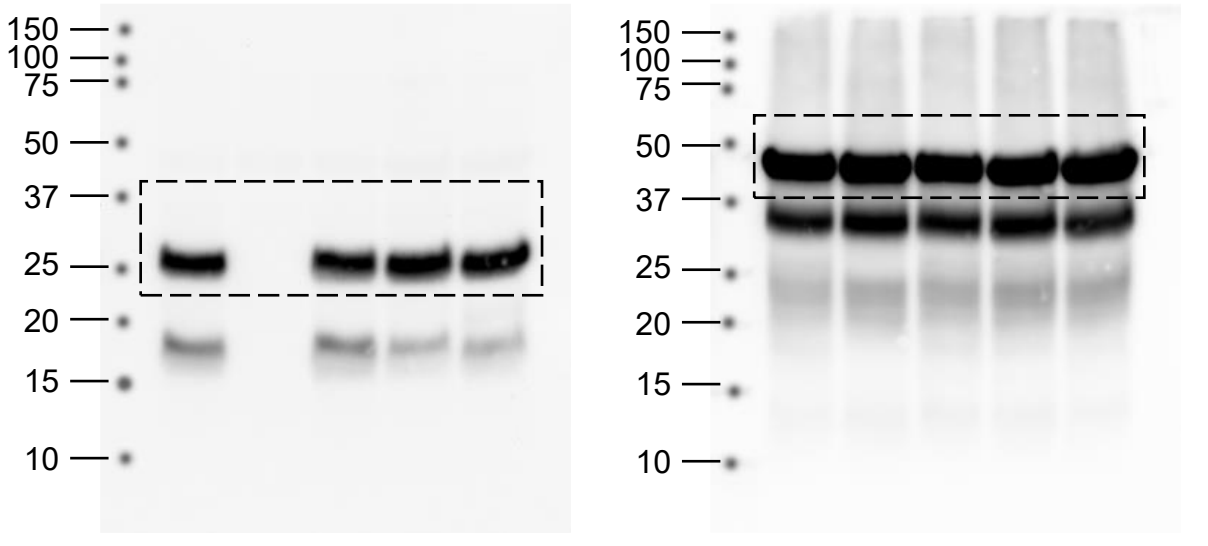

IB: α-HA-tag (Tim22)

IB: α-Pgk1

Both blots are from the same membrane.

Extended Data Fig. 4f

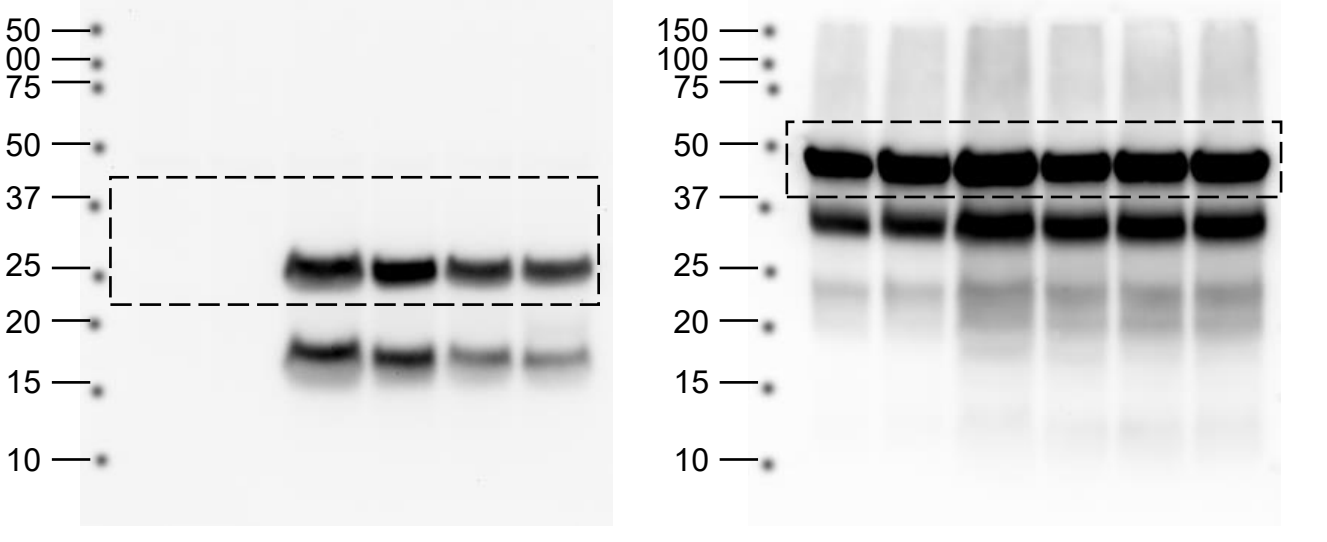

IB: α-HA-tag (Tim22)

IB: α-Pgk1

Both blots are from the same membrane.

Extended Data Fig. 4d

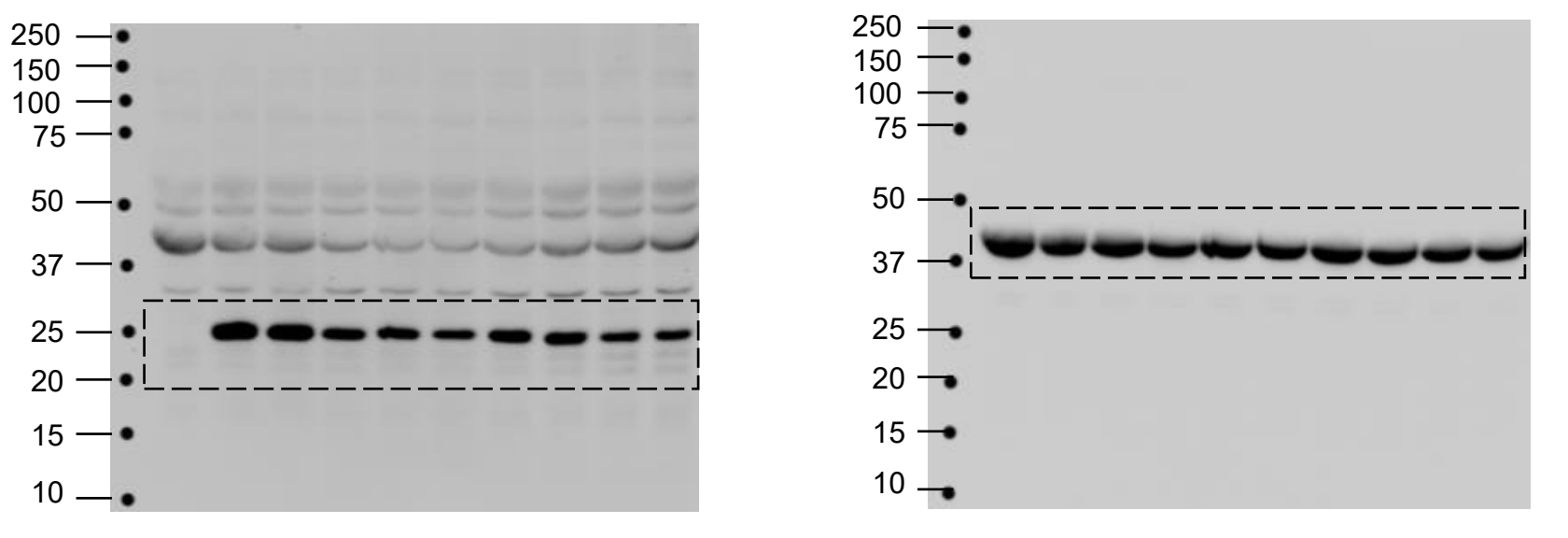

IB: α-HA-tag (Tim22)

IB: α-Pgk1

Both blots are from the same membrane.

Extended Data Fig. 4i

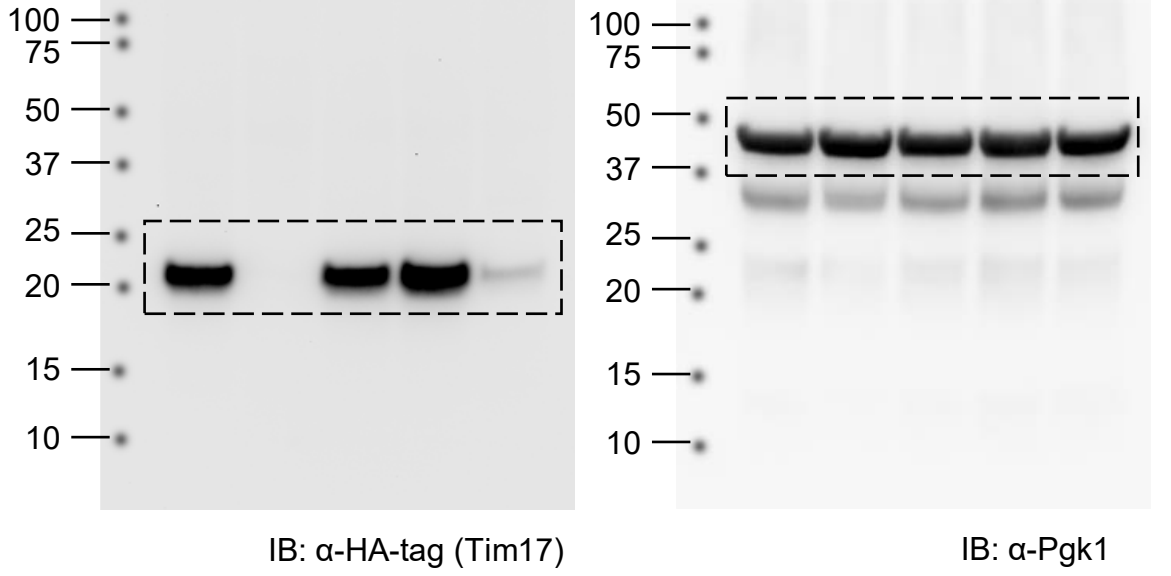

IB: α-HA-tag (Tim17)

IB: α-Pgk1

Both blots are from the same membrane.

Extended Data Fig. 5a

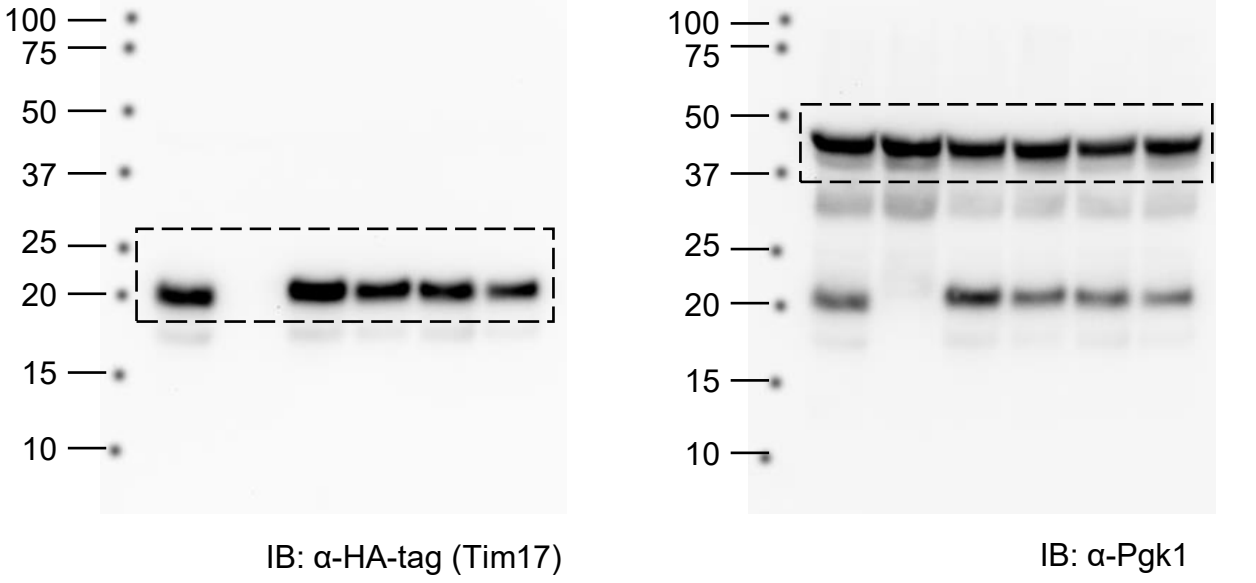

IB: α-HA-tag (Tim17)

IB: α-Pgk1

Both blots are from the same membrane.

Extended Data Fig. 5b

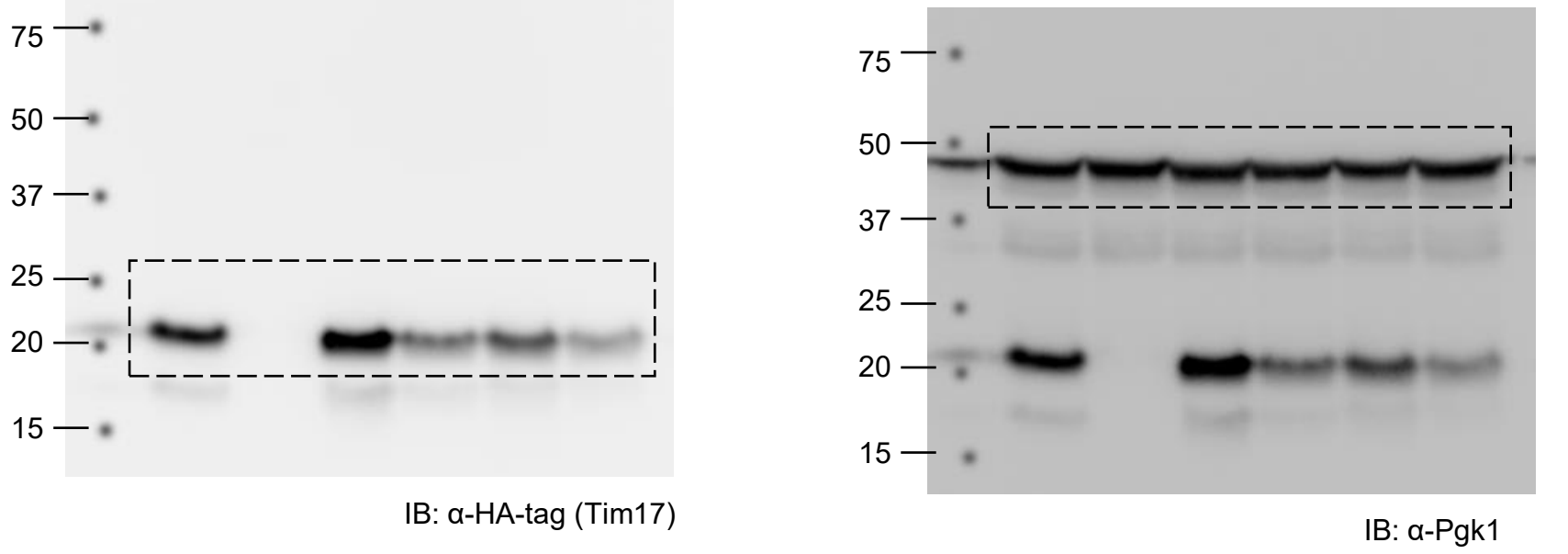

IB: α-HA-tag (Tim17)

IB: α-Pgk1

Both blots are from the same membrane.

Extended Data Fig. 5c

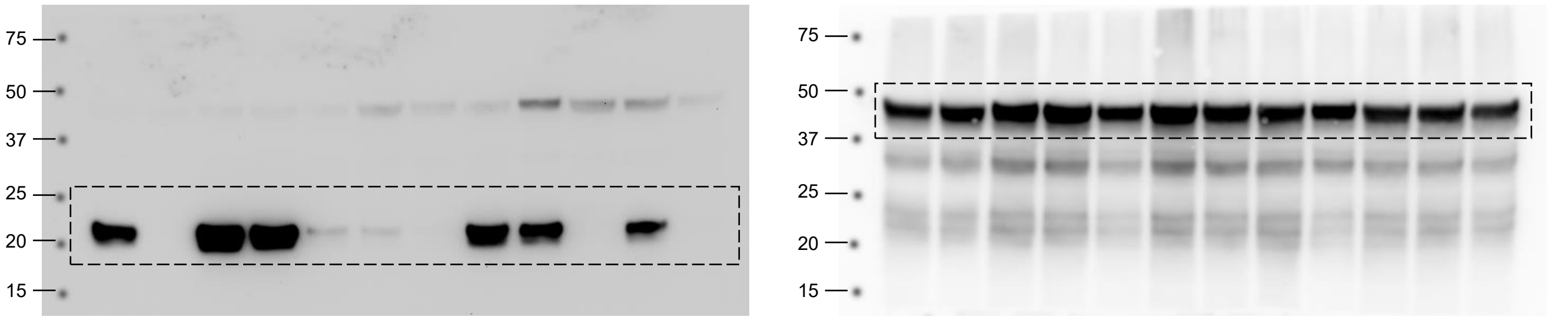

Both blots are from the same membrane.

Extended Data Fig. 5d

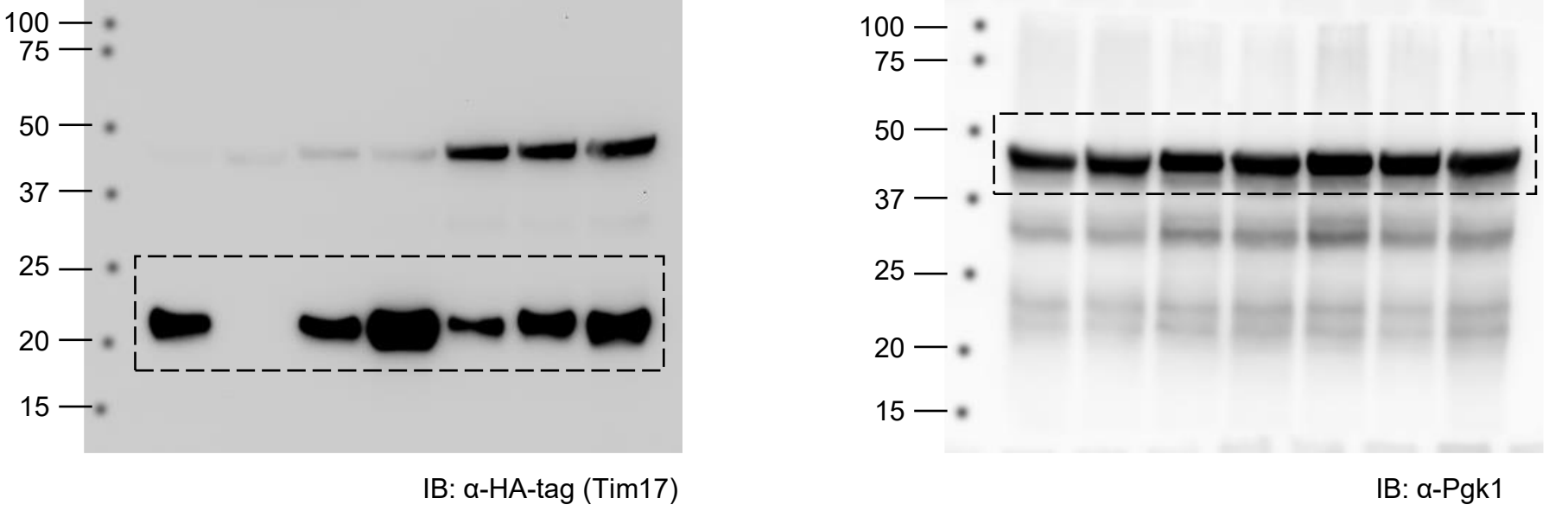

Both blots are from the same membrane.

Extended Data Fig. 5e

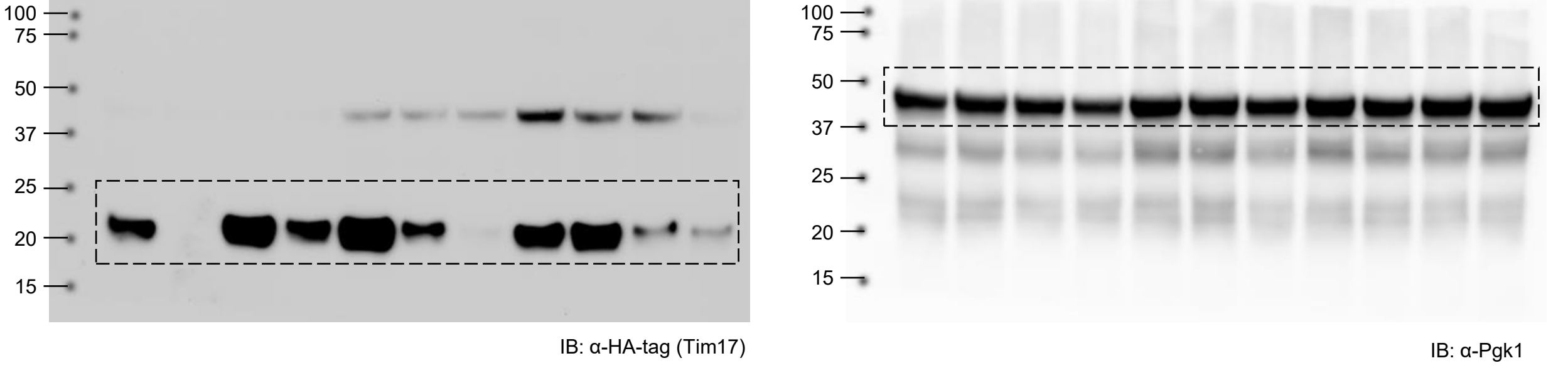

Both blots are from the same membrane.

Extended Data Fig. 9a

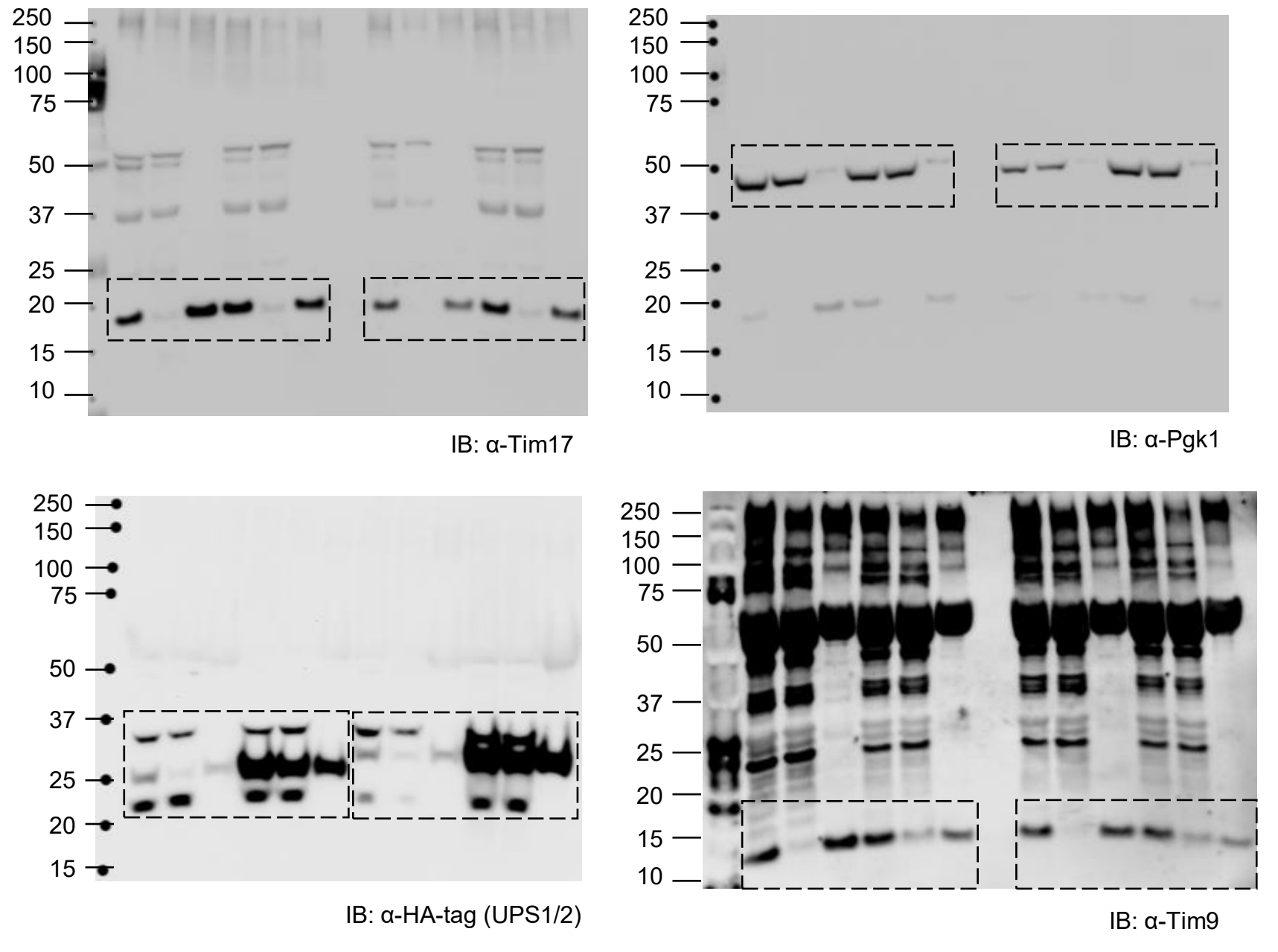

Anti-Tim17 and anti-Pgk1 blots are from the same gel/membrane.
Anti-HA and anti-Tim9 are from two separate gels loaded with equal volumes of the same samples in the same order.

Extended Data Fig. 9b

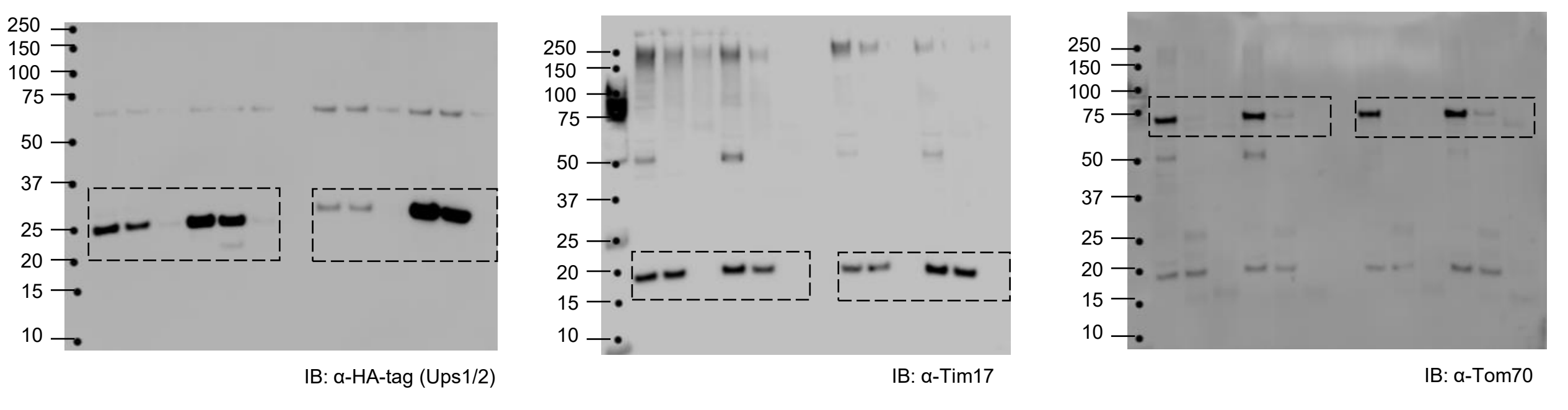

Anti-Tim17 and anti-Tom70 are from the same membrane.
Anti-HA blot is from a separate gel loaded with the equal volumes of the same samples.

Extended Data Fig. 9c

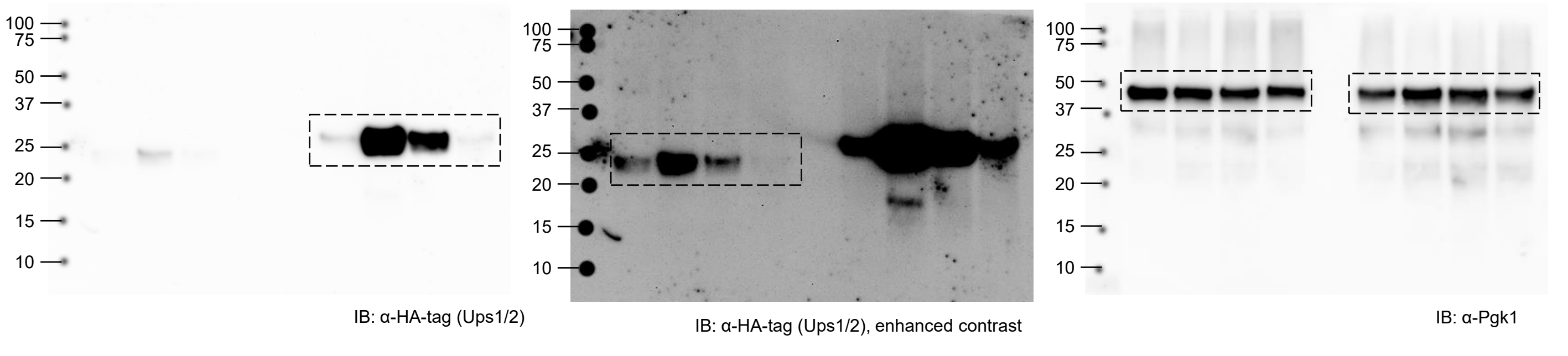

All blots are from the same membrane.
